# Expanding the mitochondrial genomic toolkit for Polyneoptera: New mitogenomes and evaluation of reduced marker sets for phylogeny and DNA barcoding

**DOI:** 10.1590/1678-4685-GMB-2025-0282

**Published:** 2026-07-24

**Authors:** Héctor Antônio Assunção Romão, Leonardo Carlos Jerônimo Corvalán, Juliana Alves Carneiro, David Daniel Ferreira dos Santos, Rhewter Nunes, Renata de Oliveira Dias

**Affiliations:** 1 Universidade Federal de Goiás, Instituto de Ciências Biológicas, Laboratório de Genética e Biodiversidade, Goiânia, GO, Brazil.; 2 Universidade Estadual de Goiás, Instituto Acadêmico de Ciências da Saúde e Biológicas, Laboratório de Bioinformática e Biodiversidade, Iporá, GO, Brazil.; 3 Pontifícia Universidade Católica de Goiás, Goiânia, GO, Brazil.

**Keywords:** Arthropoda, barcode, insecta, mitochondrial genome assembly, molecular marker

## Abstract

Polyneoptera comprises hemimetabolous insect orders of significant agricultural, ecological, and medical relevance, motivating phylogenetic and molecular research that has nevertheless focused predominantly on canonical mitochondrial markers. Here, we assembled new mitogenomes for Polyneoptera and evaluated the usefulness of genes located in nucleotide-diversity hotspots as markers for species identification and phylogenetic inference. To expand the available mitogenomic resources, raw sequencing data were retrieved from public databases, resulting in the assembly and annotation of 26 complete mitogenomes, all exhibiting the typical insect mitochondrial architecture. These newly assembled genomes were combined with publicly available mitogenomes from Orthoptera, Blattodea, Plecoptera, Mantodea, and Phasmatodea to reconstruct phylogenetic relationships using both complete and reduced datasets comprising nucleotide-diversity hotspot-associated genes. The performance of these hotspot regions was further assessed through barcoding gap analyses and comparisons with the most comprehensive datasets to identify candidate mitochondrial markers for molecular species identification and phylogenetic inference. Across orders, different mitochondrial regions, including the classical markers *16S* and *COX1*, as well as genes from the *NADH* dehydrogenase complex, emerged as the most informative, although optimal markers varied among lineages. Overall, our findings highlight the value of publicly accessible sequencing data for generating high-quality genomic resources and improving phylogenetic and taxonomic tools.

## Introduction

The superorder Polyneoptera comprises the orders Blattodea, Mantodea, Phasmatodea, Orthoptera, Plecoptera, Mantophasmatodea, Grylloblattodea, Embioptera, and Zoraptera. Species within this group occupy a wide range of ecological niches and exhibit remarkable morphological diversity, as well as diverse dietary habits and ecological behaviors (Wipfler et al., 2019). Moreover, Polyneoptera holds particular relevance for evolutionary and molecular identification studies, given that many of its species serve as indicators of water quality (Morinière et al., 2017), function as invasive urban pests or potential vectors of pathogens (Nasirian, 2017), or act as agricultural pests (Githae and Kuria, 2021).

The evolutionary aspects of Polyneoptera have been extensively investigated, and the monophyly of the group is well established, supported by both morphological and molecular evidence (Yoshizawa, 2011; Song et al., 2016; Wipfler et al., 2019). Among the molecular resources used in phylogenetic studies, the mitochondrial genome stands out as a widely applied and generally reliable marker for resolving intra-ordinal relationships and other evolutionary questions, owing to its conserved gene content and predictable mutation rates (Simon et al., 1994; Cameron, 2014). In Polyneoptera, mitochondrial data have been employed to infer phylogenetic relationships and to perform comparative analyses of genome organization across several orders, including Orthoptera (Zhang et al., 2013), Mantodea (Ma et al., 2023) , Blattodea (Cameron *et al*., 2012; Gong et al., 2018), Plecoptera (Ding et al., 2019), and Phasmatodea (Yuan et al., 2023).

However, the use of mitochondrial markers frequently relies on conventional gene sets rather than adopting a theory-driven approach to gene selection, such as focusing on the most variable regions to maximize phylogenetic signal (Whitfield and Kjer, 2008), and the usage of these highly variable regions, here referred to as hotspots, offers a promising alternative. Because these regions contain unconstrained sites with elevated variability, they provide critical information for distinguishing closely related taxa while reducing noise from non-informative positions (Simon et al., 1994). Hotspot-based datasets may therefore resolve phylogenetic relationships effectively without requiring complete mitochondrial genome sequencing.

In addition to their use in phylogenetic studies, mitochondrial markers are widely employed for species identification through DNA barcoding, primarily targeting regions of the cytochrome oxidase subunit I (*COX1*) gene, as well as other mitochondrial loci, in insects and other bilaterian taxa (Simon et al., 1994; Hebert et al., 2003a, b). However, exploring alternative candidate markers may yield both more specific primers (Moulton et al., 2010) and regions that exhibit well-defined patterns of intraspecific variation and interspecific divergence (Meyer and Paulay, 2005; Meier et al., 2008), thereby reducing error rates and minimizing misidentification inherited from co-amplification of NUMTs and intraspecific divergences (Dong et al. 2021).

Taken together, these observations highlight the underexplored potential of Polyneoptera mitogenomic data as a resource for assessing evolutionary patterns among orders and for species-level molecular identification. To address this gap and evaluate the applicability of mitochondrial genome regions for phylogenetic inference, as well as their potential utility as alternative or complementary barcode markers, we first expanded the available mitochondrial dataset by assembling and annotating high-quality, previously unavailable mitogenome sequences, then systematically investigated nucleotide-diverse regions, employing these hotspots to infer phylogenies and assess their suitability for both DNA barcoding and phylogenetic analyses. We hypothesized that a reduced set of carefully selected markers could achieve phylogenetic resolution comparable to that obtained with the most comprehensive datasets. To test this hypothesis, we compared the topologies reconstructed from these targeted mitochondrial regions with those obtained from complete mitogenomes and from datasets comprising all mitochondrial protein-coding genes (PCGs).

## Material and Methods

### Genome assembly, annotation, and quality assessment 

Whole Genome Shotgun (WGS) sequencing data obtained using the Illumina paired-end strategy for Blattodea, Mantodea, Phasmatodea, Plecoptera, and Orthoptera species without publicly available assembled mitogenomes (partial or complete) were retrieved from the NCBI Sequence Read Archive (SRA) database in March 2024. The search was performed using the superorder name as the term, filtering the results to retrieve only Illumina paired-end sequencing and prioritizing the most extensive library for each species according to the number of bases sequenced. The Polyneoptera orders: Mantophasmatodea, Grylloblattodea, Embioptera, and Zoraptera were not selected for inclusion in the present study due to insufficient information for further analysis, as none of these orders yielded more than five records in the RefSeq database. Sequencing reads were retrieved using the SRA Toolkit and then assembled *de novo* with NOVOPlasty 4.3.1 (Dierckxsens et al., 2016), using the protein-coding sequence of the *COX1* gene from a congeneric species as the seed for assembly extension, with k-mer values of 33 (Table S1). This strategy allowed a consistent mitogenome assembly with a high reliability due the seed-extension algorithm, reference guided assembly when available, and a high coverage due the WGS strategy used as filter to retrieve the sequence data. The completeness and integrality of the circularized contigs were inferred using AWA v.20170413, according to the values of the alignment score (Jacob Machado et al., 2018). 

The annotation was performed on the circularized mitogenomes using the MITOS2 web server (Bernt et al., 2013), following the Invertebrate Mitochondrial Code (transl_table=5). The protein-coding gene boundaries were manually verified using Ugene (Okonechnikov et al., 2012), in which the annotated genes were compared to those annotated in reference genomes retrieved from the RefSeq database by alignment in MAFFT, allowing the correction of start and stop codon positions (Katoh and Standley, 2013), this procedure allowed the identification of frameshifts, stop codon contamination and potentially NUMts.

To evaluate the quality of the assembled genomes, some metrics were established based on standard assumptions. First, the ratio of nonsynonymous to synonymous substitutions (dN/dS) was estimated for protein-coding genes using the codeML package implemented in the ETE Toolkit (Huerta-Cepas et al., 2016), applying M3 vs. M0 model to test for site-specific variability, with the expectation that ω values would remain low, as typically observed in mitochondrial genes. Second, nucleotide composition was examined by calculating AT content, AT-skew [(A − T) / (A + T)], and GC-skew [(G − C) / (G + C)], expecting the typical patterns for each order. Finally, relative synonymous codon usage (RSCU) was computed with CodonW v.1.4.4 to assess consistency with codon usage and composition patterns commonly reported in insect mitogenomes.

### Nucleotide diversity

To assess nucleotide diversity (π) across the 13 PCGs for each order, concatenated codon-sequence alignments were analysed using DnaSP v6.12.03 (Rozas et al., 2017). The analysis was conducted with a window length of 600 bp and a step size of 200 bp. The genes bearing regions of interest were identified through similarity searches using the Basic Local Alignment Search Tool (BLAST) against the NCBI non-redundant nucleotide database. Nucleotide diversity hotspots were defined as regions with π values greater than the median within the dataset. 

### Barcode evaluation

To identify the optimal mitochondrial regions for species-level identification in Polyneptera, we assessed the presence of a barcoding gap across nucleotide diversity hotspots detected in our dataset, as well as across commonly amplified mitochondrial markers reported in the literature. 

Scientific papers applying barcoding strategies for species identification in each Polyneoptera order analysed in this study were retrieved from the Web of Science database. The search used the following keywords: (“[ORDER]” AND (“DNA barcoding” OR “metabarcoding” OR “barcoding”)). Only studies that specified primer sets were included, and the corresponding primer sequences were also collected, along with the amplified gene regions (Table S2).

To calculate and visualize the DNA barcode gap for each order, a dataset comprising intraspecific and interspecific sequences was obtained from the NCBI nucleotide database. This was done using the *esearch* function from the Entrez package, whereby the species names with more than one record in the GenBank database were searched using the prompt: “SPECIES” AND (“8000”[SLEN] : “100000000000”[SLEN] AND (ddbj_embl_genbank[filter] AND mitochondrion[filter]). The search targeted both commonly amplified regions described in the literature and the hotspot regions identified in this study. To ensure the quality of the datasets, sequences retrieved for each order were aligned and conserved alignment blocks were extracted to remove non-homologous regions and sequences with unusually short or long lengths. To improve taxonomic consistency, only sequences identified at the species level were retained, excluding records with ambiguous annotations (e.g., “sp.”, “cf.”). In addition, an inspection of sequence similarity within each species dataset was performed to detect potential outliers. Pairwise genetic distances were calculated and UPGMA dendrograms were generated using similarity as the metric in the ape package (Paradis and Schliep, 2019). Sequences that did not cluster with other sequences of the same species were considered potential misidentifications or sequencing artifacts and were removed from the dataset. To ensure balanced comparisons between intra- and interspecific variation, only species represented by at least three sequences were included in the analyses, with the subspecies being treated as intraspecific records. The final curated dataset used in all analyses is available in Table S3.

These datasets were used to evaluate the effectiveness of the region for molecular species identification using the function barcoding.gap built in BarcodingR package (Zhang et al., 2017). Intraspecific and interspecific genetic distances were calculated using the Kimura 2-parameter (k2p) distance model. To further validate and substantiate the significance of the divergence between interspecific and intraspecific regions, the Mann-Whitney-Wilcoxon test was applied to the same dataset.

### Phylogenetic analysis

Phylogenetic trees for each taxonomic order were inferred using species from closely related orders as outgroups. Outgroups were selected as follows: Plecoptera: *Challia fletcheri* (Dermaptera: Pygidicranidae - NC_018538.1) and *Euborellia arcanum* (Dermaptera: Anisolabididae - NC_032075.1); Blattodea: *Hierodula membranacea* (Mantodea: Mantidae - NC_048984.1) and *Hierodula patellifera* (Mantodea: Mantidae - NC_034283.1); Orthoptera: *Sclerophasma paresisense* (Mantophasmatodea: Mantophasmatidae - NC_007701.1) and *Tamolanica tamolana* (Mantodea: Mantidae - NC_007702.1); Phasmatodea: *Epeorus unispinosus* (Ephemeroptera: Heptageniidae - NC_065800.1) and *Epeorus gibbus* (Ephemeroptera: Heptageniidae - NC_065799.1); Mantodea: *Sclerophasma paresisense* (Mantophasmatodea: Mantophasmatidae - NC_007701.1).

For each analyzed order, at least 11 datasets were constructed to evaluate the consistency of mitochondrial markers as phylogenetic resources. These datasets were defined as follows: 1) PCG: coding DNA sequence (CDS) of all protein-coding genes combined; 2) var: all CDS from protein-coding genes corresponding to the highly variable genes concatenated; 3) COX1_var: the CDS of *COX1* gene together with the CDS of identified hotspot genes; 4) PCG_Partition: all CDS of PCGs partitioned by gene; 5) COX1: the CDS of *COX1* region singularly; 6) mtDNA: the complete mitochondrial genome; 7) var_3rd: CDS of highly variable genes, excluding the third codon position; 8) COX1_3rd: only CDS of *COX1* gene, excluding the third codon position; 9) COX1_var_3rd: *COX1* and CDS of genes inside hotspot regions, excluding the third codon position; 10) PCG_3rd: all CDS of PCGs combined, excluding the third codon position; 11) GENE / GENE_3rd: each individual CDS of genes within a highly variable region, analyzed both as complete (GENE) and with the third codon position excluded (GENE_3rd). This design enabled assessment of the influence of gene selection and codon-position exclusion on phylogenetic congruence across the insect orders examined.

For datasets with PCGs, amino acid sequences were aligned using MAFFT v.7 with the L-INS-i strategy (Katoh and Standley, 2013). The amino acid alignments were then used to guide a codon-based sequence alignment using the Pal2Nal v. 14 Perl script (Suyama et al., 2006). The poorly aligned regions were removed from each alignment generated with trimAL v1.4 using the -automated1 option to determine the optimal mode for the dataset (Capella-Gutiérrez et al., 2009). For the multi-gene datasets, the sequence alignments were concatenated in a unique matrix using the catfasta2phyml script. For the complete mtDNA dataset, genomes were first standardized to start at the *ND2* gene by shifting the start position using MARS (Ayad and Pissis, 2017), and then aligned as described above. The *16S* sequences, in turn, were aligned directly without modification.

The maximum-likelihood phylogenetic tree was inferred using IQ-TREE v3 (Wong et al., 2025). The best-fit nucleotide substitution model was selected via the built-in ModelFinder Plus algorithm (Kalyaanamoorthy et al., 2017). Branch support was assessed with 1,000 bootstrap replicates.

To evaluate the performance of each dataset, the topologies of all inferred trees were compared pairwise using cophenetic distance matrices tested with a Mantel test, using the vegan package (Dixon, 2003), and symmetric Robinson-Foulds (RF) distances.

This study did not involve experiments on live vertebrates or human subjects. All data used were obtained from public databases and analyzed using computational approaches only.

## Results

### Characterization of the novel mitogenome sequences

**Figure 1-:**
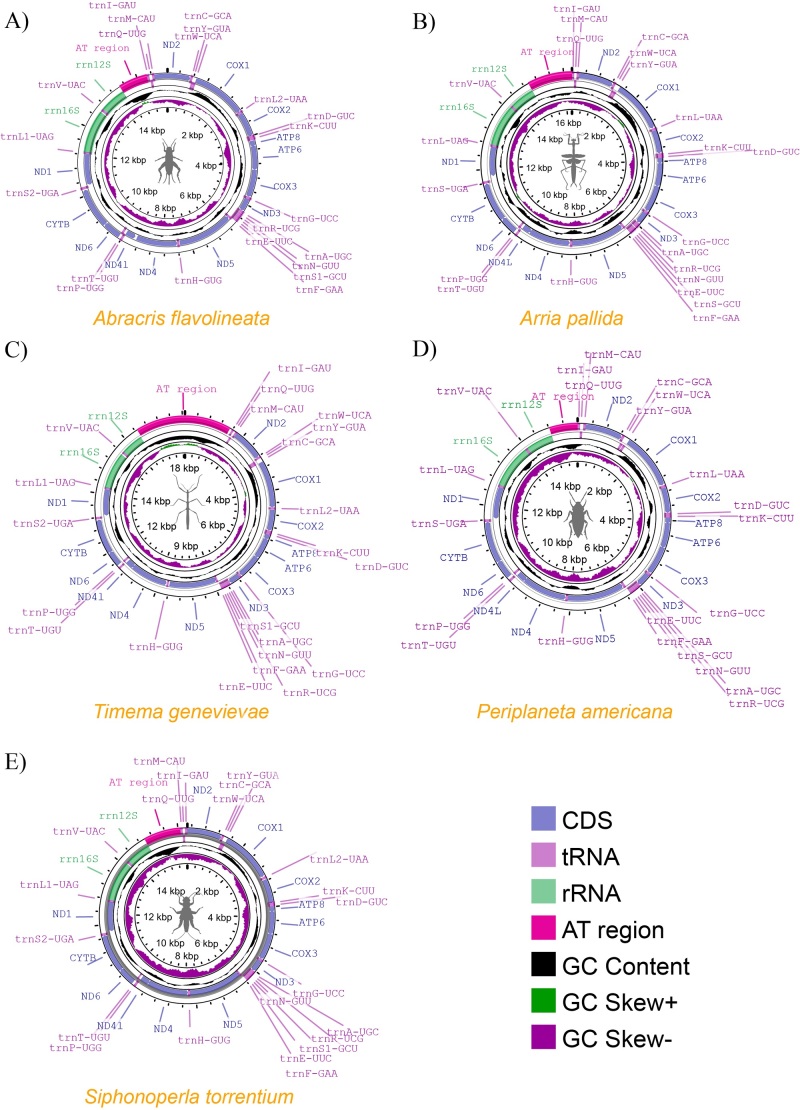
Circular maps of mitochondrial genomes from representatives of the Polyneoptera orders: (A) Abracris flavolineata (Orthoptera), (B) Arria pallida (Mantodea), (C) Timema genevievae (Phasmatodea), (D) Periplaneta americana (Blattodea), and (E) Siphonoperla torrentium (Plecoptera). The outer bars represent different types of genes and genomic features: blue for coding sequences (CDS), purple for transfer RNAs (tRNA), light green for ribosomal RNAs (rRNA), and magenta for the AT-rich region. The inner plots display the distribution of GC content (black), GC skew positive (green), and GC skew negative (purple).

We successfully obtained complete circularized mitogenome sequences for 26 novel representative species of Polyneoptera across 65 assembly attempts, yielding a 40% circularization rate. This includes nine species from Orthoptera, seven from Phasmatodea, and ten from Plecoptera. The assembled mitogenome lengths ranged from 15,532 bp in *Brachyptera seticornis* (Plecoptera: Taeniopterygidae) to 19,048 bp in *Timema bartmani* (Phasmatodea: Timematidae). Most mitogenomes followed the typical Metazoan gene composition, consisting of 37 genes (13 protein-coding genes, two rRNA genes, and 22 tRNA genes) and an AT-rich control region (Figure 1). The GC content of the mitogenomes varied from 22.41% in *Medauroidea extradentata* (Phasmatodea: Phasmatidae) to 37.38% in *Brachyptera seticornis* (Plecoptera: Taeniopterygidae), with a strong AT bias (Table S4).

The RSCU analysis showed a strong preference for codons with A or T in the third position (Table S5). The most frequently used codons were UUA (Leu), UCU (Ser), UCA (Ser), CGA (Arg), and GUU (Val). Among the least used codons, CGC (Arg) was the most commonly absent in the mitogenomes assembled, missing from the PCGs of *Podisma pedestris* (Orthoptera: Acrididae), *Ronderosia bergii* (Orthoptera: Acrididae), *Utaperla sopladora* (Plecoptera: Chloroperlidae), *Vandiemenella viatica* (Orthoptera: Morabidae), and *Xyleus discoideus angulatus* (Orthoptera: Romaleidae). This was followed by AGG (Ser), which was absent in *Amphinemura sulcicollis* (Plecoptera: Nemouridae), *Eyprepocnemis plorans* (Orthoptera: Acrididea), *Kathroperla siskiyou* (Plecoptera: Chloroperlidae), and *Utaperla lepnevae* (Plecoptera: Chloroperlidae), and GCG (Ala), which was not used in the PCGs of *Medauroidea extradentata*, *Pyrgomorpha conica* (Orthoptera: Acrididae), and *R. bergii* (Table S5). A similar relationship between the prevalence of AT codons and the dynamics of start and stop codons was also observed. The TAA stop codon was more prevalent than TAG, and ATA was more prevalent than ATG as a start codon in all species.

### Comparative analysis of mitogenomic characteristics across Polyneoptera orders

**Figure 2-:**
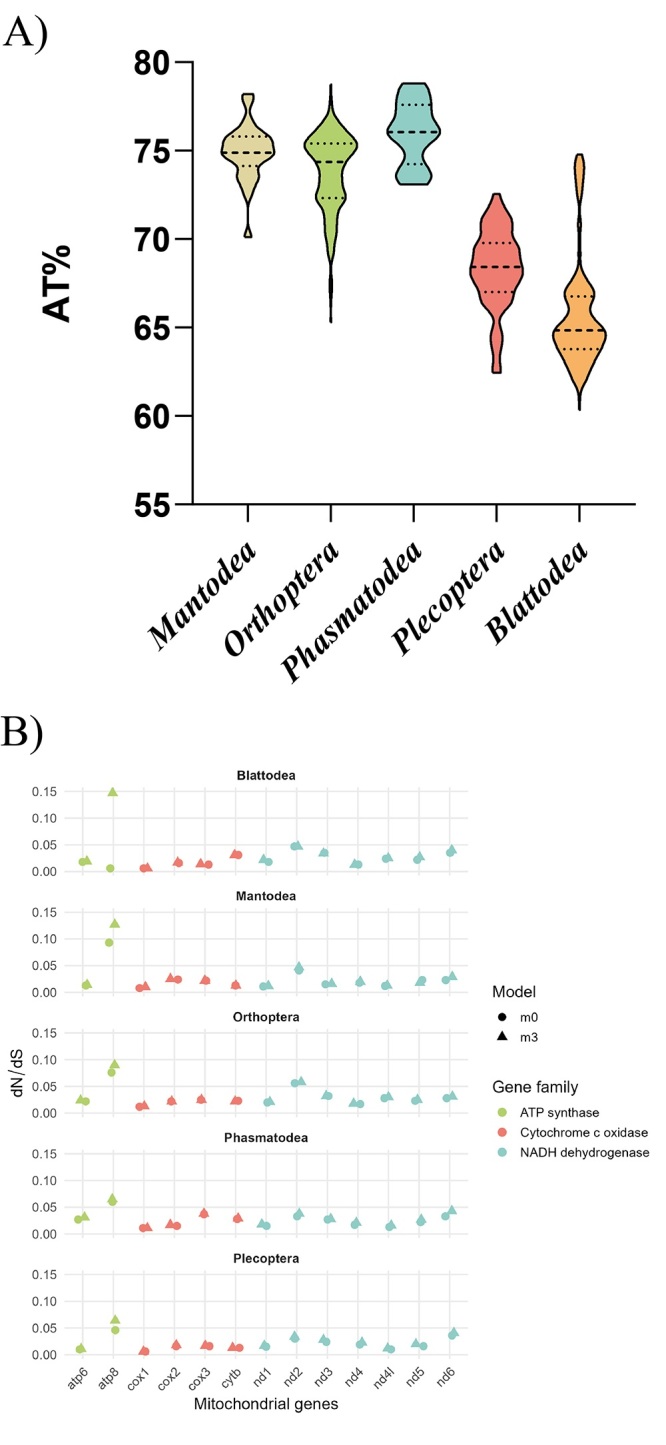
Mitochondrial genome characterization across Polyneoptera orders. (A) Distribution of AT content percentages among the mitochondrial genomes from each Polyneoptera order. (B) dN/dS ratios estimated for each mitochondrial protein-coding gene across the five analyzed orders, shown in separate panels. Gene families are indicated by different colors, and the two evolutionary models (m0 and m3) are represented by different symbols.

Among the five Polyneoptera orders analysed in this study, Phasmatodea exhibited the largest average mitogenome length at 16,996.94 ± 833.58 bp, while Blattodea had the smallest at 15,237.90 ± 565.24 bp. The variations in overall genome size were primarily due to differences in the non-coding portions, including intergenic and control regions (Table S6). All mitogenomes displayed a high AT content, exceeding 65% (Figure 2A).

The analysis of evolutionary pressure, assessed through the dN/dS ratio, revealed significant results for all genes in the dataset under the M3 vs. M0 model, which accounts for site-specific rate variation. Consistently low ω values were observed across all mitochondrial protein-coding genes, indicating strong purifying selection. The highest ω value was 0.147 for *ATP8* in Blattodea, and the lowest, 0.006, was found in *COX1* for both Blattodea and Plecoptera (Figure 2B).

### Nucleotide diversity across genes

**Figure 3-:**
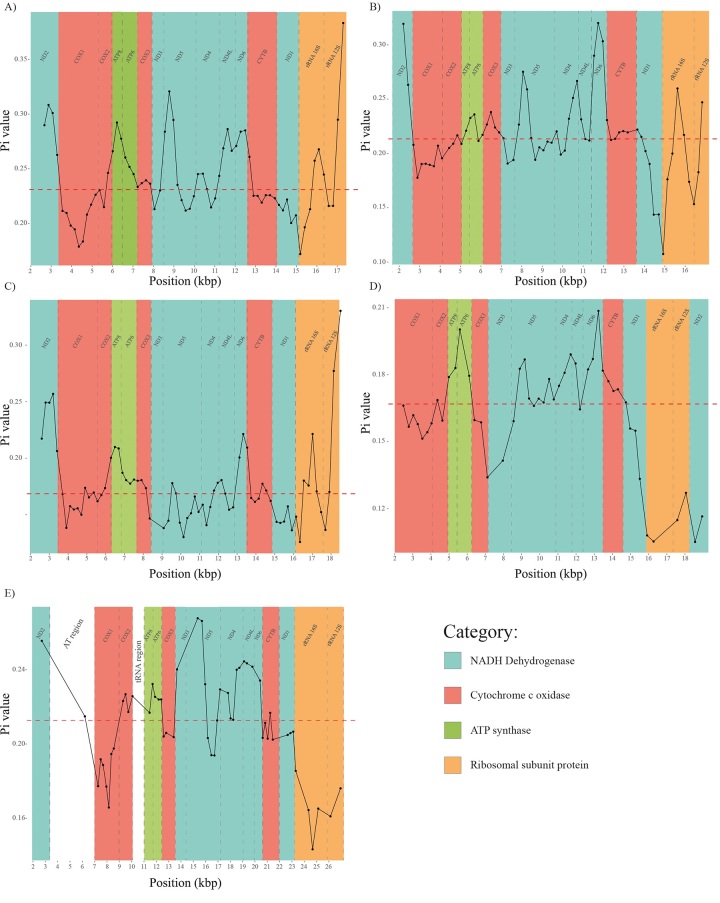
Nucleotide diversity across mitochondrial genomes in the five Polyneoptera orders analysed. (A) Phasmatodea, (B) Plecoptera, (C) Mantodea, (D) Blattodea, and (E) Orthoptera. The black lines represent the nucleotide diversity value across genome positions, and the colored blocks indicate gene categories: NADH dehydrogenase (blue), cytochrome c oxidase (red), ATP synthase (green), and ribosomal subunit proteins (orange).

The graphical distribution of nucleotide diversity across the genes of the orders Blattodea (average = 0.16, median = 0.17), Orthoptera (average = 0.21, median = 0.21), Mantodea (average = 0.17, median = 0.17), Phasmatodea (average = 0.24, median = 0.23), and Plecoptera (average = 0.22, median = 0.21) is illustrated in Figure 3. Notably, the nucleotide diversity values for three NADH-ubiquinone oxidoreductase (ND) subunits (*ND2*, *ND5*, and *ND6*) were consistently higher than the median across nearly all analysed orders. Specifically, *ND2* was identified as a hotspot in all orders except Blattodea, *ND5* in all but Mantodea, and *ND6* in all except Orthoptera. Additionally, the *ND4L* subunit emerged as a diversity hotspot in Orthoptera and Phasmatodea. Other notable hotspot genes included *ATP6* in Blattodea; *ATP8* in Orthoptera, Mantodea, and Phasmatodea; and *16S* in Mantodea and Plecoptera.

### Optimum primer region research

A total of 134 scientific articles describing barcoding applications in Polyneoptera species were identified through our Web of Science search. These articles encompassed all Polyneoptera orders included in our work, with the following distribution in descending order: Orthoptera (67), Plecoptera (42), Blattodea (15), Mantodea (9), and Phasmatodea (1). (Table S2). The publications included 62 distinct primer sets; the most frequently cited was the set described by Folmer et al. (1994) , referenced in 51 articles and used without modification in 43. The second most cited primer set was that described by Pan et al. (2006) , which was cited by 11 articles, but all from Orthoptera. 

The barcode regions amplified in the selected publications were, in descending order of frequency, from the genes: *COX1* (114), *16S* (8), *COX2* (4), *CYTB* (4), *12S* (2), *COX3* (1), and *ND2* (1). The suitability of these amplified regions, along with the hotspot regions identified through our nucleotide diversity analysis, was evaluated using barcoding gap analysis to assess the efficiency of these primers in distinguishing species from each order.

In the Blattodea dataset, only the hotspot regions of the *ND5* and *ND6* genes exhibited visible gaps, with genetic distances of 2.68% and 0.41%, respectively (Table S7). In contrast, the *COX1* gene, the most commonly used region for amplification showed no visible gaps. It presented a minimum interspecific distance of 0.0039 and a maximum intraspecific distance of 0.1713.

In Orthoptera, the regions displaying visible gaps corresponded to the predicted hotspot areas in the *ATP6* and *ND4* genes, with distances of 4.68% and 7.87%, respectively. As in Blattodea, the *COX1* region analysed in this study showed overlap between intraspecific and interspecific distance curves, with a minimum interspecific distance of 0.000.

In Plecoptera, visible gaps were predicted in all four hotspot regions analysed, located within the *ND4* (10.97%), *ND4L* (11.23%), *ND5* (25.55%), and *ND6* (12.41%) genes. However, as with the other orders, no visible gaps were observed in the *COX1* region tested. 

Mantodea was the only order in which all the tested regions, including *COX1*, exhibited visible gaps. The gaps between interspecific and intraspecific distances for this order were as follows: *16S* = 6.51%, *ATP8* = 9.53%, *COX1* = 7.40%, *ND2* = 4.84%, and *ND6* = 8.62%.

For Phasmatodea, only the region of *COX1* was deemed to contain sufficient information for analysis, although the minimum interspecific variance distance of 0.000 was recorded. The regions of the nucleotide diversity hotspots yielded at most two species per search and were therefore excluded. 

Despite the analyses conducted, none of the regions examined showed a high degree of similarity in the distribution of intraspecific and interspecific genetic distances, as assessed by the Mann-Whitney-Wilcoxon test (Table S7).

### Phylogenetic analysis

Each phylogenetic tree explored in this study was evaluated for its concordance with both the classical PCG dataset and the more informative complete mitochondrial genome (mtDNA) inferences, using the complementary metrics of Mantel test and Robinson-Foulds (RF) distances.

In Plecoptera, all datasets exhibited strong agreement with the phylogenies inferred from the most complete datasets based on Mantel correlations (values > 0.921) (Table S8). However, the RF test revealed greater variation in topological similarity across datasets, with the highest congruence observed when all hotspot genes were concatenated, either alone or with *COX1* (RF ≤ 0.222). However, the individual hotspot genes *ND4*, *16S*, *ND5*, *ND6*, and *COX3* performed better than *COX1* in the RF test.

For Orthoptera, the Mantel test revealed more variable outcomes, with *ND2* and *ND4L* showing the lowest concordance with phylogenies inferred from the most comprehensive datasets (Table S9). However, as in Plecoptera, datasets composed of highly variable genes, used either individually or in combination with *COX1*, showed the highest agreement with the complete mtDNA datasets. When comparing single-gene datasets, *ND5* slightly outperformed *COX1* in the RF test.

Across Phasmatodea, all datasets yielded highly similar results according to the Mantel test (Table S10). However, RF values revealed more informative differences: datasets combining the hotspot genes, either alone or together with *COX1*, and the *16S* gene alone, showed the closest agreement with the most comprehensive datasets. Moreover, as observed in Plecoptera, several hotspot genes used individually (*16S*, *ND5*, *ND4L*, and *ND2*) outperformed *COX1* alone in reproducing the phylogeny inferred from the complete datasets according to the RF test.

For Mantodea, all datasets yielded results comparable to the most comprehensive datasets, according to the Mantel test, except for *ATP8* when used alone (Table S11). However, the RF results revealed more evident differences: the closest topological agreement was obtained using the concatenated hotspot genes, either alone or combined with *COX1*, as well as with *16S* individually. Aside from *16S*, *COX1* performed better than the other hotspot genes when used alone.

Across Blattodea, all datasets performed similarly to the most comprehensive datasets, as indicated by the Mantel test (Table S12). However, the RF test revealed that concatenating all hotspot genes, either alone or in combination with *COX1*, yielded the closest topological agreement with the complete datasets. Additionally, in Blattodea, *ND2* and *ND5* outperformed *COX1* when used individually.

## Discussion

### Assembly results and quality assessment 

The assembly of mitogenomes using publicly available sequence archives successfully retrieved 26 novel polyneopteran mitochondrial genomes, including nine from Orthoptera, seven from Phasmatodea, and ten from Plecoptera. Notably, these genomes represent the first reported mitogenome sequences for 11 genera and two insect families, marking a significant contribution to the genomic resources available for these groups.

All mitogenomes assembled in this study exhibited the typical mitochondrial gene composition, consisting of 13 PCGs, 22 tRNAs, and two rRNAs, along with an AT-rich region (Boore, 1999). The high AT% bias observed in the assembled mitogenomes is consistent with previous findings for Polyneoptera groups, including Phasmatodea (Li et al., 2022), Plecoptera (Cao et al., 2024), Mantodea (Ma et al., 2023) , Blattodea (Cheng et al., 2016), and Orthoptera (Liu et al., 2013). This high AT% is partly explained by the presence of an AT-rich region, which optimizes the energy required for replication (Clary and Wolstenholme, 1987). This compositional bias is further influenced by the preferential use of the synonymous amino acids encoded by NNA and NNU codons, as observed in previous studies on other insect species (Wei et al., 2014; Barbhuiya et al., 2020). This can be attributed to the degeneracy of synonymous codons and the wobble position of the third base in the anticodon, which pairs more effectively with uracil-terminated codons (Sun et al., 2009). 

The genome skewness was positively correlated with AT content and negatively correlated with GC skew, as is typical of insect mitochondrial genomes. This pattern results from the strand-displacement model of replication, in which the parent minority strand is more susceptible to deamination, leading to an increase in A and C nucleotides on the lagging (majority) strand (Wei et al., 2010). The dN/dS analyses further support the quality and biological consistency of the assembled datasets, as ω values were uniformly low, indicating strong purifying selection, as typically observed in mitochondrial protein-coding genes. Although expected, this result confirms that both the newly assembled and curated mitogenomes follow canonical mitochondrial evolutionary patterns. Here, the M3 vs. M0 comparison was used as a diagnostic test for site-specific rate heterogeneity rather than to detect positive selection. The absence of anomalous ω values reinforces the reliability of the assemblies and supports their use in downstream hotspot detection, barcoding, and phylogenetic analyses.

### Performance of alternative markers to DNA barcoding

The nucleotide diversity hotspots identified in this study were most frequently located in regions of the *ND2*, *ND5*, and *ND6* genes. Among these, only the *ND2* region has previously been described for primer design in Polyneoptera, with a reported case in Orthoptera (Tokuda et al., 2010) and the *ND6* region amplification was also used in lepidopterans, yielding similar results to the conventional *COX1* marker (Silva-Brandão et al., 2011). It is noteworthy to mention that other regions of such *ATP6* and *ATP8* were already explored as alternative barcode loci in aphids (Lee and Akimoto, 2015). These nucleotide diversity hotspots serve as valuable markers for species identification, mainly since a lack of resolution in specific taxa is often attributed to factors such as saturation in mitochondrial markers, including *COX1*, incomplete lineage sorting, hybridization, and the presence of Nuclear-Mitochondrial DNA segments (NUMTs) (Kock et al., 2024). 

To evaluate the efficacy of the nucleotide diversity hotspot regions identified in our study compared to the frequently used barcode gene regions for species identification across the Polyneoptera orders analysed, we conducted a barcoding gap analysis. In these analyses, the *COX1* region was not deemed optimal for species delimitation due to the absence of a sufficiently large gap between intraspecific and interspecific divergence values, except in Mantodea, for which the region showed a gap of 7.40% between the maximum intraspecific and the minimum interspecific genetic distance, as well for the regions of *16S*, *ATP8*, *ND2* and *ND6*. For the remaining orders, other regions were predicted as optimal: *ND5* for Blattodea; *ND4* for Orthoptera; and *ND4*, *ND4L*, *ND5* and *ND6* for Plecoptera. 

Despite the identification of optimal regions for species identification, the absence of a barcode gap was primarily driven by the smoothing of intraspecific distance distributions, with most frequencies concentrated at low divergence levels. This pattern suggests that all evaluated regions remain promising candidates for species identification, even in the absence of a distinct barcode gap. This may also help explain why *COX1*, despite lacking a consistent barcode gap, remains the most frequently cited marker in our literature review and continues to be widely used, despite reported limitations for species delimitation in specific orders such as Orthoptera (Kock et al., 2024), Diptera (Whitworth et al., 2007), and Hymenoptera (Gerth et al., 2011). Notably, *COX1* remains useful for phylogenetic inference; in Orthoptera, for instance, it has been successfully employed to recover relationships using tree-based methods and distance thresholds (Huang et al., 2013). Nonetheless, the combined use of complementary approaches, such as similarity-based, diagnostic, and tree-based methods, offers a more robust framework for species delimitation (van Velzen *et al*., 2012).

Finally, it is essential to acknowledge that these results may benefit from re-evaluation as taxon sampling expands. The patterns observed here are likely influenced by the scale and composition of the currently available datasets. Notably, barcode gaps were detected only in datasets with fewer sampled species, which may capture fewer instances where coalescent depth obscures divergence patterns, an effect known to hinder the detection of apparent gaps (Collins and Cruickshank, 2013). As additional mitochondrial genomes become available, broader sampling may refine or modify these conclusions.

### Mitochondrial datasets for phylogenetic inferences benchmarking

The use of mitochondrial genomic resources to infer phylogenetic relationships in insects has traditionally focused on protein-coding gene (PCG) sequences, which comprise approximately 75% of the mitochondrial genome and thus provide substantial phylogenetic information (Cameron, 2014). Complete mitogenomes have also been widely employed, as they include non-coding regions that evolve more rapidly and may contribute additional phylogenetic signal (Mandal et al., 2014).

Our assessment of mitochondrial markers identified through nucleotide-diversity analyses and evaluated using the barcoding-gap approach yielded promising results for resolving phylogenetic relationships within Polyneoptera. However, no single marker proved universally informative across the entire superorder. This pattern is consistent with the pronounced mitochondrial divergence observed both within and among polyneopteran orders, particularly in terms of base composition (Cameron, 2014). As a result, analysing each order independently proved to be a more effective strategy for identifying informative markers and recovering robust phylogenetic signal. Moreover, it is important to note that although more variable mitochondrial regions may improve species discrimination, this typically comes at the cost of reduced primer universality. The widespread adoption of *COX1* as the standard barcode reflects a compromise between sequence conservation suitable for primer design and sufficient divergence for species-level identification (Folmer et al., 1994; Hebert et al., 2003a).

Among single-gene datasets from nucleotide-diversity hotspots, *ND4*, *16S*, *ND5*, *ND6*, and *COX3* outperformed the commonly used *COX1* in Plecoptera. *COX1* was also surpassed by *ND5* in Orthoptera; by *16S*, *ND5*, *ND4L*, and *ND2* in Phasmatodea; by *16S* in Mantodea; and by *ND2* and *ND5* in Blattodea. Taken together, these results show that although *COX1* remains a valuable resource for phylogenetic inference, single-gene analyses may benefit from selecting markers that are tailored to the target order. For example, the strong performance of several NADH dehydrogenase complex genes is consistent with previous studies demonstrating that NAD genes often provide robust phylogenetic signal across Metazoa. Their effectiveness has been attributed to both the large number of informative sites they contain and their distinct evolutionary rates, which together can yield clearer phylogenetic resolution (Havird and Santos, 2014).

However, despite the valuable insights obtained from single-gene analyses, none of them matched the performance of datasets composed of multiple concatenated genes. This reinforces the importance of assembling complete mitochondrial genomes for as many species as possible to achieve the highest phylogenetic resolution. Nevertheless, mitochondrial genomes have intrinsic limitations for resolving deep phylogenetic relationships, regardless of sampling effort, and are more reliable for shallow to intermediate evolutionary timescales.

In summary, we expanded the mitogenomic resources available for Polyneoptera by assembling 26 new high-quality mitochondrial genomes, including the first representatives for multiple genera and families. The assemblies displayed canonical mitochondrial architecture, consistent base-composition biases, and expected patterns of nucleotide skew, reinforcing their integrity and suitability for downstream analyses. These new genomes provide essential resources for improving molecular identification and evolutionary studies in this diverse and ecologically significant group.

Our evaluation of nucleotide-diversity hotspots and their applicability to species delimitation revealed that several mitochondrial regions hold strong potential as complementary or alternative markers to the widely used *COX1* gene. Although distinct optimal markers were identified for each order, no single locus exhibited a clear barcode gap across all datasets. This result reflects both intrinsic evolutionary characteristics of Polyneoptera and the limitations imposed by uneven taxon sampling. As mitogenomic sampling expands, the predictive power of these markers is expected to become more refined.

Benchmarking phylogenetic datasets further demonstrated that mitochondrial markers differ considerably in their informativeness across orders. While several NADH dehydrogenase genes and the ribosomal marker *16S* consistently outperformed *COX1* in single-gene phylogenetic analyses, the highest topological congruence was always achieved using multi-gene concatenated datasets. This order-specific performance, together with pronounced variation in mitochondrial base composition across Polyneoptera, underscores the importance of tailoring marker selection to the clade of interest rather than relying on a universal mitochondrial gene. Ultimately, our findings reaffirm the superior phylogenetic resolution afforded by complete mitogenome datasets and highlight the continued need to expand taxonomic coverage by assembling additional mitochondrial genomes.

Taken together, this work advances the phylogenetic and taxonomic toolkit available for Polyneoptera by: enriching the genomic repository for the group; identifying multiple informative regions for species identification; and providing a comparative framework that guides the selection of mitochondrial markers for phylogenetic inference. As new data accumulate, these insights will help refine mitochondrial-based approaches and support more robust evolutionary and biodiversity assessments across one of the most diverse insect lineages.

## Supplementary Material

The following online material is available for this article:

Table S1 - Summary of sequencing run information and genome assembly statistics for all species included in this study.

Table S2 - List of mitochondrial primers identified through a literature review for each taxonomic group analyzed, including primer sequences, original sources, and documented usage in Polyneoptera.

Table S3 - Accession number of the sequences used for the barcoding gap analysis.

Table S4 - Nucleotide composition of mitochondrial genomes from Polyneoptera species, including AT content, GC-skew, and AT-skew calculated for the complete genome and for different genomic partitions (protein-coding genes, rRNAs, and tRNAs).

Table S5 - Codon usage patterns across newly assembled mitochondrial genomes, showing relative synonymous codon usage (RSCU) values for protein-coding genes in representative Polyneoptera species.

Table S6 - Structural characteristics of mitochondrial genomes from Polyneoptera species analyzed in this study, including genome size, gene content, and lengths of coding and non-coding regions.

Table S7 - Results of barcoding gap analyses for Orthoptera, Blattodea, Mantodea, and Plecoptera based on mitochondrial gene regions. The table summarizes intraspecific and interspecific genetic distances (minimum, median, and maximum), the difference between minimum interspecific and maximum intraspecific distances, and the results of Mann-Whitney-Wilcoxon tests comparing distance distributions.

Table S8 - Mantel and Robinson-Foulds (RF) coefficients comparing phylogenetic trees inferred from different mitochondrial datasets in Plecoptera.

Table S9 - Mantel and Robinson-Foulds (RF) coefficients comparing phylogenetic trees inferred from different mitochondrial datasets in Orthoptera.

Table S10 - Mantel and Robinson-Foulds (RF) coefficients comparing phylogenetic trees inferred from different mitochondrial datasets in Phasmatodea.

Table S11 - Mantel and Robinson-Foulds (RF) coefficients comparing phylogenetic trees inferred from different mitochondrial datasets in Mantodea.

Table S12 - Mantel and Robinson-Foulds (RF) coefficients comparing phylogenetic trees inferred from different mitochondrial datasets in Blatoddea.

## Data Availability

 Nucleotide sequence data reported are available in the Third Party Annotation Section of the DDBJ/ENA/GenBank databases under the accession numbers: BK068635, BK068636, BK068638, BK068639, BK068643-BK068652, BK068655-BK068665.

## References

[B1] Ayad LAK, Pissis SP (2017). MARS: Improving multiple circular sequence alignment using
refined sequences. BMC Genomics.

[B2] Barbhuiya RI, Uddin A, Chakraborty S (2020). Codon usage pattern and its influencing factors for mitochondrial
CO genes among different classes of Arthropoda. Mitochondrial DNA A DNA Mapp Seq Anal.

[B3] Bernt M, Donath A, Jühling F, Externbrink F, Florentz C, Fritzsch G, Pütz J, Middendorf M, Stadler PF (2013). MITOS: Improved de novo metazoan mitochondrial genome
annotation. Mol Phylogenet Evol.

[B4] Boore JL (1999). Animal mitochondrial genomes. Nucleic Acids Res.

[B5] Cameron SL (2014). Insect Mitochondrial Genomics: Implications for evolution and
phylogeny. Annu Rev Entomol.

[B6] Cameron SL, Lo N, Bourguignon T, Svenson GJ, Evans TA (2012). A mitochondrial genome phylogeny of termites (Blattodea:
Termitoidae): Robust support for interfamilial relationships and molecular
synapomorphies define major clades. Mol Phylogenet Evol.

[B7] Cao J-J, Wang Y, Murányi D, Cui J-X, Li W-H (2024). Mitochondrial genomes provide insights into the Euholognatha
(Insecta: Plecoptera). BMC Ecol Evo.

[B8] Capella-Gutiérrez S, Silla-Martínez JM, Gabaldón T (2009). trimAl: A tool for automated alignment trimming in large-scale
phylogenetic analyses. Bioinformatics.

[B9] Cheng X-F, Zhang L-P, Yu D-N, Storey KB, Zhang J-Y (2016). The complete mitochondrial genomes of four cockroaches (Insecta:
Blattodea) and phylogenetic analyses within cockroaches. Gene.

[B10] Clary DO, Wolstenholme DR (1987). Drosophila mitochondrial DNA: Conserved sequences in the A+T-rich
region and supporting evidence for a secondary structure model of the small
ribosomal RNA. J Mol Evol.

[B11] Collins RA, Cruickshank RH (2013). The seven deadly sins of DNA barcoding. Mol Ecol Resour.

[B12] Dixon P (2003). VEGAN, a package of R functions for community
ecology. J Veg Sci.

[B13] Dierckxsens N, Mardulyn P, Smits G (2016). NOVOPlasty: De novo assembly of organelle genomes from whole
genome data. Nucleic Acids Res.

[B14] Ding S, Li W, Wang Y, Cameron SL, Murányi D, Yang D (2019). The phylogeny and evolutionary timescale of stoneflies (Insecta:
Plecoptera) inferred from mitochondrial genomes. Mol Phylogenet Evol.

[B15] Dong Z, Wang Y, Li C, Li L, Men X (2021). Mitochondrial DNA as a molecular marker in insect ecology:
Current status and future prospects. Ann Entomol Soc Am.

[B16] Folmer O, Black M, Hoeh W, Lutz R, Vrijenhoek R (1994). DNA primers for amplification of mitochondrial cytochrome c
oxidase subunit I from diverse metazoan invertebrates. Mol Mar Biol Biotechnol.

[B17] Gerth M, Geißler A, Bleidorn C (2011). Wolbachia infections in bees (Anthophila) and possible
implications for DNA barcoding. Syst Biodivers.

[B18] Githae EW, Kuria EK (2021). Biological control of desert locust (Schistocerca gregaria
Forskål). CABI Rev.

[B19] Gong R, Guo X, Ma J, Song X, Shen Y, Geng F, Price M, Zhang X, Yue B (2018). Complete mitochondrial genome of Periplaneta brunnea (Blattodea:
Blattidae) and phylogenetic analyses within Blattodea. J Asia Pac Entomol.

[B20] Havird JC, Santos SR (2014). Performance of single and concatenated sets of mitochondrial
genes at inferring metazoan relationships relative to full mitogenome
data. PLoS One.

[B21] Hebert PDN, Cywinska A, Ball SL, deWaard JR (2003a). Biological identifications through DNA barcodes. Proc R Soc Lond B.

[B22] Hebert PDN, Ratnasingham S, De Waard JR (2003). Barcoding animal life: Cytochrome c oxidase subunit 1 divergences
among closely related species. Proc R Soc Lond B.

[B23] Huang J, Zhang A, Mao S, Huang Y (2013). DNA barcoding and species boundary delimitation of selected
species of Chinese Acridoidea (Orthoptera: Caelifera). PLoS One.

[B24] Huerta-Cepas J, Serra F, Bork P (2016). ETE 3: Reconstruction, analysis, and visualization of
phylogenomic data. Mol Biol Evol.

[B25] Jacob Machado D, Janies D, Brouwer C, Grant T (2018). A new strategy to infer circularity applied to four new complete
frog mitogenomes. Ecol Evol.

[B26] Kalyaanamoorthy S, Minh BQ, Wong TKF, Von Haeseler A, Jermiin LS (2017). ModelFinder: Fast model selection for accurate phylogenetic
estimates. Nat Methods.

[B27] Katoh K, Standley DM (2013). MAFFT Multiple sequence alignment software version 7:
Improvements in performance and usability. Mol Biol Evol.

[B28] Kock L-S, Körs E, Husemann M, Davaa L, Dey L-S (2024). Barcoding fails to delimit species in Mongolian Oedipodinae
(Orthoptera, Acrididae). Insects.

[B29] Lee W, Akimoto S-I (2015). Development of new barcoding loci in gall-forming aphids
(Eriosomatinae: Eriosomatini): Comparing three mitochondrial genes, ATP6,
ATP8, and COI. J Asia Pac Entomol.

[B30] Li Y, Wang S, Chen J, Zhou J, Bu W (2022). Two new stick insect species of Sosibia Stål (Phasmatodea:
Lonchodidae: Necrosciinae) from China and the first report on mitochondrial
genomes of this genus. Arch Insect Biochem Physiol.

[B31] Liu C, Chang J, Ma C, Li L, Zhou S (2013). Mitochondrial genomes of two Sinochlora species (Orthoptera):
novel genome rearrangements and recognition sequence of replication
origin. BMC Genomics.

[B32] Ma Y, Zhang L, Lin Y, Yu D, Storey KB, Zhang J (2023). Phylogenetic relationships and divergence dating of Mantodea
using mitochondrial phylogenomics. Syst Entomol.

[B33] Mandal SD, Chhakchhuak L, Gurusubramanian G, Kumar NS (2014). Mitochondrial markers for identification and phylogenetic studies
in insects - A Review. DNA Barcodes.

[B34] Meier R, Zhang G, Ali F (2008). The use of mean instead of smallest interspecific distances
exaggerates the size of the “barcoding gap” and leads to
misidentification. Syst Biol.

[B35] Meyer CP, Paulay G (2005). DNA barcoding: Error rates based on comprehensive
sampling. PLoS Biol.

[B36] Morinière J, Hendrich L, Balke M, Beermann AJ, König T, Hess M, Koch S, Müller R, Leese F, Hebert PDN (2017). A DNA barcode library for Germany’s mayflies, stoneflies and
caddisflies (Ephemeroptera, Plecoptera and Trichoptera). Mol Ecol Resour.

[B37] Moulton MJ, Song H, Whiting MF (2010). Assessing the effects of primer specificity on eliminating numt
coamplification in DNA barcoding: A case study from Orthoptera (Arthropoda:
Insecta). Mol Ecol Resour.

[B38] Nasirian H (2017). Infestation of cockroaches (Insecta: Blattaria) in the human
dwelling environments: A systematic review and meta-analysis. Acta Trop.

[B39] Okonechnikov K, Golosova O, Fursov M, UGENE Team (2012). Unipro UGENE: A unified bioinformatics toolkit. Bioinformatics.

[B40] Paradis E, Schliep K (2019). ape 5.0: An environment for modern phylogenetics and evolutionary
analyses in R. Bioinformatics.

[B41] Pan CY, Hu J, Zhang X, Huang Y (2006). The DNA barcoding application of mtDNA COI gene in seven species
of Catantopidae (Orthoptera). Entomotaxonomia.

[B42] Rozas J, Ferrer-Mata A, Sánchez-DelBarrio JC, Guirao-Rico S, Librado P, Ramos-Onsins SE, Sánchez-Gracia A (2017). DnaSP 6: DNA sequence polymorphism analysis of large data
sets. Mol Biol Evol.

[B43] Silva-Brandão KL, Lyra ML, Santos TV, Seraphim N, Albernaz KC, Pavinato VAC, Martinelli S, Cônsoli FL, Omoto C (2011). Exploitation of mitochondrial nad6 as a complementary marker for
studying population variability in Lepidoptera. Genet Mol Biol.

[B44] Simon C, Frati F, Beckenbach A, Crespi B, Liu H, Flook P (1994). Evolution, weighting, and phylogenetic utility of mitochondrial
gene sequences and a compilation of conserved polymerase chain reaction
primers. Ann Entomol Soc Am.

[B45] Song N, Li H, Song F, Cai W (2016). Molecular phylogeny of Polyneoptera (Insecta) inferred from
expanded mitogenomic data. Sci Rep.

[B46] Sun Z, Wan D-G, Murphy RW, Ma L, Zhang X-S, Huang D-W (2009). Comparison of base composition and codon usage in insect
mitochondrial genomes. Genes Genom.

[B47] Suyama M, Torrents D, Bork P (2006). PAL2NAL: Robust conversion of protein sequence alignments into
the corresponding codon alignments. Nucleic Acids Res.

[B48] Tokuda M, Tanaka S, Zhu D-H (2010). Multiple origins of Locusta migratoria (Orthoptera: Acrididae) in
the Japanese Archipelago and the presence of two major clades in the world:
Evidence from a molecular approach. Biol J Linn Soc Lond.

[B49] Van Velzen R, Weitschek E, Felici G, Bakker FT (2012). DNA barcoding of recently diverged species: Relative performance
of matching methods. PLoS One.

[B50] Wei L, He J, Jia X, Qi Q, Liang Z, Zheng H, Ping Y, Liu S, Sun J (2014). Analysis of codon usage bias of mitochondrial genome in Bombyx
mori and its relation to evolution. BMC Evol Biol.

[B51] Wei S-J, Shi M, Chen X-X, Sharkey MJ, Van Achterberg C, Ye G-Y, He J-H (2010). New views on strand asymmetry in insect mitochondrial
genomes. PLoS One.

[B52] Whitfield JB, Kjer KM (2008). Ancient rapid radiations of insects: Challenges for phylogenetic
analysis. Annu Rev Entomol.

[B53] Whitworth TL, Dawson RD, Magalon H, Baudry E (2007). DNA barcoding cannot reliably identify species of the blowfly
genus Protocalliphora (Diptera: Calliphoridae). Proc R Soc B.

[B54] Wipfler B, Letsch H, Frandsen PB, Kapli P, Mayer C, Bartel D, Buckley TR, Donath A, Edgerly-Rooks JS, Fujita M (2019). Evolutionary history of Polyneoptera and its implications for our
understanding of early winged insects. Proc Natl Acad Sci U S A.

[B55] Wong T, Ly-Trong N, Ren H, Baños H, Roger A, Susko E, Bielow C, De Maio N, Goldman N, Hahn M (2025). IQ-TREE 3: Phylogenomic inference software using complex
evolutionary models. Mol Biol Evol.

[B56] Yoshizawa K (2011). Monophyletic Polyneoptera recovered by wing base
structure. Syst Entomol.

[B57] Yuan Y, Zhang L, Li K, Hong Y, Storey KB, Zhang J, Yu D (2023). Nine mitochondrial genomes of phasmatodea with two novel
mitochondrial gene rearrangements and phylogeny. Insects.

[B58] Zhang A, Hao M, Yang C, Shi Z (2017). BarcodingR: An integrated r package for species identification
using DNA barcodes. Methods Ecol Evol.

[B59] Zhang H-L, Huang Y, Lin L-L, Wang X-Y, Zheng Z-M (2013). The phylogeny of the Orthoptera (Insecta) as deduced from
mitogenomic gene sequences. Zool Stud.

