# Supplementary material for: Expanding the mitochondrial genomic toolkit for Polyneoptera: New mitogenomes and evaluation of reduced marker sets for phylogeny and DNA barcoding
Source: Genet Mol Biol. 2026 Jul 24;49(3):e20250282. doi: 10.1590/1678-4685-GMB-2025-0282 (PMC13403772; doi:10.1590/1678-4685-GMB-2025-0282)
Supplement: Table S1 - [file 1415-4757-GMB-49-3-e20250282-s1.pdf]

Genetics and Molecular Biology

DOI: https://doi.org/10.1590/1678-4685-GMB-2025-0282

Supplementary Material to "Expanding the mitochondrial genomic toolkit for Polyneoptera: New mitogenomes and evaluation of reduced marker sets for phylogeny and DNA barcoding"

Table S1 - Summary of sequencing run information and genome assembly statistics for all species included in this study.

	Sequence reads		Seed	Assembly statistics			
**Species name**	**NCBI SRA Accession number**	**Total bases (bp)**	**NCBI Accession Number**	**Status**	**#Contigs**	**Largest contig**	**Coverage**
Abracris flavolineata	SRR16892668	1025958202	MG993440	circularized	1	15668	474
Aeropedellus variegatus	SRR10395434	422051592	MG470492.1	fail	NA	NA	NA
Amphinemura sulcicollis	SRR3262386	11958688110	MH085447.1	circularized	1	15829	1960
Anisolabis maritima	SRR8064813	24327861600	MZ701626.1	incomplete	1	7482	NA
Brachyptera seticornis	SRR15358142	19920989088	JQ736345.1	circularized	1	15532	7385
Burttia prunoides	ERR7618047	934940100	NC_057646.1	incomplete	1	2100	NA
Chorthippus biguttulus	SRR10398078	172852629	ON422093.1	fail	NA	NA	NA
Clitarchus hookeri	SRR5889587	125823423200	MW530386.1	incomplete	122	9451	NA
Doru luteipes	SRR5651483	1344851564	OR922644.1	fail	NA	NA	NA
Eumigus monticolus	SRR3000673	5071063752	AM267143.1	circularized	1	15627	667
Eyprepocnemis plorans	SRR2970625	8107209602	MG993425.1	circularized	1	15668	1583
Forficula auricularia	SRR17839760	241487073442	MT072862.1	fail	NA	NA	NA
Hierodula sp. WJ-2021	SRR16955305	3150183600	NC_048984.1	incomplete	3	15312	NA
Hymenopus oblongifolius	ERR7618015	206099598	KX434861.1	incomplete	1	3279	NA
Kathroperla doma	SRR13743954	25326267923	MG382590.1	circularized	1	15990	3196
Kathroperla siskiyou	SRR13743953	17170194062	MG382590.1	circularized	1	16358	869
Leuctra hippopus	SRR11470105	2736462166	MK568475.1	circularized	1	15685	207
Leuctra nigra	SRR12518772	3319907442	MK568475.1	incomplete	33	7802	NA
Medauroidea extradentata	SRR6383867	177869241000	ON881016.1	circularized	1	17428	13698
Paraperla wilsoni	SRR13743952	21580052766	MH840645.1	circularized	1	16594	602
Pholidoptera griseoaptera	SRR12432320	3459266550	MW243136.1	incomplete	9	7202	NA
Phryganogryllacris superangulata	SRR23815002	21898708800	NC_033994.1	incomplete	5	8477	NA

Species name	Sequence reads		Seed	Assembly statistics			
	NCBI SRA Accession number	Total bases (bp)	NCBI Accession Number	Status	#Contigs	Largest contig	Coverage
Podisma pedestris	SRR18000087	1440276608	MT311125.1	circularized	1	15627	166
Pseudochorthippus parallelus	SRR10398080	200579291	OR780552.1	incomplete	1	3044	NA
Pyrgomorpha conica	SRR3953136	5364985872	KM384853.1	circularized	1	15636	296
Ronderosia bergii	SRR11819620	2415130584	MT771674.1	circularized	1	15606	375
Schistocerca americana	SRR16603599	135330327736	NC_013240.1	incomplete	4	306	NA
Schistocerca cancellata	SRR18836884	210887592976	NC_013240.1	incomplete	1	355	NA
Schistocerca ceratiola	SRR14493068	592008612	NC_013240.1	incomplete	1	3544	NA
Schistocerca damnifica	SRR14493059	1875726832	NC_013240.1	incomplete	4	802	NA
Schistocerca flavofasciata	SRR14493067	1189871846	NC_013240.1	fail	NA	NA	NA
Schistocerca nitens	SRR19025522	142335334610	NC_013240.1	incomplete	2	2059	NA
Schistocerca nitens caribbeana	SRR14493065	1646719024	NC_013240.1	fail	NA	NA	NA
Schistocerca pallens	SRR14493066	1170105040	NC_013240.1	incomplete	1	1740	NA
Schistocerca piceifrons	SRR16604496	127129483006	NC_013240.1	incomplete	141	1941	NA
Schistocerca rubiginosa	SRR14493060	1664962542	NC_013240.1	incomplete	2	1457	NA
Schistocerca serialis cubense	SRR18836885	177844456860	NC_013240.1	incomplete	1	252	NA
Sigaus australis	SRR1575334	54989420	EF544562.1	fail	NA	NA	NA
Sigaus childi	SRR1575319	92933370	EF544562.1	fail	NA	NA	NA
Siphonoperla torrentium	SRR12777459	32948649900	MT482900.1	circularized	1	15996	3866
Stauroderus scalaris	SRR10398081	170680097	MT311127.1	incomplete	2	2288	NA
Stenopelmatus talpa	SRR18074866	234262200	NC_028058.1	incomplete	1	297	NA
Stenopelmatus typhlops	SRR18074868	193439100	NC_028058.1	incomplete	1	753	NA
Tachycines shuangcha	SRR18390613	10280297100	NC_057442.1	fail	NA	NA	NA
Taeniopteryx nebulosa	SRR12518807	2441110092	NC_037897.1	incomplete	1	15314	NA
Tenodera sp. WJ-2021	SRR18218046	3617209800	MN447996.1	incomplete	5	15549	NA
Timema bartmani	SRR5248893	24540161700	DQ241799.1	circularized	1	19048	3541
Timema cristinae	SRR6345715	177827595716	DQ241799.1	incomplete	21	4984	NA
Timema douglasi	SRR18079125	32035918702	DQ241799.1	fail	NA	NA	NA
Timema genevievae	SRR5248887	28312966800	DQ241799.1	circularized	1	18085	4721
Timema monikensis	SRR5248911	33563060100	DQ241799.1	circularized	1	16977	11373
Timema podura	SRR14340209	3646615504	DQ241799.1	circularized	1	16864	880
Timema poppensis	SRR5248929	29584773300	DQ241799.1	circularized	1	18305	1924
Timema shepardi	SRR5248923	29843238900	DQ241799.1	circularized	1	17174	3354

Species name	Sequence reads		Seed	Assembly statistics			
	NCBI SRA Accession number	Total bases (bp)	NCBI Accession Number	Status	#Contigs	Largest contig	Coverage
Timema tahoe	SRR5248899	25423391700	DQ241799.1	incomplete	2	18493	NA
Utaperla gaspesiana	SRR13743951	19585467342	EF623003.1	circularized	1	17119	2576
Utaperla lepnevae	SRR13743957	25984189773	EF623003.1	circularized	1	16655	3678
Utaperla orientalis	SRR13743956	20967119021	EF623003.1	incomplete	3	15688	2372
Utaperla sopladora	SRR13743955	9705370939	EF623003.1	circularized	1	16278	429
Vandiemenella pichirichi	SRR23945927	181398988102	EU121484.1	incomplete	1	164	NA
Vandiemenella viatica	SRR23945939	243817330206	EU121484.1	circularized	1	15611	22502
Xya riparia	SRR17629532	63218113286	MK903575.1	incomplete	75	164	NA
Xyleus discoideus angulatus	SRR16892664	1571089542	OL415642.1	circularized	1	15752	146
Xyleus discoideus discoideus	SRR16892662	2033433202	OL415642.1	circularized	1	15707	520
Zapada chila	SRR17808847	1677275544	MG378125.1	incomplete	1	540	NA
