# Supplementary material for: Expanding the mitochondrial genomic toolkit for Polyneoptera: New mitogenomes and evaluation of reduced marker sets for phylogeny and DNA barcoding
Source: Genet Mol Biol. 2026 Jul 24;49(3):e20250282. doi: 10.1590/1678-4685-GMB-2025-0282 (PMC13403772; doi:10.1590/1678-4685-GMB-2025-0282)
Supplement: Table S2 - [file 1415-4757-GMB-49-3-e20250282-s2.pdf]

Genetics and Molecular Biology

DOI: https://doi.org/10.1590/1678-4685-GMB-2025-0282

Supplementary Material to "Expanding the mitochondrial genomic toolkit for Polyneoptera: New mitogenomes and evaluation of reduced marker sets for phylogeny and DNA barcoding"

Table S2 - List of mitochondrial primers identified through a literature review for each taxonomic group analyzed, including primer sequences, original sources, and documented usage in Polyneoptera.

Gene	Forward ID	Forward Sequence	Reverse ID	Reverse Sequence	Original source (Author, year)	Original source (DOI/PMID)	Polyneoptera usage (Author, year)	Polyneoptera usage (DOI/PMID)
				**Blattodea**				
16S	primer_A	CGCCTGTTTATCAAAAACAT	primer_B	CTCCGGTTTGAACTCAGATC	Xiong and Kocher, 1991	https://doi.org/10.1139/g91-050	Park et al., 2019	https://doi.org/10.1093/jee/toz097
16S	Chiar16SF	TARTYCAACACGRCGTC	Chiar16SR	CYGTRCDAAGGTAGCATA	Marquina et al., 2019	https://doi.org/10.1111/1755-0998.12942	Nagaraju et al., 2021	https://doi.org/10.1007/s42690-020-00287-5
COX1	LCO1490	GGTCAACAAATCATAAAGATATTGG	HCO2198	TAAACTTCAGGGTGACCAAAAAATCA	Folmer et al., 1994	7881515	Folmer et al., 1994	7881515
COX1	LCO1490	GGTCAACAAATCATAAAGATATTGG	HCO2198	TAAACTTCAGGGTGACCAAAAAATCA	Folmer et al., 1994	7881515	Park et al., 2019	https://doi.org/10.1093/jee/toz097
COX1	LepF1	ATTCAACCAATCATAAAGATAT	LepR1	TAAACTTCTGGATGTCCAAAAA	Hebert et al., 2004	https://doi.org/10.1073/pnas.0406166101	Schapeer et al., 2017	https://doi.org/10.1016/j.rbe.2017.02.001
COX1	AUS[F]	ATCAATTTCCATATTTGGCTT	AUS[R]	GCTGATGTAAAATAAGCTCGTG	Ma et al., 2019	https://doi.org/10.1002/ece3.5777	Ma et al., 2019	https://doi.org/10.1002/ece3.5777
COX1	H[F]	TTACCTTCGAATCTGTTATGC	H[R]	GCTGATGTAAAATAAGCTCGTG	Ma et al., 2019	https://doi.org/10.1002/ece3.5777	Ma et al., 2019	https://doi.org/10.1002/ece3.5777
COX1	Q[F]	CTCAGCCATTCTACTAACTTTGC	Q[R]	CTATAATAGGAGATGCTCTGTCTTG	Ma et al., 2019	https://doi.org/10.1002/ece3.5777	Ma et al., 2019	https://doi.org/10.1002/ece3.5777
COX1	wg550F83	ATGCGTCAGGARTGYAARTGYCAYGGYATGTC	wgcockR	AACATGCACGCACACCTCTGCACCACGGACACC	Ma et al., 2019	https://doi.org/10.1002/ece3.5777	Ma et al., 2019	https://doi.org/10.1002/ece3.5777
COX1	COI-F2	CAACAAATCATAAAGATATTGGAAC	COI-R2	TAAACTTCTGGATGACCAAAAAATCA	Yang et al., 2019	https://doi.org/10.3897/zookeys.851.31403	Luo et al., 2023	https://doi.org/10.3897/zookeys.1146.90817
COX1	COI-F3	CAACYAATCATAAAGANATTGGAAC	COI-R3	TAAACTTCAGGGTGACCAAARAATCA	Yang et al., 2019	https://doi.org/10.3897/zookeys.851.31403	Luo et al., 2023	https://doi.org/10.3897/zookeys.1146.90817

Gene	Forward ID	Forward Sequence	Reverse ID	Reverse Sequence	Original source (Author, year)	Original source (DOI/PMID)	Polyneoptera usage (Author, year)	Polyneoptera usage (DOI/PMID)
COX2	A-tLeu	CAGATAAGTGCATTGG ATTT	B-tLys	GTTTAAGAGACCAGTAC TTG	Liu and Beckenbach, 1992	https://doi.org/10.1016/1055-7903(92)90034-E	Zaman et al, 2022	https://doi.org/10.3390/f13 050674
COX2	COII-2a	ATAGAKCWTCYCCHTT AATAGAACA	COII-9b	GTACTTGCTTTCAGTCA TCTWATG	Whiting, 2002	https://doi.org/10.1046/j.0300 -3256.2001.00095.x	Park et al., 2019	https://doi.org/10.1093/jee/ toz097
COX2	COII-F-leu	TCTAATATGGCAGATT AGTGC	COII-R-lys	GAGACCAGTACTTGCTT TCAGTCATC	Whiting, 2002	https://doi.org/10.1046/j.0300 -3256.2001.00095.x	Park et al., 2019	https://doi.org/10.1093/jee/ toz097
COX2	MODA	CAGATAAGTGCATTGG ATTT	ILYSB	GTTTAAGAGACCAGTAC TTG	Ondo et al., 2023	https://doi.org/10.1111/jbi.14 671	Ondo et al., 2023	https://doi.org/10.1111/jbi. 14671
				**Phasmatodea**				
COX1	Jerry	CAACATTTATTTTGAT TTTTTGG	Tom2	ARGGGAATCATTGAATA AAWCC	Simon et al., 1994; Ribera et al., 2010	https://doi.org/10.1093/aesa/8 7.6.651; doi: 10.1186/1471-2148-10-29	Glaw et al., 2019	https://doi.org/10.3389/fev o.2019.00105
				**Plecoptera**				
16S	Inse01-F	RGACGAGAAGACCCT ATARA	Inse01-R	ACGCTGTTATCCCTAAR GTA	Taberlet et al., 2018	https://doi.org/10.1111/j.1365 -294X.2012.05542.x	Ficetola et al, 2021	https://doi.org/10.1111/me c.15632
COX1	C1-J-1718	GGAGGATTTGGAAATT GATTAGTTCC	HCO2198	TAAACTTCAGGGTGACC AAAAAATCA	Simon et al., 1994;Folmer et al., 1994	https://doi.org/10.1093/aesa/8 7.6.651; 7881515	Avelino-Capistrano et al, 2016	https://doi.org/10.11646/zo otaxa.4079.3.5
COX1	LCO1490	GGTCAACAAATCATAA AGATATTGG	HCO2198	TAAACTTCAGGGTGACC AAAAAATCA	Folmer et al., 1994	7881515	Zhou et al., 2009	https://doi.org/10.1186/17 42-9994-6-30
COX1	LCO1490	GGTCAACAAATCATAA AGATATTGG	HCO2198	TAAACTTCAGGGTGACC AAAAAATCA	Folmer et al., 1994	7881515	Elwess, 2018	https://doi.org/10.1080/00 219266.2017.1403359
COX1	LCO1490	GGTCAACAAATCATAA AGATATTGG	HCO2198	TAAACTTCAGGGTGACC AAAAAATCA	Folmer et al., 1994	7881515	Brian et al, 2014	https://doi.org/10.1086/67 4526
COX1	LCO1490	GGTCAACAAATCATAA AGATATTGG	HCO2198	TAAACTTCAGGGTGACC AAAAAATCA	Folmer et al., 1994	7881515	Maribet and Monaghan 2014	https://doi.org/10.1080/01 650424.2015.1013038
COX1	LCO1490	GGTCAACAAATCATAA AGATATTGG	HCO2198	TAAACTTCAGGGTGACC AAAAAATCA	Folmer et al., 1994	7881515	Mynott, 2015	https://doi.org/10.1071/IS1 4043
COX1	LCO1490	GGTCAACAAATCATAA AGATATTGG	HCO2198	TAAACTTCAGGGTGACC AAAAAATCA	Folmer et al., 1994	7881515	Cruz et al., 2016	https://doi.org/10.11646/zo otaxa.4193.1.4
COX1	LCO1490	GGTCAACAAATCATAA AGATATTGG	HCO2198	TAAACTTCAGGGTGACC AAAAAATCA	Folmer et al., 1994	7881515	Gattolliati et al., 2016	https://doi.org/10.11646/zo osymposia.11.1.15
COX1	LCO1490	GGTCAACAAATCATAA AGATATTGG	HCO2198	TAAACTTCAGGGTGACC AAAAAATCA	Folmer et al., 1994	7881515	Cordero et al., 2017	https://doi.org/10.1007/s00 300-016-2062-3
COX1	LCO1490	GGTCAACAAATCATAA AGATATTGG	HCO2198	TAAACTTCAGGGTGACC AAAAAATCA	Folmer et al., 1994	7881515	Morinière eta l., 2017	https://doi.org/10.1111/17 55-0998.12683

Gene	Forward ID	Forward Sequence	Reverse ID	Reverse Sequence	Original source (Author, year)	Original source (DOI/PMID)	Polyneoptera usage (Author, year)	Polyneoptera usage (DOI/PMID)
COX1	LCO1490	GGTCAACAAATCATAAAGATATTGG	HCO2198	TAAACTTCAGGGTGACCAAAAAATCA	Folmer et al., 1994	7881515	Giersch et al, 2017	https://doi.org/10.1111/gcb.13565
COX1	LCO1490	GGTCAACAAATCATAAAGATATTGG	HCO2198	TAAACTTCAGGGTGACCAAAAAATCA	Folmer et al., 1994	7881515	Teslenko et al., 2019	https://doi.org/10.11646/zootaxa.4585.3.9
COX1	LCO1490	GGTCAACAAATCATAAAGATATTGG	HCO2198	TAAACTTCAGGGTGACCAAAAAATCA	Folmer et al., 1994	7881515	Fochetti et al., 2019	https://doi.org/10.11646/zootaxa.4661.3.4
COX1	LCO1490	GGTCAACAAATCATAAAGATATTGG	HCO2198	TAAACTTCAGGGTGACCAAAAAATCA	Folmer et al., 1994	7881515	Chen et al., 2020	https://doi.org/10.11646/zootaxa.4751.2.9
COX1	LCO1490	GGTCAACAAATCATAAAGATATTGG	HCO2198	TAAACTTCAGGGTGACCAAAAAATCA	Folmer et al., 1994	7881515	Uchida et al., 2020	https://doi.org/10.7717/peerj.9176
COX1	LCO1490	GGTCAACAAATCATAAAGATATTGG	HCO2198	TAAACTTCAGGGTGACCAAAAAATCA	Folmer et al., 1994	7881515	Ge et al., 2021	https://doi.org/10.1002/ece3.7470
COX1	LCO1490	GGTCAACAAATCATAAAGATATTGG	HCO2198	TAAACTTCAGGGTGACCAAAAAATCA	Folmer et al., 1994	7881515	Hlebec et al, 2021	https://doi.org/10.3897/zookeys.1078.66382
COX1	LCO1490	GGTCAACAAATCATAAAGATATTGG	HCO2198	TAAACTTCAGGGTGACCAAAAAATCA	Folmer et al., 1994	7881515	Schröder et al., 2021	https://doi.org/10.1111/fwb.13854
COX1	LCO1490	GGTCAACAAATCATAAAGATATTGG	HCO2198	TAAACTTCAGGGTGACCAAAAAATCA	Folmer et al., 1994	7881515	Hlebec et al., 2022	https://doi.org/10.7717/peerj.13213
COX1	LCO1490	GGTCAACAAATCATAAAGATATTGG	HCO2198	TAAACTTCAGGGTGACCAAAAAATCA	Folmer et al., 1994	7881515	Vuataz et al., 2024	https://doi.org/10.1038/s41598-024-56930-5
COX1	LCO1490	GGTCAACAAATCATAAAGATATTGG	HCO2198	TAAACTTCAGGGTGACCAAAAAATCA	Folmer et al., 1994	7881515	Chen, 2024	https://doi.org/10.1016/j.jcz.2024.02.003
COX1	Jerry	CAACATTTATTTTGATTTTTTGG	PAT	TCCAATGCACTNNAATCTGCCATATTA	Simon et al., 1994	https://doi.org/10.1093/aesa/87.6.651	Mynott, 2015	https://doi.org/10.1071/IS14043
COX1	LepF	ATTCAACCAATCATAAAGATATTGG	LepR	TAAACTTCTGGATGTCCAAAAAATCA	Hebert et al., 2004	https://doi.org/10.1073/pnas.0406166101	Gill1 et al., 2014	https://doi.org/10.1086/674526
COX1	LepF	ATTCAACCAATCATAAAGATATTGG	LepR	TAAACTTCTGGATGTCCAAAAAATCA	Hebert et al., 2004	https://doi.org/10.1073/pnas.0406166101	Hlebec et al, 2021	https://doi.org/10.3897/zookeys.1078.66382
COX1	LepF	ATTCAACCAATCATAAAGATATTGG	LepR	TAAACTTCTGGATGTCCAAAAAATCA	Hebert et al., 2004	https://doi.org/10.1073/pnas.0406166101	Hlebec et al, 2021	https://doi.org/10.7717/peerj.13213
COX1	LepF	ATTCAACCAATCATAAAGATATTGG	LepR	TAAACTTCTGGATGTCCAAAAAATCA	Hebert et al., 2004	https://doi.org/10.1073/pnas.0406166101	Zieritz et al, 2022	https://doi.org/10.1111/fwb.13926
COX1	FL_rück1	TAAGCTCGGGTATCAACGTCTAT	LCO_mod	TTCTACAAATCATAAAGATATTGGAAC	Leese, 2004	https://hdl.handle.net/11858/00-001M-0000-000F-C7AF-1	Morinière et al, 2017	https://doi.org/10.1111/1755-0998.12683
COX1	LCO1490-L	GGTCWACWAATCATAAAGATATTGG	HCO2198-L	TAAACTTCWGGRTGWCCAAARAATCA	Nelson et al., 2007	https://doi.org/10.1111/j.1365-2915.2007.00664.x	Boumans and Baumann, 2012	https://doi.org/10.11646/zootaxa.3537.1.5

Gene	Forward ID	Forward Sequence	Reverse ID	Reverse Sequence	Original source (Author, year)	Original source (DOI/PMID)	Polyneoptera usage (Author, year)	Polyneoptera usage (DOI/PMID)
COX1	LCO1490-JJ	CHACWAAYCATAAAGATATYGG	HCO2198-JJ	AWACTTCVGGRTGVCCAAARAATCA	Astrin and Stüben, 2008	https://doi.org/10.1071/IS07057	Zizka et al, 2019	https://doi.org/10.1139/gen-2018-0048
COX1	BE_F	CCIGAYATRGCITTYCCICG	BE_R	GTRATIGCICCIGCIARIAC	Hajibabaei et al, 2012	https://doi.org/10.1186/1472-6785-12-28	Emilson et al, 2017	https://doi.org/10.1038/s41598-017-13157-x
COX1	FoldF	CCNGAYATRGCNTTYCCNCG	FoldR	TANACYTCNGGRTGNCCRAARAAYCA	Yu et al, 2012	https://doi.org/10.1111/j.2041-210X.2012.00198.x	Rahman et al., 2022	https://doi.org/10.1111/1365-2664.14174
COX1	mlCOIintF	GGWACWGGWTGAACWGTWTAYCCYCC	jgHCO2198	TAIACYTCIGGRTGICCRAARAAYCA	Leray et al., 2013	https://doi.org/10.1186/1742-9994-10-34	Zieritz et al, 2022	https://doi.org/10.1111/fwb.13926
COX1	mlCOIintF	GGWACWGGWTGAACWGTWTAYCCYCC	HCO2198	TAAACTTCAGGGTGACCAAAAAATCA	Leray et al., 2013;Folmer et al., 1994	https://doi.org/10.1186/1742-9994-10-34; 7881515	Keck et al, 2022	https://doi.org/10.3897/mbmg.6.79351
COX1	LepF	ATTCAACCAATCATAAAGATATTGG	LepR	TAAACTTCTGGATGTCCAAAAAATCA	Hebert et al., 2004	https://doi.org/10.1073/pnas.0406166101	Morinière et al, 2017	https://doi.org/10.1111/1755-0998.12683
COX1	mod_LCO1490	TYTCAACAAATCAYAARGAYATTGG	mod_HCO2198	TAYACYTCWGGRTGMCCAAAAAATCA	Giersch et al., 2015	https://doi.org/10.1086/679490	Giersch et al., 2015	https://doi.org/10.1086/679490
COX1	fwhF2	GGDACWGGWTGAACWGTWTAYCCHCC	fwhR2n	GTRATWGCHCCDGCTARWACWGG	Elbrecht et al., 2017	https://doi.org/10.1111/2041-210X.12789	Remmel et al, 2024	https://doi.org/10.1111/icad.12710
COX1	BF2	GCHCCHGAYATRGCHTTYCC	BR2	TCDGGRTGNCCRAAARAAYCA	Elbrecht and Leese, 2017	https://doi.org/10.3389/fenvs.2017.00011	Zizka et al, 2020	https://doi.org/10.1016/j.ecolind.2020.106383
COX1	BF2	GCHCCHGAYATRGCHTTYCC	BR2	TCDGGRTGNCCRAAARAAYCA	Elbrecht and Leese, 2017	https://doi.org/10.3389/fenvs.2017.00011	Beermann et al, 2020	https://doi.org/10.1016/j.scitotenv.2020.141969
COX1	BF2	GCHCCHGAYATRGCHTTYCC	BR2	TCDGGRTGNCCRAAARAAYCA	Elbrecht and Leese, 2017	https://doi.org/10.3389/fenvs.2017.00011	Ficetola et al, 2021	https://doi.org/10.1111/mec.15632
COX1	BF2	GCHCCHGAYATRGCHTTYCC	BR2	TCDGGRTGNCCRAAARAAYCA	Elbrecht and Leese, 2017	https://doi.org/10.3389/fenvs.2017.00011	Rivera et al, 2021	https://doi.org/10.1016/j.scitotenv.2020.144208
COX1	BF2	GCHCCHGAYATRGCHTTYCC	BR2	TCDGGRTGNCCRAAARAAYCA	Elbrecht and Leese, 2017	https://doi.org/10.3389/fenvs.2017.00011	Turunen, et al, 2021	https://doi.org/10.1111/fwb.13678
				**Orthoptera**				
12S	12Sai	AAACTAGGATTAGATACCCTATTAT	12Sbi	AAGAGCGACGGGCGATGTGT	Simon et al., 1994	https://doi.org/10.1093/aesa/87.6.651	Allegrucci et al, 2021	https://doi.org/10.1080/24750263.2021.1902005
12S	12Sai	AAACTAGGATTAGATACCCTATTAT	12Sbi	AAGAGCGACGGGCGATGTGT	Simon et al., 1994	https://doi.org/10.1093/aesa/87.6.651	Allegrucci et al, 2014	https://doi.org/10.3897/zookeys.437.7917
16S	16Sar	CGCCTGTTTAACAAAAACAT	16Sbr	CCGGTCTGAACTCAGATCACGT	Simon et al., 1994	https://doi.org/10.1093/aesa/87.6.651	Allegrucci et al, 2014	https://doi.org/10.3897/zookeys.437.7917
16S	16Sar	CGCCTGTTTAACAAAAACAT	16Sbr	CCGGTCTGAACTCAGATCACGT	Simon et al., 1994	https://doi.org/10.1093/aesa/87.6.651	Allegrucci et al, 2021	https://doi.org/10.1080/24750263.2021.1902005

Gene	Forward ID	Forward Sequence	Reverse ID	Reverse Sequence	Original source (Author, year)	Original source (DOI/PMID)	Polyneoptera usage (Author, year)	Polyneoptera usage (DOI/PMID)
16S	LR-J-13417	ATGTTTTTGTTAAACAGGCG	SR-N-14588	AAACTAGGATTAGATACCCTATTAT	Simon et al., 1994	https://doi.org/10.1093/aesa/87.6.651	Trewick, 2008	https://doi.org/10.1111/j.1096-0031.2007.00174.x
16S	LR-J-13417	ATGTTTTTGATAAACAGGCG	SR-N-14275	AAGGTGGATTTGATAGTAAT	Simon et al., 1994	https://doi.org/10.1093/aesa/87.6.651	Yassin et al, 2009	https://doi.org/10.1016/j.ympev.2009.06.012
16S	16Sb	CCGGTCTGAACTCAGATCACGT	16Sar	CGCCTGTTTATCAAAAACAT	Simon et al., 1994	https://doi.org/10.1093/aesa/87.6.651	Iorgu et al, 2023	https://doi.org/10.1093/zoolinnean/zlab084
COX1	C1-J-1859	GGAACnGGATGAACAGTAT	C1-N-2329	ACTGTAAATATATGATGAGCTCA	Simon et al., 1994	https://doi.org/10.1093/aesa/87.6.651	Bonelli et al, 2019	http://www.bulletinofinsectology.org/pdfarticles/vol72-2019-103-114bonelli.pdf
COX1	C1-N-2195	TTGATTTTTTGGTCATCCAGAAGT	C1-J-3014	TCCAATGCACTAATCTGCCATATTA	Simon et al., 1994	https://doi.org/10.1093/aesa/87.6.651	Trewick, 2008	https://doi.org/10.1111/j.1096-0031.2007.00174.x
COX1	LCO1490 (Folmer J)	GGTCACAAATCATAAAGATATTGG	HCO2198 (Folmer N)	TAAACTTCAGGGTGACCAAAAAATCA	Folmer et al., 1994	7881515	Moulton et al, 2010	https://doi.org/10.1111/j.1755-0998.2009.02823.x
COX1	LCO1490	GGTCAACAAATCATAAAGATATTGG	HCO2198	TAAACTTCAGGGTGACCAAAAAATCA	Folmer et al., 1994	7881515	Zhou et al, 2013	https://doi.org/10.1071/IS12019
COX1	LCO1490	GGTCAACAAATCATAAAGATATTGG	HCO2198	TAAACTTCAGGGTGACCAAAAAATCA	Folmer et al., 1994	7881515	Allegrucci et al, 2014	https://doi.org/10.3897/zookeys.437.7917
COX1	LCO1490	GGTCAACAAATCATAAAGATATTGG	HCO2198	TAAACTTCAGGGTGACCAAAAAATCA	Folmer et al., 1994	7881515	Pedraza-Lara et al, 2015	https://doi.org/10.1016/j.ympev.2015.01.001
COX1	LCO1490	GGTCAACAAATCATAAAGATATTGG	HCO2198	TAAACTTCAGGGTGACCAAAAAATCA	Folmer et al., 1994	7881515	Vitale et al, 2015	https://doi.org/10.1016/j.micron.2015.08.002
COX1	LCO1490	GGTCAACAAATCATAAAGATATTGG	HCO2198	TAAACTTCAGGGTGACCAAAAAATCA	Folmer et al., 1994	7881515	Kang et al, 2016	https://doi.org/10.3109/19401736.2015.1022730
COX1	LCO1490	GGTCAACAAATCATAAAGATATTGG	HCO2198	TAAACTTCAGGGTGACCAAAAAATCA	Folmer et al., 1994	7881515	De Jesús-Bonilla et al, 2017	https://doi.org/10.1080/14772000.2017.1313792
COX1	LCO1490	GGTCAACAAATCATAAAGATATTGG	HCO2198	TAAACTTCAGGGTGACCAAAAAATCA	Folmer et al., 1994	7881515	Ortega-Morales et al, 2017	https://doi.org/10.11646/zootaxa.4258.2.9
COX1	LCO1490	GGTCAACAAATCATAAAGATATTGG	HCO2198	TAAACTTCAGGGTGACCAAAAAATCA	Folmer et al., 1994	7881515	Zhou et al, 2019	https://doi.org/10.1186/s12862-019-1404-5
COX1	LCO1490	GGTCAACAAATCATAAAGATATTGG	HCO2198	TAAACTTCAGGGTGACCAAAAAATCA	Folmer et al., 1994	7881515	Kim et al, 2020	https://doi.org/10.1111/1748-5967.12433
COX1	LCO1490	GGTCAACAAATCATAAAGATATTGG	HCO2198	TAAACTTCAGGGTGACCAAAAAATCA	Folmer et al., 1994	7881515	Ahmad et al, 2020	https://dx.doi.org/10.17582/journal.pjz/20200501020521

Gene	Forward ID	Forward Sequence	Reverse ID	Reverse Sequence	Original source (Author, year)	Original source (DOI/PMID)	Polyneoptera usage (Author, year)	Polyneoptera usage (DOI/PMID)
COX1	LCO1490	GGTCAACAAATCATAAAGATATTGG	HCO2198	TAAACTTCAGGGTGACCAAAAAATCA	Folmer et al., 1994	7881515	Allegrucci et al, 2021	https://doi.org/10.1080/24750263.2021.1902005
COX1	LCO1490	GGTCAACAAATCATAAAGATATTGG	HCO2198	TAAACTTCAGGGTGACCAAAAAATCA	Folmer et al., 1994	7881515	Tlil et al, 2021	https://doi.org/10.5252/zoosystema2020v42a31
COX1	LCO1490	GGTCAACAAATCATAAAGATATTGG	HCO2198	TAAACTTCAGGGTGACCAAAAAATCA	Folmer et al., 1994	7881515	Warchalowska-Sliwa et al, 2021	https://doi.org/10.1038/s41598-021-02110-8
COX1	LCO1490	GGTCAACAAATCATAAAGATATTGG	HCO2198	TAAACTTCAGGGTGACCAAAAAATCA	Folmer et al., 1994	7881515	Kang et al, 2022	https://doi.org/10.1111/1748-5967.12619
COX1	LCO1490	GGTCAACAAATCATAAAGATATTGG	HCO2198	TAAACTTCAGGGTGACCAAAAAATCA	Folmer et al., 1994	7881515	Iorgu et al, 2023	https://doi.org/10.1093/zoolinnean/zlab084
COX1	LCO1490	GGTCAACAAATCATAAAGATATTGG	HCO2198	TAAACTTCAGGGTGACCAAAAAATCA	Folmer et al., 1994	7881515	Guerrero and Cayabyab, 2024	https://doi.org/10.18474/JES23-36
COX1	LCO1490	GGTCAACAAATCATAAAGATATTGG	Ill_C_R	GGIGGRTAIACIGTTCAICC	Folmer et al., 1994;Shokralla et al., 2015	7881515; https://doi.org/10.1038/srep09687	Pina et al, 2024	https://doi.org/10.3897/BDJ.12.e118010
COX1	LCO1490	TCWACWAAYCAYAARGAYATYGG	COmfd_R	GGDGGRTANAHHGTTCAHCCNGTHCC	Folmer et al., 1994; Kurata et al , 2024	7881515; https://doi.org/10.3897/bdj.12.e117014	Kurata et al , 2024	https://doi.org/10.3897/bdj.12.e117014
COX1	COmfd_F	CCNCGRHTRAAYAAYATRAGWTTYTG	HCO2198	ACTTCDGGRTGNCCAAARAAYCA	Kurata et al , 2024; Folmer et al., 1994	https://doi.org/10.3897/bdj.12.e117014; 7881515	Kurata et al , 2024	https://doi.org/10.3897/bdj.12.e117014
COX1	Ill_B_F	CCIGAYATRGCITTYCCICG	HCO2198	TAAACTTCAGGGTGACCAAAAAATCA	Shokralla et al., 2015;Folmer et al., 1994	https://doi.org/10.1038/srep09687; 7881515	Pina et al, 2024	https://doi.org/10.3897/BDJ.12.e118010
COX1	UEA1	GAATAATTCCCATAAATAGATTTACA	UEA10	TCCAATGCACTAATCTGCCATATTA	Lunt et al., 1996	https://doi.org/10.1111/j.1365-2583.1996.tb00049.x	Allegrucci et al, 2014	https://doi.org/10.3897/zookeys.437.7917
COX1	UEA5	AGTTTTAGCAGGAGCAATTACTAT	UEA10	TCCAATGCACTAATCTGCCATATTA	Lunt et al., 1996	https://doi.org/10.1111/j.1365-2583.1996.tb00049.x	Allegrucci et al, 2014	https://doi.org/10.3897/zookeys.437.7917
COX1	ACOIAF	CWAATCAYAAAGATATTGGAAC	ACOIAR	AATATAWACTTCWGGGTGACC	Colgan et al., 2001	https://doi.org/10.1017/S002531540100457X	Iorgu et al, 2023	https://doi.org/10.1093/zoolinnean/zlab084
COX1	MhemF	GCATTYCCACGAATAAATAAYATAAG	dgHCO2198	TAAACTTCAGGGTGACCAAARAAYCA	Park, D. S., 2011;Meyer, C. P., 2003	https://doi.org/10.1371/journal.pone.0018749; https://doi.org/10.1046/j.1095-8312.2003.00197.x	Beng et al, 2016	https://doi.org/10.1038/srep24965
COX1	LepF1	ATTCAACCAATCATAAAGATATTGG	LepR1	TAAACTTCTGGATGTCCAAAAAATCA	Hebert et al., 2004	https://doi.org/10.1073/pnas.0406166101	Ahmed et al, 2024	https://doi.org/10.35495/ajab.2023.333

Gene	Forward ID	Forward Sequence	Reverse ID	Reverse Sequence	Original source (Author, year)	Original source (DOI/PMID)	Polyneoptera usage (Author, year)	Polyneoptera usage (DOI/PMID)
COX1	COBU	TYTCAACAAAYCAYAARGATATTGG	COBL	TAAACTTCWGGRTGWCCAAARAATCA	Pan et al. 2006	Entomotaxonomia. 28:103–110	Huang et al, 2013	https://doi.org/10.1371/journal.pone.0082400
COX1	COBU	TYTCAACAAAYCAYAARGATATTGG	COBL	TAAACTTCWGGRTGWCCAAARAATCA	Pan et al. 2006	Entomotaxonomia. 28:103–110	Guo et al, 2016	https://doi.org/10.3897/zookeys.596.8669
COX1	COBU	TYTCAACAAAYCAYAARGATATTGG	COBL	TAAACTTCWGGRTGWCCAAARAATCA	Pan et al. 2006	Entomotaxonomia. 28:103–110	Zhao et al, 2016	https://doi.org/10.3897/zookeys.582.6301
COX1	COBU	TYTCAACAAAYCAYAARGATATTGG	COBL	TAAACTTCWGGRTGWCCAAARAATCA	Pan et al. 2006	Entomotaxonomia. 28:103–110	Hawlitschek et al, 2017	https://doi.org/10.1111/1755-0998.12638
COX1	COBU	TYTCAACAAAYCAYAARGATATTGG	COBL	TAAACTTCWGGRTGWCCAAARAATCA	Pan et al. 2006	Entomotaxonomia. 28:103–110	Kundu et al, 2020	https://doi.org/10.1080%2F23802359.2020.1830725
COX1	COBU	TYTCAACAAAYCAYAARGATATTGG	COBL	TAAACTTCWGGRTGWCCAAARAATCA	Pan et al. 2006	Entomotaxonomia. 28:103–110	Hafayed et al, 2023	https://doi.org/10.1080/15627020.2023.2263498
COX1	COBU	TYTCAACAAAYCAYAARGATATTGG	COBL	TAAACTTCWGGRTGWCCAAARAATCA	Pan et al. 2006	Entomotaxonomia. 28:103–110	Zheng et al, 2023	https://doi.org/10.3897/asp.81.e104772
COX1	COBU	TYTCAACAAAYCAYAARGATATTGG	COBL	TAAACTTCWGGRTGWCCAAARAATCA	Pan et al. 2006	Entomotaxonomia. 28:103–110	Hou et al, 2024	https://doi.org/10.3897/zookeys.1193.117612
COX1	COBU	TYTCAACAAAYCAYAARGATATTGG	COBL	TAAACTTCWGGRTGWCCAAARAATCA	Pan et al. 2006	Entomotaxonomia. 28:103–110	Kim et al, 2024	https://doi.org/10.1016/j.aspen.2024.102249
COX1	COBU	TYTCAACAAAYCAYAARGATATTGG	COBL	TAAACTTCWGGRTGWCCAAARAATCA	Pan et al. 2006	Entomotaxonomia. 28:103–110	Kock et al, 2024	https://doi.org/10.3390/insects15020128
COX1	COBU	TYTCAACAAAYCAYAARGATATTGG	COBL	TAAACTTCWGGRTGWCCAAARAATCA	Pan et al. 2006	Entomotaxonomia. 28:103–110	Zhang et al, 2024	https://doi.org/10.1002/arch.22070
COX1	Teleogryllus_1F	CTATCGCCTATTATTCAGCCACT	Teleogryllus_1R	GGGGAGATGATCTATTTTGTAGA	Ye et al., 2008	https://doi.org/10.3724/SP.J.1141.2008.03236	Kim et al, 2022	https://doi.org/10.1016/j.aspen.2022.101959
COX1	Teleogryllus_2F	CACTTTATCGCAACGATGATTAT	Teleogryllus_2R	GCACTTTTCTGCCATATTAGTAG	Ye et al., 2008	https://doi.org/10.3724/SP.J.1141.2008.03236	Kim et al, 2022	https://doi.org/10.1016/j.aspen.2022.101959
COX1	Anabrus J	GGTTACTACAAATAAAGTGGTATTGG	Anabrus N	TAAACTGCAGGGTGACCAAAAAATCA	Moulton et al, 2010	https://doi.org/10.1111/j.1755-0998.2009.02823.x	Moulton et al, 2010	https://doi.org/10.1111/j.1755-0998.2009.02823.x
COX1	Locusta J	GGTCACCAGCTATAAAGACATTGG	Locusta N	TAAACTTCAGGGTGACCGAAAAATGA	Moulton et al, 2010	https://doi.org/10.1111/j.1755-0998.2009.02823.x	Moulton et al, 2010	https://doi.org/10.1111/j.1755-0998.2009.02823.x
COX1	Myrmecophilus J	GGTCACAAATCATAAAGATATCAGG	Myrmecophilus N	TAAATTTCAGGGTGACTAAAAAATCA	Moulton et al, 2010	https://doi.org/10.1111/j.1755-0998.2009.02823.x	Moulton et al, 2010	https://doi.org/10.1111/j.1755-0998.2009.02823.x
COX1	Schistocerca J	GGTCACAAATCCGCAAAGATATTGG	Schistocerca N	TAAACTTCAGGGTGACCAAAAAATGA	Moulton et al, 2010	https://doi.org/10.1111/j.1755-0998.2009.02823.x	Moulton et al, 2010	https://doi.org/10.1111/j.1755-0998.2009.02823.x
COX1	Orthoptera-specific J	GGTCACAAATCATAAAGCGTATTGG	Orthoptera-specific N	TAAACTTCAGGGTGACCGGAAAATCA	Moulton et al, 2010	https://doi.org/10.1111/j.1755-0998.2009.02823.x	Moulton et al, 2010	https://doi.org/10.1111/j.1755-0998.2009.02823.x

Gene	Forward ID	Forward Sequence	Reverse ID	Reverse Sequence	Original source (Author, year)	Original source (DOI/PMID)	Polyneoptera usage (Author, year)	Polyneoptera usage (DOI/PMID)
COX1	Orthoptera-specific J	GGTCACAAATCATAAAGCGTATTGG	Orthoptera-specific N	TAAACTTCAGGGTGACCGGAAAATCA	Moulton et al, 2010	https://doi.org/10.1111/j.1755-0998.2009.02823.x	Gutiérrez-Rodríguez et al, 2022	https://doi.org/10.1071/IS21022
COX1	COI-F	CCATCTTACCGCAAAAATGAT	COI-R	CTGGGTGWCCAAAGAATCAAA	Husemann et al. , 2012	http://dx.doi.org/10.1111/j.1463-6409.2012.00548.x	Moussi et al, 2018	https://doi.org/10.1080/15627020.2018.1463172
COX1	COI-F	CCATCTTACCGCAAAAATGAT	COI-R	CTGGGTGWCCAAAGAATCAAA	Husemann et al. , 2012	http://dx.doi.org/10.1111/j.1463-6409.2012.00548.x	Sofrane et al, 2022	https://www.redalyc.org/journal/3220/322070162008/322070162008.pdf
COX1	mICOIintF	GGWACWGGWTGAACWGTWTAYCCYCC	jgHCO2198	TANACYTCNGGRTGNCCRAARAAYCA	Leray et al., 2013	https://doi.org/10.1186/1742-9994-10-34	Li et al, 2023	https://doi.org/10.1002/ece3.10031
COX1	MDG-F	TYTCAACWAAYCAYAARGAYATYGG	MDG-R	TADACTTCWGGRTGWCCRAARAATCA	Sofrane et al, 2015	https://doi.org/10.1080/00379271.2015.1054647	Sofrane et al, 2015	https://doi.org/10.1080/00379271.2015.1054647
COX1	OTLCOF	TCAACAAACCATAAGGACATTGG	OTHCOR	ATATGTGAAATAATACCAAATCCTGG	Kang et al, 2016	https://doi.org/10.3109/19401736.2015.1022730	Kang et al, 2016	https://doi.org/10.3109/19401736.2015.1022730
COX1	MDG-F	TYTCAACWAAYCAYAARGAYATYGG	MDG-R	TADACTTCWGGRTGWCCRAARAATCA	Sofrane et al, 2022	https://www.redalyc.org/journal/3220/322070162008/322070162008.pdf	Sofrane et al, 2022	https://www.redalyc.org/journal/3220/322070162008/322070162008.pdf
COX1	COX1-F	CTCRACRAATCATAAAGATATYGG	COX1-R2	GTRTCWACATCTATWCCTACAG	Suastes-Jiménez et al, 2023	https://doi.org/10.22201/ib.20078706e.2023.94.5184	Suastes-Jiménez et al, 2023	https://doi.org/10.22201/ib.20078706e.2023.94.5184
COX3	COX3_fwd	CCTTGACCATTAACAGGAGCAATTGGA	COX3_rev	TGTCAGTATCATGCTGCTGCTTCAA	Pereira et al, 2021	https://doi.org/10.1111/jzs.12446	Pereira et al, 2021	https://doi.org/10.1111/jzs.12446
CYTB	CB-J10933	GTTTTACCATGAGGTCAAATATC	CB-N11526	TTCTACTGGTCGRGCTCCAATTCA	Simon et al., 1994	https://doi.org/10.1093/aesa/87.6.651	Zhou et al, 2013	https://doi.org/10.1071/IS12019
CYTB	CB1	TATGTACTACCATGAGGACAAATATC	CB2	ATTACACCTCCTAATTTATTAGGAAT	Jermiin & Crozier, 1994	https://doi.org/10.1007/bf00176090	Pedraza-Lara et al, 2015	https://doi.org/10.1016/j.ympev.2015.01.001
CYTB	CB1	TATGTACTACCATGAGGACAAATATC	CB2	ATTACACCTCCTAATTTATTAGGAAT	Jermiin & Crozier, 1994	https://doi.org/10.1007/bf00176090	De Jesús-Bonilla et al, 2017	https://doi.org/10.1080/14772000.2017.1313792
CYTB	CYTB_fwd	CGAACACTACACGCAAATGGAGCA	CYTB_rev	AGGTTCTTCAACTGGTCGTTTTCCA	Pereira et al, 2021	https://doi.org/10.1111/jzs.12446	Pereira et al, 2021	https://doi.org/10.1111/jzs.12446
ND2	ND2A	CGTTGATGATAGGAACGTACC	ND2B	GGTGTCTAATTGATGATTATGC	Tokuda et al., 2010	https://doi.org/10.1111/j.1095-8312.2010.01386.x	Tlili et al, 2021	https://doi.org/10.5252/zoosystema2020v42a31
				**Mantodea**				
COX1	LCO1490	GGTCAACAAATCATAAAGATATTGG	HCO2198	TAAACTTCAGGGTGACCAAAAAATCA	Folmer et al., 1994	7881515	Govorov t al, 2024	https://doi.org/10.1371/journal.pone.0304163

Gene	Forward ID	Forward Sequence	Reverse ID	Reverse Sequence	Original source (Author, year)	Original source (DOI/PMID)	Polyneoptera usage (Author, year)	Polyneoptera usage (DOI/PMID)
COX1	LCO1490	GGTCAACAAATCATAA AGATATTGG	HCO2198	TAAACTTCAGGGTGACC AAAAAATCA	Folmer et al., 1994	7881515	Battiston et al, 2014	https://doi.org/10.11646/zootaxa.3797.1.8
COX1	LCO1490	GGTCAACAAATCATAA AGATATTGG	HCO2198	TAAACTTCAGGGTGACC AAAAAATCA	Folmer et al., 1994	7881515	Scherrer, 2014	https://doi.org/10.11646/zootaxa.3797.1.15
COX1	LCO1490	GGTCAACAAATCATAA AGATATTGG	HCO2198	TAAACTTCAGGGTGACC AAAAAATCA	Folmer et al., 1994	7881515	Porter et al, 2014	https://doi.org/10.1111%2F1755-0998.12240
COX1	LCO1490	GGTCAACAAATCATAA AGATATTGG	HCO2198	TAAACTTCAGGGTGACC AAAAAATCA	Folmer et al., 1994	7881515	Maioglio et al, 2023	https://doi.org/10.1080/00379271.2023.2171487
COX1	LCO1490	GGTCAACAAATCATAA AGATATTGG	Nancy-R	CCCGGTAAAATTAAAAT ATAAACTTC	Folmer et al., 1994; Simon et al., 1994	7881515; https://doi.org/10.1093/aesa/87.6.651	de Alcantara Viana, et al, 2023	https://doi.org/10.1038/s41598-023-46204-x
COX1	C_LepFolF	ATTCAACCAATCATAA AGATAT	C_LepFolR	TAAACTTCTGGATGTCC AAAAA	Hebert et al., 2004	https://doi.org/10.1073/pnas.0406166101	Moulin , 2020	https://doi.org/10.3897/zookeys.917.39270
COX1	CO1–C02	AYTCAACAAATCATAA AGATATTGG	CO1–C04	ACYTCRGGRTGACCAAA AAATCA	Che et al., 2012	https://doi.org/10.1111/j.1755-0998.2011.03090.x	Song et al, 2020	https://doi.org/10.1016/j.jep.2020.112574
COX1	COH6	TADACTTCDGGRTGDC CAAARAAYCA	COL6b	ACAAATCATAAAGATAT YGG	Mantelatto et al, 2016	https://doi.org/10.1590/2358-2936e2016030	Govorov et al, 2024	https://doi.org/10.1371/journal.pone.0304163

* The colors indicate that the same primer sets were used across different studies.
