# Supplementary material for: Expanding the mitochondrial genomic toolkit for Polyneoptera: New mitogenomes and evaluation of reduced marker sets for phylogeny and DNA barcoding
Source: Genet Mol Biol. 2026 Jul 24;49(3):e20250282. doi: 10.1590/1678-4685-GMB-2025-0282 (PMC13403772; doi:10.1590/1678-4685-GMB-2025-0282)
Supplement: Table S3 - [file 1415-4757-GMB-49-3-e20250282-s3.pdf]

Genetics and Molecular Biology

DOI: https://doi.org/10.1590/1678-4685-GMB-2025-0282

Supplementary Material to "Expanding the mitochondrial genomic toolkit for Polyneoptera: New mitogenomes and evaluation of reduced marker sets for phylogeny and DNA barcoding"

Table S3 - Accession number of the sequences used for the barcoding gap analysis.

Species	Gene											
	12S	***16S***	***ATP6***	***ATP8***	***COX1***	***COX2***	***CYTB***	***ND2***	***ND4***	***ND4L***	***ND5***	***ND6***
**Blattodea**												
Allacta bimaculata	NA	NA	NA	NA	PP133874, PP133873, PP133870, PP133869, MG882128	NA	NA	NA	NA	NA	NA	NA
Anaplecta omei	NA	NA	NA	NA	OL790058, OL790057, OL790056, MT800290, MT800289, MT800288, MT800287, MT800286, MT800285, MG882129	NA	NA	NA	NA	NA	NA	NA
Anoplotermes banksi	NA	NA	NA	NA	NA	OQ980233, OL875045, MH068012, MH068011, KY224509	NA	NA	NA	NA	NA	NA
Blattella germanica	NA	NA	NA	NA	PP854577, PP854576, PP854575, PP758930, PP754525, PP754240, PP754236, PP754234,	PP101131, PP101130, PP101129, PP101128, PP101127, PP101126, PP101125, PP101124,	NA	NA	NA	NA	NA	NA

Species	Gene											
	12S	***16S***	***ATP6***	***ATP8***	***COX1***	***COX2***	***CYTB***	***ND2***	***ND4***	***ND4L***	***ND5***	***ND6***
					PP754233, PP754231	PP101123, PP101122						
Bundoksia longissima	NA	NA	NA	NA	OM370893, OM370892, OM370891, OM370890, OM370889, OM370888, OM370887, OM370886, OM370885, OM370884	NA	NA	NA	NA	NA	NA	NA
Capritermes capricornis	NA	NA	NA	NA	NA	OQ645340, OQ555372, FJ818900, FJ818899	NA	NA	NA	NA	NA	NA
Cavitermes tuberosus	NA	NA	OQ998960, OQ451185, OQ440400, OL875046, KY224568, KP026294	NA	OQ998960, OQ451185, OQ440400, OL875046, KY224568, KP026294	OQ998960, OQ451185, OQ440400, OL875046, MH067996, MH067995, MH067994, KY224568, KP026294	NA	NA	NA	NA	OQ998960, OQ451185, OQ440400, OL875046	OQ998960, OQ451185, OQ440400, OL875046
Coptotermes acinaciformis	NA	NA	KU925199, KU925198, KU925197, KU925196	NA	MZ424795, KU925199, KU925198, KU925197, KU925196, KJ918347, KJ918344, KJ918341, KJ918340, KJ918339	KU925199, KU925198, KU925197, KU925196, KJ918347, KJ918344, KJ918341, KJ918340, KJ918339, KJ918338	NA	NA	NA	NA	NA	NA
Coptotermes formosanus	NA	NA	OR069384, KU925203, AB626147, AB626146, AB626145	NA	OR069384, MZ424477, MZ424471, MZ424470, MZ424469, MZ424468, MZ424467, MZ424466, MZ420500, MZ413511	OR069384, MW558361, MW558360, MW558359, MW558358, MW558364, MW558363, MW558362, MF410753, MF410752	NA	NA	NA	NA	OR069384, AB626147, AB626146, AB626145	OR069384, AB626147, AB626146, AB626145

Species	Gene											
	12S	***16S***	***ATP6***	***ATP8***	***COX1***	***COX2***	***CYTB***	***ND2***	***ND4***	***ND4L***	***ND5***	***ND6***
Coptotermes frenchi	NA	NA	NA	NA	KU925204, KJ918345, KJ918343, KJ918342, KJ918322, KJ918320, KJ918302, KJ918301, KJ918251	KU925204, KJ918345, KJ918343, KJ918342, KJ918322, KJ918320, KJ918302, KJ918301, KJ918251	NA	NA	NA	NA	NA	NA
Coptotermes gestroi	NA	NA	NA	NA	OP422957, OP422956, OP422955, OP422954, OP422953, OP422952, OL881264, OL881263, OL881262, OL881261	NA	NA	NA	NA	NA	NA	NA
Coptotermes heimi	NA	NA	NA	NA	ON529969, ON529968, ON529967, ON529966, ON529965, ON529964, ON529963, ON529962, ON529961, ON529960	NA	NA	NA	NA	NA	NA	NA
Coptotermes lacteus	NA	NA	NA	NA	KU925211, KJ918299, KJ918296, KJ918294, KJ918293, KJ918292, KJ918259, KJ918258, KJ918257, JX144934	KU925211, KJ918299, KJ918296, KJ918294, KJ918293, KJ918292, KJ918259, KJ918258, KJ918257, JX144934	NA	NA	NA	NA	NA	NA
Coptotermes michaelseni	NA	NA	NA	NA	KU925212, KJ918349, KJ918348, KJ918346, KJ918326, KJ918316	KU925212, KJ918349, KJ918348, KJ918346, KJ918326, KJ918316	NA	NA	NA	NA	NA	NA

**Species**	**Gene**											
	12S	***16S***	***ATP6***	***ATP8***	***COX1***	***COX2***	***CYTB***	***ND2***	***ND4***	***ND4L***	***ND5***	***ND6***
Coptotermes testaceus	NA	NA	OQ446319, OQ434259, OL875050, KU925220, KU925219, KU925218, KR872938	NA	OQ446319, OQ434259, OL875050, MZ424789, MZ424476, KU925220, KU925219, KU925218, KR872938	OQ446319, OQ434259, OL875050, KU925220, KU925219, KU925218, KR872938	NA	NA	NA	NA	OQ446319, OQ434259, OL875050, KR872938	OQ446319, OQ434259, OL875050, KR872938
Coptotermes truncatus	NA	NA	NA	NA	OQ675031, OQ675030, KJ918304, KJ918303	OQ675031, OQ675030, FJ818989, FJ818988, FJ818987, FJ818986, KJ918304, KJ918303	NA	NA	NA	NA	NA	NA
Cortaritermes intermedius	NA	NA	NA	NA	NA	MH577236, MN803319, MN803317, MH067987	NA	NA	NA	NA	NA	NA
Cryptotermes brevis	NA	NA	NA	NA	OM991333, OK506051, MK618724, MT040337	OM991333, OK506051, MK618724, LC527384	NA	NA	NA	NA	NA	NA
Cryptotermes cavifrons	NA	NA	OM991429, OM991349, OM991310, OL875054	NA	OM991429, OM991349, OM991310, OL875054	OM991429, OM991349, OM991310, OL875054	NA	NA	NA	NA	OM991429, OM991349, OM991310, OL875054	OM991429, OM991349, OM991310, OL875054
Cryptotermes declivis	NA	NA	NA	NA	NA	LC527401, LC527400, LC527399, LC527398, LC527397, MK599465	NA	NA	NA	NA	NA	NA
Cryptotermes domesticus	NA	NA	NA	NA	OM991399, OM991334, MT010558, LC524000, LC523999, LC523998, LC523997, LC523996, LC523995, LC523994	OM991399, OM991334, MT010558, LC527396, LC527395, LC527394, LC527393, LC527392, LC527391, LC527390	NA	NA	NA	NA	NA	NA

Species	Gene											
	12S	***16S***	***ATP6***	***ATP8***	***COX1***	***COX2***	***CYTB***	***ND2***	***ND4***	***ND4L***	***ND5***	***ND6***
Cryptotermes havilandi	NA	NA	OM991433, OM991385, OM991316, OM991313, OL469805, MW208858	NA	OQ852745, OQ852744, OM991433, OM991385, OM991316, OM991313, OL469805, MW208858	OM991433, OM991385, OM991316, OM991313, OL469805, MW208858	NA	NA	NA	NA	OM991433, OM991385, OM991316, OM991313, MW208858	OM991433, OM991385, OM991316, OM991313, MW208858
Dolichorhinotermes longilabius	NA	NA	OQ980237, OQ446313, OL875051, KP026258	NA	OQ980237, OQ446313, OL875051, KP026258	OQ980237, OQ446313, OL875051, KP026258	NA	NA	NA	NA	NA	NA
Embiratermes neotenicus	NA	NA	OL875044, KY436202, KY436201, KP026262	NA	OR946202, OR946201, OR946200, OR946199, OR946198, OR946197, OR946196, OR946195, OR946194, OR946193	OL875044, MH068003, MH068002, MH068001, KY436202, KY436201, KP026262	NA	NA	NA	NA	NA	NA
Engelitermes zambo	NA	NA	OP882290, OP882289, OP882288, OP882287	NA	OP882290, OP882289, OP882288, OP882287	OP882290, OP882289, OP882288, OP882287	NA	NA	NA	NA	OP882290, OP882289, OP882288, OP882287	OP882290, OP882289, OP882288, OP882287
Eupolyphaga sinensis	NA	NA	NA	NA	OP215847, OP215846, OP215845, MT593331, MG882184	NA	NA	NA	NA	NA	NA	NA
Geoscapheus dilatatus	NA	NA	MW601004, MW601003, MW601002, MW601001, MW601000, MW600999, MW600998, MW600997, MW600996, MW600995	NA	MW601004, MW601003, MW601002, MW601001, MW601000, MW600999, MW600998, MW600997, MW600996, MW600995	MW601004, MW601003, MW601002, MW601001, MW601000, MW600999, MW600998, MW600997, MW600996, MW600995	NA	NA	NA	NA	NA	NA
Heterotermes convexinotatus	NA	NA	NA	NA	NA	OQ440399, MN313721, MN313720, MN313719,	NA	NA	NA	NA	NA	NA

Species	Gene											
	12S	***16S***	***ATP6***	***ATP8***	***COX1***	***COX2***	***CYTB***	***ND2***	***ND4***	***ND4L***	***ND5***	***ND6***
						MN313718, MN313717, MN313716, MN313715, MN313714, MN313713						
Heterotermes tenuis	NA	NA	NA	NA	NA	OQ440398, OL875043, MN313681, MN313680, MN313679, KU933151, KU933150, KX555564, KU925233	NA	NA	NA	NA	NA	NA
Hodotermopsis sjostedti	NA	NA	NA	NA	NA	OM646553, LC606589, LC606588, LC606587, LC606586, LC606585, LC606584, KP026259	NA	NA	NA	NA	NA	NA
Kalotermes approximatus	NA	NA	NA	NA	NA	PP112326, PP112325, PP112324, OM991351	NA	NA	NA	NA	NA	NA
Kalotermes flavicollis	NA	NA	NA	NA	PP112323, PP112322, OL875030, OK506054, MZ029062, MZ029061, MT484288, MF589164, MF589163, MF589162	PP112323, PP112322, OL875030, OK506054, MF589164, MF589163, MF589162, MF589161, MF589158, MF589157	NA	NA	NA	NA	NA	NA
Kalotermes italicus	NA	NA	NA	NA	NA	MF589160, MF589159, MF589153, MF589152, MF589151, MF589150, MF589146, MF589145,	NA	NA	NA	NA	NA	NA

Species	Gene											
	12S	***16S***	***ATP6***	***ATP8***	***COX1***	***COX2***	***CYTB***	***ND2***	***ND4***	***ND4L***	***ND5***	***ND6***
						MF589144, MF589143						
Labiotermes labralis	NA	NA	OQ980234, OL875034, KY436202, KY436201, KP026292	NA	OQ980234, OL875034, OK310886, OK310885, KY436202, KY436201, KP026292	OQ980234, OL875034, MN803316, MH068000, MH067999, MH067998, MH067997, KY436202, KY436201, KP026292	NA	NA	NA	NA	NA	NA
Longicaputermes sinaicus	NA	NA	OM991368, OM991367, OM991366, OM991365, OM991364, OM991363	NA	OM991368, OM991367, OM991366, OM991365, OM991364, OM991363	OM991368, OM991367, OM991366, OM991365, OM991364, OM991363, KC914290, KC914289, KC914288, KC914287	NA	NA	NA	NA	OM991368, OM991367, OM991366, OM991365, OM991364, OM991363	OM991368, OM991367, OM991366, OM991365, OM991364, OM991363
Macrotermes annandalei	NA	NA	NA	NA	LC481541, LC481540, KU900579, KY224518	NA	NA	NA	NA	NA	NA	NA
Macrotermes subhyalinus	NA	NA	NA	NA	OR782849, OR782848, OR782847, OR782846, OR782845, OR782844, OR782843, OR782842, OR782841, OR782840	OR653920, KY224559, JF302855, JF302854, JX144937	NA	NA	NA	NA	NA	NA
Margattea nimbata	NA	NA	NA	NA	LC750828, LC750827, LC750826, MW970261, MW970260, MW970259, MW970258, MW970257	NA	NA	NA	NA	NA	NA	NA

Species	Gene											
	12S	***16S***	***ATP6***	***ATP8***	***COX1***	***COX2***	***CYTB***	***ND2***	***ND4***	***ND4L***	***ND5***	***ND6***
Margattea ogatai	NA	NA	NA	NA	NA	LC750841, LC750840, LC750839, LC750838, LC750837, LC750836	NA	NA	NA	NA	NA	NA
Margattea satsumana	NA	NA	NA	NA	NA	LC750835, LC750834, LC750833, LC750832	NA	NA	NA	NA	NA	NA
Microcerotermes crassus	NA	NA	OQ980220, OQ955593, OQ908860, KY224690, KY224496, KY224461, KY224445, KY224428	NA	OQ980220, OQ955593, OQ908860, KY224690, KY224496, KY224461, KY224445, KY224428	OQ980220, OQ955593, OQ908860, KY224690, KY224496, KY224461, KY224445, KY224428	NA	NA	NA	NA	NA	NA
Microcerotermes papuanus	NA	NA	OQ991901, OQ984045, OQ933859, OK163848	NA	NA	NA	NA	NA	NA	NA	OQ991901, OQ984045, OQ933859, OK163848	OQ991901, OQ984045, OQ933859, OK163848
Microcerotermes parvus	NA	NA	OQ991905, OQ947860, OQ947858, OQ933865, KP091693, KP091692, KP091691, KP091690, KP091689, KP091688	NA	OQ991905, OQ947860, OQ947858, OQ933865, KP091693, KP091692, KP091691, KP091690, KP091689, KP091688	OQ991905, OQ947860, OQ947858, OQ933865, KP091693, KP091692, KP091691, KP091690, KP091689, KP091688	NA	NA	NA	NA	OQ991905, OQ947860, OQ947858, OQ933865	OQ991905, OQ947860, OQ947858, OQ933865
Microcerotermes sikorae	NA	NA	NA	NA	NA	OQ130290, OQ130288, OQ130285, FJ818891, FJ818890, FJ818889, FJ818888, FJ818887, FJ818886, FJ818885	NA	NA	NA	NA	NA	NA
Microcerotermes subtilis	NA	NA	OQ130294, OQ130293,	NA	OQ130294, OQ130293,	OQ130294, OQ130293,	NA	NA	NA	NA	OQ130294, OQ130293,	OQ130294, OQ130293,

Species	Gene											
	12S	***16S***	***ATP6***	***ATP8***	***COX1***	***COX2***	***CYTB***	***ND2***	***ND4***	***ND4L***	***ND5***	***ND6***
			OQ130276, OQ130275		OQ130276, OQ130275	OQ130276, OQ130275					OQ130276, OQ130275	OQ130276, OQ130275
Microtermes obesi	NA	NA	NA	NA	OP741194, KY224632, KY224524, KY224504	NA	NA	NA	NA	NA	NA	NA
Nasutitermes canaliculatus	NA	NA	OQ675029, OQ612667, OQ612666, OQ612665, OQ612664, OQ612663, OQ612662	NA	OQ675029, OQ612667, OQ612666, OQ612665, OQ612664, OQ612663, OQ612662	OQ675029, OQ612667, OQ612666, OQ612665, OQ612664, OQ612663, OQ612662	NA	NA	NA	NA	OQ675029, OQ612667, OQ612666, OQ612665, OQ612664, OQ612663, OQ612662	OQ675029, OQ612667, OQ612666, OQ612665, OQ612664, OQ612663, OQ612662
Nasutitermes corniger	NA	NA	OL875055, KP091693, KP091692, KP091691, KP091690, KP091689, KP091688	NA	OL875055, KP091693, KP091692, KP091691, KP091690, KP091689, KP091688	OL875055, MH577203, KP091693, KP091692, KP091691, KP091690, KP091689, KP091688	NA	NA	NA	NA	NA	NA
Nasutitermes ephratae	NA	NA	NA	NA	NA	MH577205, MH067986, MH067985, MH067984, MH067983, MF176392	NA	NA	NA	NA	NA	NA
Nasutitermes matangensis	NA	NA	KY224730, KY224715, KY224639, KY224599, KY224561, KY224423, KY224422	NA	KY224730, KY224715, KY224639, KY224599, KY224561, KY224423, KY224422	KY224730, KY224715, KY224639, KY224599, KY224561, KY224423, KY224422	NA	NA	NA	NA	NA	NA
Nasutitermes nigritus	NA	NA	OQ675027, OQ612655, OQ612654, OQ612653, OQ612652	NA	OQ675027, OQ612655, OQ612654, OQ612653, OQ612652	OQ675027, OQ612655, OQ612654, OQ612653, OQ612652, FJ818922, FJ818921, FJ818920, FJ818919, FJ818918	NA	NA	NA	NA	OQ675027, OQ612655, OQ612654, OQ612653, OQ612652	OQ675027, OQ612655, OQ612654, OQ612653, OQ612652

Species	Gene											
	12S	***16S***	***ATP6***	***ATP8***	***COX1***	***COX2***	***CYTB***	***ND2***	***ND4***	***ND4L***	***ND5***	***ND6***
Nasutitermes similis	NA	NA	NA	NA	NA	MH577216, MH067981, MH067980, MH067979, MH067978, KY224557, KY238295	NA	NA	NA	NA	NA	NA
Neocapritermes taracua	NA	NA	NA	NA	NA	OQ980239, KP091693, KP091692, KP091691, KP091690, KP091689, KP091688, MH068014, MH068013, KY224527	NA	NA	NA	NA	NA	NA
Odontotermes formosanus	NA	NA	NA	NA	OL875037, MZ429083, MZ429077, MZ424473, MW535144, MW535143, KP026254	NA	NA	NA	NA	NA	NA	NA
Odontotermes obesus	NA	NA	NA	NA	PQ044821, PP177528, OP781936, OP741193, OP579103, MZ823814, KY224493, KY224406, KY238293	OM630470, KY224493, KY224406, KY238293	NA	NA	NA	NA	NA	NA
Opisthoplatia orientalis	NA	NA	NA	NA	LC793871, MW649982, MW649981, MW535126, MW535112, MW535110, MW450974, MW450969, KY748280, MG882239	NA	NA	NA	NA	NA	NA	NA
Panchlora nivea	NA	NA	NA	NA	MT338275, MT338274,	NA	NA	NA	NA	NA	NA	NA

Species	Gene										
	12S	***16S***	***ATP6***	***ATP8***	***COX1***	***COX2***	***CYTB***	***ND2***	***ND4***	***ND4L***	***ND5***	***ND6***
					MT338273, MT338272, MT338271, MT338270, MT338269, MT338268, MT338267, MT338266							
Panesthia sloanei	NA	NA	MW996597, MW996596, MW996595, MW996594	NA	MW996597, MW996596, MW996595, MW996594	MW996597, MW996596, MW996595, MW996594	NA	NA	NA	NA	NA	NA
Panesthia tryoni	NA	NA	MW996604, MW996603, MW996602, MW996601, MW996600, MW996599, MW996598	NA	MW996604, MW996603, MW996602, MW996601, MW996600, MW996599, MW996598	MW996604, MW996603, MW996602, MW996601, MW996600, MW996599, MW996598	NA	NA	NA	NA	NA	NA
Patawatermes nigripunctatus	NA	NA	OQ998964, MW773532, KY224476, KY224420	NA	OQ998964, MW773532, KY224476, KY224420	OQ998964, MW773532, KY224476, KY224420	NA	NA	NA	NA	NA	NA
Periplaneta americana	NA	NA	NA	NA	PP854574, PP854573, PP854572, LC793868, PP087113, PP087112, PP087111, PP087110, PP087109, PP087108	PP101121, PP101120, PP101119, PP101118, PP101117, PP101116, PP101115, PP101114, PP101113, PP101112	NA	NA	NA	NA	NA	NA
Periplaneta australasiae	NA	NA	NA	NA	LC793869, MG882181, KX640826, KX640825	NA	NA	NA	NA	NA	NA	NA
Periplaneta brunnea	NA	NA	NA	NA	LC793870, MT498808, MG010455, MG882182	NA	NA	NA	NA	NA	NA	NA
Periplaneta lateralis	NA	NA	NA	NA	LC793873, PP216565, PP216564, PP216563,	NA	NA	NA	NA	NA	NA	NA

Species	Gene										
	12S	***16S***	***ATP6***	***ATP8***	***COX1***	***COX2***	***CYTB***	***ND2***	***ND4***	***ND4L***	***ND5***	***ND6***
					PP216562,<br>PP216561,<br>PP216560,<br>PP216559,<br>PP216558,<br>PP216557							
Pseudacanthotermes militaris	NA	NA	NA	NA	OQ947859,<br>OQ703627,<br>OQ703626,<br>OQ703625,<br>KY224517	NA	NA	NA	NA	NA	NA	NA
Pseudocapritermes sowerbyi	NA	NA	NA	NA	MT445563,<br>MT445562,<br>MT445561,<br>MT445560,<br>MT445559,<br>MT445558,<br>MT445557,<br>MT434008	NA	NA	NA	NA	NA	NA	NA
Pycnoscelus surinamensis	NA	NA	NA	NA	OR223810,<br>OR223809,<br>OR223808,<br>OR223807,<br>OL589366,<br>MW051035,<br>MW051034,<br>MW535142,<br>MW535141,<br>MW535140	NA	NA	NA	NA	NA	NA	NA
Reticulitermes flavipes	NA	NA	OL875053,<br>EF206317,<br>EF206316,<br>EF206314,<br>KU925236	NA	OM421644,<br>OL875053,<br>EF206317,<br>EF206316,<br>EF206314	OL875053,<br>EF206317,<br>EF206316,<br>EF206314,<br>KU925236	NA	NA	NA	NA	OL875053,<br>EF206317,<br>EF206316,<br>EF206314	OL875053,<br>EF206317,<br>EF206316,<br>EF206314
Reticulitermes urbis	NA	NA	NA	NA	NA	MF374832,<br>MF374831,<br>MF374830,<br>MF374829,<br>MF374828,<br>MF374827,<br>MF374826,<br>MF374825	NA	NA	NA	NA	NA	NA
Silvestritermes heyeri	NA	NA	NA	NA	NA	MH068008,<br>MH068007,<br>MH068006,	NA	NA	NA	NA	NA	NA

Species	Gene											
	12S	*16S*	*ATP6*	*ATP8*	*COX1*	*COX2*	*CYTB*	*ND2*	*ND4*	*ND4L*	*ND5*	*ND6*
						MH068005, KY224619, KY224492						
Termes fatalis	NA	NA	KY224726, KY224572, KY224507, KY224457	NA	KY224726, KY224572, KY224507, KY224457	MH068017, MH068016, MH068015, KY224726, KY224572, KY224507, KY224457	NA	NA	NA	NA	NA	NA
Trinervitermes geminatus	NA	NA	NA	NA	OR782883, OR782882, OR782881, OR782880, OR782879, OQ555367, MZ029081, MZ029080, MZ029079, MT484292	NA	NA	NA	NA	NA	NA	NA
Trinervitermes occidentalis	NA	NA	NA	NA	OR782889, OR782888, OR782887, OR782886, OR782885, OR782884, MZ029083, MZ029082	NA	NA	NA	NA	NA	NA	NA
Zootermopsis angusticollis	NA	NA	NA	NA	OQ567494, OQ411246, OQ411245, JX144932	NA	NA	NA	NA	NA	NA	NA
Zootermopsis nevadensis	NA	NA	OQ559113, OQ434263, OQ434262, KJ958410	NA	NA	NA	NA	NA	NA	NA	OQ559113, OQ434263, OQ434262, KJ958410	OQ559113, OQ434263, OQ434262, KJ958410
**Mantodea**												
Mantis religiosa	NA	KX434836, MZ153073, MN356097, KU201317	NA	KX434836, MZ153073, MN356097, KU201317	MG377394, OP862442, KX434836, MZ153073, MT011513, MN356097, KM529415, HQ981410,	NA	NA	KX434836, MZ153073, MN356097, KU201317	NA	NA	NA	KX434836, MZ153073, MN356097, KU201317

Species	Gene										
	12S	*16S*	*ATP6*	*ATP8*	*COX1*	*COX2*	*CYTB*	*ND2*	*ND4*	*ND4L*	*ND5*	*ND6*
					KR148854, KR146939							
Rhombodera valida	NA	KX434848, KX434869, OP168282, OP168281, KX611804	NA	KX434848, KX434869, OP168282, OP168281, KX611804	KX434848, KX434869, KX611804	NA	NA	KX434848, KX434869, OP168282, OP168281, KX611804	NA	NA	NA	KX434848, KX434869, OP168282, OP168281, KX611804
Statilia maculata	NA	KX434832, KX434809, OK542390, KX900484	NA	KX434832, KX434809, OK542390, KX900484	KX434832, KX434809, OK542390, OP050279, OL913123, OL913122, OL913121, OL913120, MZ836002	NA	NA	KX434832, KX434809, OK542390, KX900484	NA	NA	NA	KX434832, KX434809, OK542390, KX900484
**Plecoptera**												
Claassenia magna	NA	NA	NA	NA	OL664057, OL664056, OL664055, OL664054, OL664053, OL664052, OK012602, MN419914	NA	NA	NA	NA	NA	NA	NA
Dictyogenus fontium	NA	NA	NA	NA	OR733994, OR733991, MZ027511, MZ027510, MZ027509, MZ027508, MZ027507, MZ027506, MZ027505, MZ027504	NA	NA	NA	NA	NA	NA	NA
Leuctra fusca	NA	NA	NA	NA	OR733838, MT872701, MN850722, MT483025, MT483024, MT483023, MT483022, MT483021, MT483020, MT483019	NA	NA	NA	NA	NA	NA	NA

**Species**	**Gene**											
	12S	***16S***	***ATP6***	***ATP8***	***COX1***	***COX2***	***CYTB***	***ND2***	***ND4***	***ND4L***	***ND5***	***ND6***
Nemurella pictetii	NA	NA	NA	NA	OR733759,<br>OR601702,<br>MZ046710,<br>MT862398	NA	NA	NA	NA	NA	NA	NA
Neoperla arambourgana	NA	NA	NA	NA	OR031168,<br>OR031148,<br>OR031147,<br>OR031146,<br>OR031143	NA	NA	NA	OR034778,<br>OR034777,<br>OR034757,<br>OR034756,<br>OR034755,<br>OR034752	OR034913,<br>OR034912,<br>OR034892,<br>OR034891,<br>OR034890,<br>OR034887	OR034913,<br>OR034912,<br>OR034892,<br>OR034891,<br>OR034890,<br>OR034887	OR034977,<br>OR034976,<br>OR034956,<br>OR034955,<br>OR034954,<br>OR034951
Neoperla burgeoni	NA	NA	NA	NA	OR031186,<br>OR031185,<br>OR031166,<br>OR031165,<br>OR031164,<br>OQ992288,<br>OQ992287,<br>OQ992286,<br>OQ992285,<br>OQ992284	NA	NA	NA	OR034796,<br>OR034795,<br>OR034775,<br>OR034774,<br>OR034773	OR034930,<br>OR034929,<br>OR034910,<br>OR034909,<br>OR034908	OR034930,<br>OR034929,<br>OR034910,<br>OR034909,<br>OR034908	OR034992,<br>OR034974,<br>OR034973,<br>OR034972
Neoperla excisa	NA	NA	NA	NA	OR031195,<br>OR031194,<br>OR031184,<br>OQ992491	NA	NA	NA	NA	NA	NA	NA
Neoperla panafricana	NA	NA	NA	NA	OR031204,<br>OR031183,<br>OR031182,<br>OR031181,<br>OR031180,<br>OR031179,<br>OR031149,<br>OQ992460,<br>OQ992417,<br>OQ992416	NA	NA	NA	OR034793,<br>OR034792,<br>OR034791,<br>OR034790,<br>OR034789,<br>OR034758	OR034927,<br>OR034926,<br>OR034925,<br>OR034924,<br>OR034893,<br>OR034884	OR034927,<br>OR034926,<br>OR034925,<br>OR034924,<br>OR034893,<br>OR034884	OR034990,<br>OR034989,<br>OR034988,<br>OR034987,<br>OR034957
Neoperla socia	NA	NA	NA	NA	OR031189,<br>OR031188,<br>OR031187,<br>OQ992490	NA	NA	NA	NA	NA	NA	NA
Protonemura lateralis	NA	NA	NA	NA	OR733851,<br>OR733802,<br>OR733771,<br>OR733766,<br>OR733752,<br>MZ027532,<br>MZ027531,	NA	NA	NA	NA	NA	NA	NA

Species	Gene										
	12S	*16S*	*ATP6*	*ATP8*	*COX1*	*COX2*	*CYTB*	*ND2*	*ND4*	*ND4L*	*ND5*	*ND6*
					MZ027530, MZ027529, MZ027528							
Siphonoperla montana	NA	NA	NA	NA	MT482900, MT482899, MT482898, MT482897	NA	NA	NA	NA	NA	NA	NA
Tyrrhenoleuctra tangerina	NA	NA	NA	NA	OQ633073, OQ633072, OQ633071, OQ633070, OQ633069	NA	NA	NA	NA	NA	NA	NA
Zwicknia bifrons	NA	NA	NA	NA	OR733940, OR733931, MT872688, MT872687	NA	NA	NA	NA	NA	NA	NA
**Orthoptera**												
Acheta domesticus	N/A	OK504623, MZ440654, MK204368, JX269094, KR903672, AF514462, AF248698, AF020299, Z97627	NA	NA	OK504623, MZ440654, MZ197987, MT859331	NA	N/A	N/A	NA	NA	N/A	NA
Aiolopus thalassinus	MK903555, KY236114, JQ247667, AB497590, AY560535, AY352427	N/A	NA	NA	OQ658232, OQ658228, OQ658227, PP911326, OR515637, OL589385, OP392008, ON368926, MZ436010, OL343337	NA	MK903555, KY236114, EU366075, EU366076, HQ712024, EF151876, AB497587	AB497589	NA	NA	N/A	NA
Antaxius spinibrachius	N/A	N/A	NA	NA	N/A	NA	N/A	OR592055, OR592054, OR592053, OR592052, OR592051, OR592050, OR592049, OR592048,	NA	NA	N/A	NA

Species	Gene											
	12S	***16S***	***ATP6***	***ATP8***	***COX1***	***COX2***	***CYTB***	***ND2***	***ND4***	***ND4L***	***ND5***	***ND6***
								OR592047, OR592046				
Anterastes babadaghi	N/A	MW241321, MN138365, KR051820, KR051819, KR051818, KR051817, KR051814, KR051808, HQ850980, HQ850978	NA	NA	N/A	NA	N/A	MN138365	NA	NA	N/A	NA
Apholidoptera pietschmanni	N/A	N/A	NA	NA	N/A	NA	N/A	MW261421, MW261420, MW261419, MW261418, MW261417, MW261416	NA	NA	N/A	NA
Arcyptera meridionalis	N/A	N/A	NA	NA	N/A	NA	MF997490, MN083204, MG838886, JQ996600	N/A	NA	NA	N/A	NA
Arminda brunneri	N/A	N/A	NA	NA	N/A	NA	N/A	N/A	NA	NA	EF134464, EF134463, EF134462, EF134461, EF134460, EF134459	NA
Arminda fuerteventurae	N/A	N/A	NA	NA	N/A	NA	N/A	N/A	NA	NA	EF134455, EF134454, EF134453, EF134452	NA
Betiscoides meridionalis	N/A	N/A	NA	NA	N/A	NA	N/A	N/A	NA	NA	MG243894, MG243841, MG243840, MG243839, MG243837	NA
Betiscoides muris	N/A	N/A		NA	N/A	NA	N/A	N/A	NA	NA	PP417669, PP417668, PP417667, PP417666, PP417665	NA
Betiscoides nova	N/A	N/A		NA	N/A	NA	N/A	N/A	NA	NA	PP417676, PP417675, PP417674,	NA

Species	Gene										
	12S	***16S***	***ATP6***	***ATP8***	***COX1***	***COX2***	***CYTB***	***ND2***	***ND4***	***ND4L***	***ND5***	***ND6***
											PP417673,<br>PP417672,<br>PP417671,<br>PP417670	
Bryodema gebleri	N/A	N/A		NA	N/A	NA	N/A	N/A	NA	NA	OQ470637,<br>JQ513126,<br>JQ513125,<br>JQ513124	NA
Bryodemella tuberculata	N/A	N/A		NA	PP669995,<br>MW263333,<br>MW263332,<br>MW263331,<br>MW263330,<br>MW263329,<br>MW263328,<br>MW263327,<br>MW263326,<br>MW263325	NA	N/A	N/A	NA	NA	PP669995,<br>MN083196,<br>JQ513131,<br>JQ513130,<br>JQ513129	NA
Calliphona alluaudi	N/A	EF515135,<br>EF515134,<br>EF515133,<br>EF515132,<br>EF515131,<br>EF515130,<br>EF515129,<br>EF515128,<br>EF515127,<br>EF515126		NA	N/A	NA	N/A	N/A	NA	NA	N/A	NA
Calliphona palmensis	N/A	EF515148,<br>EF515147,<br>EF515146,<br>EF515145,<br>EF515144,<br>EF515143,<br>EF515142		NA	N/A	NA	N/A	N/A	NA	NA	N/A	NA
Calliptamus barbarus	N/A	N/A	NA	NA	ON426211,<br>ON426210,<br>ON426209,<br>ON426208,<br>ON426207,<br>ON426206,<br>ON426205,<br>ON426204,<br>MZ714070,<br>MZ714069	NA	MT985324,<br>MN083183,<br>DQ366770,<br>JX033932,<br>DQ366833	N/A	NA	NA	N/A	NA

Species	Gene											
	12S	*16S*	*ATP6*	*ATP8*	*COX1*	*COX2*	*CYTB*	*ND2*	*ND4*	*ND4L*	*ND5*	*ND6*
Calliptamus italicus	N/A	N/A	NA	NA	MZ714109, MZ714108, MZ714107, MZ714106, MZ714105, MZ714104, MZ714103, MZ714102, MZ714101, MZ714100	NA	MN864046, MN864045, MN864044, MN864043, MN864042, MN864041, MN864040, MN864039, MN864038, MN864037	N/A	NA	NA	N/A	NA
Ceracris fasciata	N/A	N/A	NA	NA	N/A	NA	MK903585, KP872953, JQ996615, DQ365919, DQ365918	N/A	NA	NA	N/A	NA
Ceracris nigricornis	MF997464, MK903558, AY995322, AY995317	MF997464, MK903558, JQ065102, AY995326, AY995325, AY995322, AY995317	NA	NA	MF997464, MT325835, MT325834, MT325833, MK903558, MW056480, MW056479, MW056478, MW053536	NA	MF997464, MK903558, AY157540, JQ996616, JQ996604, DQ365902	N/A	NA	NA	N/A	NA
Chizuella bonneti	N/A	LC801691, MH685924, KX057723, EF198432	NA	NA	N/A	NA	N/A	N/A	NA	NA	N/A	NA
Choroedocus violaceiepes	N/A	N/A	NA	NA	N/A	NA	N/A	N/A	NA	NA	N/A	NA
Choroedocus violaceipes	N/A	MK903559, KY236113, DQ366759, JQ065103, DQ366821	NA	NA	N/A	NA	MK903559, KY236113, DQ366759, DQ366821	MK903559, KY236113, DQ092568, JQ283252	NA	NA	N/A	NA
Chorthippus parallelus	MT166302, MT166301, MT166300, MT166299, MT166298, KX426768, AY803353	MT166302, MT166301, MT166300, MT166299, MT166298, KX426822, AY803353, JQ580931, JQ580930	MT166302, MT166301, MT166300, MT166299, MT166298, KX426822, AY803353, JQ580931, JQ580930	NA	MT166302, MT166301, MT166300, MT166299, MT166298, AY803353	NA	MW232411, MW232410, MW232409, MW232408, MW232407, MW232406, MW232405, MW232404, MW232403, MW232402	MT166302, MT166301, MT166300, MT166299, MT166298, AY803353, X95575, X95574	NA	NA	MT166302, MT166301, MT166300, MT166299, MT166298, DQ230758, AY803353, Y14813	NA

Species	Gene										
	12S	***16S***	***ATP6***	***ATP8***	***COX1***	***COX2***	***CYTB***	***ND2***	***ND4***	***ND4L***	***ND5***	***ND6***
Ducetia japonica	N/A	KY612457, KU885974, HG810325, EF685926, EF198438	NA	NA	PP389423, OQ053066, MT679148, MT679147, MT679146, MT679145, MT679144, MT679143, MT679142, MT679141	NA	N/A	N/A	NA	NA	N/A	NA
Eupholidoptera smyrnensis	N/A	MW241225, MW241224, MW241223, MW241222, KC852311, KC852310, KC852309, EU120794	NA	NA	N/A	NA	N/A	N/A	NA	NA	N/A	NA
Gampsocleis sedakovii	N/A	MK903561, JQ065104, EF198424, EF198423	NA	NA	N/A	NA	N/A	N/A	NA	NA	N/A	NA
Gryllus bimaculatus	MT993975, MZ440656, KR903835, AY905292	N/A	NA	NA	MT993975, OP142874, OP142873, OP142872, OP142871, OP142870, OP142869, OP142868, OP142867, OP142866	NA	MT993975, EF093752, EF093751, EF093750, EF093749, EF093748, EF093747, EF093746, EF093745, EF093744	N/A	NA	NA	N/A	NA
Haplotropis brunneriana	N/A	KC894753, MK903563, DQ366755, JQ065106, DQ366816, AY379749	NA	NA	N/A	NA	KC894753, MK903563, MT011664, DQ366755, AY382870, DQ366816	N/A	NA	NA	N/A	NA
Isophya cania	N/A	N/A	NA	NA	N/A	NA	N/A	KX026779, KX026778, KX026777, KX026742, KX026741, KX026740, KX026739	NA	NA	N/A	NA

Species	Gene											
	12S	***16S***	***ATP6***	***ATP8***	***COX1***	***COX2***	***CYTB***	***ND2***	***ND4***	***ND4L***	***ND5***	***ND6***
Isophya rectipennis	N/A	N/A	NA	NA	N/A	NA	N/A	KX026773, KX026772, KX026770, KX026765, KX026764, KX026759, KX026758	NA	NA	N/A	NA
Isophya rhodopensis	N/A	N/A	NA	NA	N/A	NA	N/A	KX026753, KX026752, KX026751, KX026748, KX026747	NA	NA	N/A	NA
Isophya stenocauda	N/A	N/A	NA	NA	N/A	NA	N/A	KX026749, KX026737, KX026736, KX026734, KX026733	NA	NA	N/A	NA
Isophya thracica	N/A	N/A	NA	NA	N/A	NA	N/A	KX026763, KX026762, KX026761, KX026760	NA	NA	N/A	NA
Locusta migratoria	HQ260647, HM219224, JN858212, JN858211, JN858210, JN858209, JN858208, JN858207, JN858206, JN858205	LC801762, LC801681, KX977407, HQ260647, HM219224, JN858212, JN858211, JN858210, JN858209, JN858208	HQ260647, HM219224, JN858212, JN858211, JN858210, JN858209, JN858208	NA	OL663229, OL663228, OL663227, MT311114, MN395672, KX170937, EU287446, EF368367, EF368366, GU344101	NA	KX170937, EU366091, EU366092, EU287446, HQ260647, HM219224, GU344101, JN858212, JN858211, JN858210	KX170937, EU287446, HQ260647, HM219224, GU344101, AB497593, AB497589, AB497585, AB497457, AB497456	NA	NA	KX170937, EU287446, KM384871, AY324457, AY324456, AY324455, HQ260647, HM219224, GU344101, EF546761	NA
Loxoblemmus doenitzi	N/A	LC801746, MK903567, KX057721, KX673202, JQ065108	NA	NA	MZ472885, MZ472884, MZ472883, MZ472882, MZ472881, MZ472880, MZ701948, MK903567, KX057721, KX673202	NA	MZ681945, MK903567, KX057721, KX673202	MK903567, KX057721, KX673202, JQ283282	NA	NA	N/A	NA
Nomadacris japonica	N/A	N/A	NA	NA	OR672728, OR672727, OR672726, OR672725,	NA	N/A	MK059455, MF113246, DQ092569, JQ283265	NA	NA	N/A	NA

Species	Gene										
	12S	*16S*	*ATP6*	*ATP8*	*COX1*	*COX2*	*CYTB*	*ND2*	*ND4*	*ND4L*	*ND5*	*ND6*
					OR672724, OR672723, OR672722, OR672721, OR672720, OR672719							
Oedaleus decorus	N/A	N/A	NA	NA	N/A	NA	PP690437, PP690436, PP690435, PP690434, PP690433, PP690432, PP690431, PP690430, PP690429, PP690428	N/A	NA	NA	N/A	NA
Oedaleus infernalis	N/A	N/A	NA	NA	OL664387, OL664386, OL664385, OL343148, OL343147, KR604688	NA	EU366095, EU366096, HQ712033, KR604688, KC484981, KC484980, KC484979, KC484978, KC484977, KC484976	AB497593	NA	NA	N/A	NA
Oedipoda caerulescens	N/A	N/A	NA	NA	OQ282994, OQ615379, MT311122, KM816665, KM816664, KM816663, KM816655	NA	N/A	N/A	NA	NA	OQ282994, OQ615379, EU266744, JX244441	NA
Omocestus haemorrhoidalis	N/A	N/A	NA	NA	N/A	NA	MK903570, EU366097, EU366098, AY738382, JQ996592	N/A	NA	NA	N/A	NA
Pachyrhamma edwardsii	OR551729, OR551719, HM594541, HM594540	OR551729, OR551719, HM594509, HM594508	NA	NA	N/A	NA	N/A	N/A	NA	NA	N/A	NA
Parapholidoptera distincta	N/A	N/A	NA	NA	N/A	NA	N/A	MW261428, MW261427,	NA	NA	N/A	NA

Species	Gene										
	12S	***16S***	***ATP6***	***ATP8***	***COX1***	***COX2***	***CYTB***	***ND2***	***ND4***	***ND4L***	***ND5***	***ND6***
								MW261426, MW261425				
Parapholidoptera salmani	N/A	N/A	NA	NA	N/A	NA	N/A	MW261366, MW261365, MW261364, MW261363, MW261362, MW261361, MW261360, MW261359, MW261358	NA	NA	N/A	NA
Parapholidoptera signata	N/A	N/A	NA	NA	N/A	NA	N/A	MW261373, MW261372, MW261371, MW261370, MW261369, MW261368, MW261367	NA	NA	N/A	NA
Pezotettix giornae	N/A	N/A	NA	NA	N/A	NA	N/A	N/A	NA	NA	KM384856, EF134472, EF134471, EF134470, JX244446, JX033939	NA
Phaneroptera falcata	N/A	LC801735, LC801663, KY458227, AM886660, EF198437	NA	NA	N/A	NA	N/A	N/A	NA	NA	N/A	NA
Pholidoptera brevipes	N/A	N/A	NA	NA	N/A	NA	N/A	MW261432, MW261431, MW261430, MW261429	NA	NA	N/A	NA
Pholidoptera fallax	N/A	N/A	NA	NA	N/A	NA	N/A	MW261351, MW261350, MW261349, MW261348, MW261347	NA	NA	N/A	NA
Pholidoptera griseoaptera	N/A	N/A	NA	NA	N/A	NA	N/A	MT872693, MW261346, MW261345, MW261344, MW261343	NA	NA	N/A	NA
Poecilimon brunneri	AM886640, AM886639,	AM886640, AM886639,	NA	NA	N/A	NA	N/A	N/A	NA	NA	N/A	NA

Species	Gene											
	12S	***16S***	***ATP6***	***ATP8***	***COX1***	***COX2***	***CYTB***	***ND2***	***ND4***	***ND4L***	***ND5***	***ND6***
	AM886637,<br>AM886632,<br>AM886629,<br>AM886550	AM886637,<br>AM886632,<br>AM886629,<br>AM886550										
Poecilimon ciplaki	N/A	N/A	NA	NA	N/A	NA	N/A	MH168592,<br>MH168591,<br>MH168581,<br>MH168580	NA	NA	N/A	NA
Poecilimon cretensis	N/A	N/A	NA	NA	N/A	NA	N/A	OQ427618,<br>MW796386,<br>MW796385,<br>MW796384,<br>MW796383,<br>MT416238,<br>MN114200,<br>MN114199,<br>MN114198	NA	NA	N/A	NA
Poecilimon isozonatus	N/A	N/A	NA	NA	N/A	NA	N/A	OP244051,<br>MH168594,<br>MH168593,<br>MH168585,<br>MH168584,<br>MH168583,<br>MH168582	NA	NA	N/A	NA
Poecilimon jonicus	AM886616,<br>AM886606,<br>AM886571,<br>AM886567,<br>AM886563,<br>AM886542	AM886616,<br>AM886606,<br>AM886571,<br>AM886567,<br>AM886563,<br>AM886542	NA	NA	N/A	NA	N/A	MN114192,<br>MN114191,<br>MN114190,<br>MN114189,<br>MN114188,<br>MN114187,<br>MN114186	NA	NA	N/A	NA
Poecilimon luschani	N/A	MK757458,<br>KM376456,<br>KM376455,<br>KM376454,<br>KM376453,<br>KM376452,<br>KM376451,<br>KM376450,<br>KF379583,<br>KF379582	NA	NA	N/A	NA	N/A	N/A	NA	NA	N/A	NA
Poecilimon macedonicus	AM886579,<br>AM886575,<br>AM886554,<br>AM886552	AM886579,<br>AM886575,<br>AM886554,<br>AM886552	NA	NA	N/A	NA	N/A	N/A	NA	NA	N/A	NA

Species	Gene											
	12S	***16S***	***ATP6***	***ATP8***	***COX1***	***COX2***	***CYTB***	***ND2***	***ND4***	***ND4L***	***ND5***	***ND6***
Poecilimon orbelicus	N/A	KF286959,<br>KF286958,<br>AM886583,<br>AM886553	NA	NA	N/A	NA	N/A	N/A	NA	NA	N/A	NA
Poecilimon ornatus	AM886581,<br>AM886580,<br>AM886574,<br>AM886562	N/A	NA	NA	N/A	NA	N/A	N/A	NA	NA	N/A	NA
Poecilimon tessellatus	N/A	N/A	NA	NA	N/A	NA	N/A	MN114192,<br>MN114191	NA	NA	N/A	NA
Poecilimon tuncayi	N/A	KM376450,<br>KF286971,<br>KF286970,<br>KF286969,<br>KF286968,<br>KF286967,<br>KF286966,<br>KF286965,<br>KF286964,<br>AM886667	NA	NA	N/A	NA	N/A	N/A	NA	NA	N/A	NA
Poecilimon zimmeri	AM886642,<br>AM886582,<br>AM886577,<br>AM886576,<br>AM886543	AM886642,<br>AM886582,<br>AM886577,<br>AM886576,<br>AM886543	NA	NA	N/A	NA	N/A	N/A	NA	NA	N/A	NA
Poecilimon zonatus	N/A	N/A	NA	NA	N/A	NA	N/A	MH168594,<br>MH168593,<br>MH168592,<br>MH168591	NA	NA	N/A	NA
Psalmatophanes barretoi	N/A	EF515154,<br>EF515153,<br>EF515152,<br>EF515151,<br>EF515150,<br>EF515149	NA	NA	N/A	NA	N/A	N/A	NA	NA	N/A	NA
Psorodonotus venosus	N/A	MW241293,<br>MW241291,<br>MN138365,<br>MK951778,<br>KU602074,<br>KU602073,<br>KU602072,<br>KU602071,<br>KU602070,<br>KU602069	NA	NA	N/A	NA	N/A	MW261339,<br>MW261338,<br>MN138365,<br>MK951778	NA	NA	N/A	NA

**Species**	**Gene**											
	12S	***16S***	***ATP6***	***ATP8***	***COX1***	***COX2***	***CYTB***	***ND2***	***ND4***	***ND4L***	***ND5***	***ND6***
Ruspolia lineosa	N/A	N/A	NA	NA	N/A	NA	MK903572, KX057729, JQ793771, JQ793770, JQ793769, JQ793768, JQ793767, JQ793766, JQ793765, JQ793764	N/A	NA	NA	N/A	NA
Schistocerca americana	N/A	OM650688, KY980817, AF155553, AY605943	OM650688, KY980817, AF155553, AY605943	NA	N/A	NA	N/A	N/A	NA	NA	N/A	NA
Schistocerca cancellata	N/A	ON530889, KY980821, FJ393927, FJ393926, AY605945	NA	NA	N/A	NA	N/A	N/A	NA	NA	N/A	NA
Schistocerca damnifica	KY980783, KM504205, KM504204, AY605936	KY980818, KM504205, KM504204, AY605936	NA	NA	N/A	NA	N/A	N/A	NA	NA	N/A	NA
Schistocerca gregaria	N/A	ON764804, KY980799, EU203966, EU203997, AF145492, KU251461, KU251460, KU251459, KU251458, KU251457	ON764804, KY980799, EU203966, EU203997, AF145492, KU251461, KU251460, KU251459, KU251458, KU251457	NA	ON764804, MT664744, MT449735, MT449734, MT449733, MT449732, MT449731, MT168616, MT168615, GQ491031	NA	ON764804, KY981012, EU203966, EU203997, GQ491031, JX033931, AB497583, EU203875	ON764804, KY981082, GQ491031, AB497585	NA	NA	ON764804, KY981219, KM384857, AF085507, GQ491031, JX244450, JX033943	NA
Schistocerca lineata	KY980775, KY980774, KY980760, KM504239, KM504238, KM504237, KM504236, KM504235, KM504234, KM504233	KY980810, KY980809, KY980796, KM504239, KM504238, KM504237, KM504236, KM504235, KM504234, KM504233	NA	NA	N/A	NA	N/A	N/A	NA	NA	N/A	NA
Schistocerca nitens	ON530890, KY980786,	ON530890, KY980822,	NA	NA	N/A	NA	ON530890, KY981034,	ON530890, KY981106,	NA	NA	ON530890, KY981242,	NA

Species	Gene										
	12S	***16S***	***ATP6***	***ATP8***	***COX1***	***COX2***	***CYTB***	***ND2***	***ND4***	***ND4L***	***ND5***	***ND6***
	KY980780, KY980765, KY980764, KY980758, KR904022, AY605934	KY980815, KY980801, KY980800, KY980794, AF155564, AY605934					KY981028, KY981014, KY981013, KY981007	KY981099, KY981084, KY981083, KY981077			KY981235, KY981221, KY981220	
Schistocerca pallens	KY980778, KY980762, KY980752, FJ393966, FJ393965, FJ393964, FJ393963, FJ393962, FJ393961, AY605946	KY980813, KY980798, KY980788, FJ393966, FJ393965, FJ393964, FJ393963, FJ393962, FJ393961, AY605946	NA	NA	N/A	NA	N/A	N/A	NA	NA	N/A	NA
Schistocerca piceifrons	N/A	OM650689, KY980814, FJ394021, FJ394020, FJ394019, FJ394018, FJ394017, FJ394016, FJ394015, FJ394014	NA	NA	N/A	NA	N/A	N/A	NA	NA	N/A	NA
Schistocerca shoshone	KY980757, KM504208, KM504207, KM504206, KM504203, KM504202, AY605941	KY980793, KM504208, KM504207, KM504206, KM504203, KM504202, AY605941	NA	NA	N/A	NA	N/A	N/A	NA	NA	N/A	NA
Sinopodisma tsinlingensis	N/A	N/A	NA	NA	N/A	NA	KX857635, KX781264, DQ366767, DQ366830	N/A	NA	NA	N/A	NA
Spathosternum prasiniferum	MN046219, KM588074, JQ247661, AY247167, AY247166	N/A	NA	NA	N/A	NA	MN046219, DQ366765, KM588074, DQ366828	N/A	NA	NA	N/A	NA
Sphingoderus carinatus	N/A	N/A	NA	NA	N/A	NA	N/A	N/A	NA	NA	KJ923393, KM494763, KM494746,	NA

Species	Gene											
	12S	***16S***	***ATP6***	***ATP8***	***COX1***	***COX2***	***CYTB***	***ND2***	***ND4***	***ND4L***	***ND5***	***ND6***
											KC208731, KC208711	
Sphingonotus azurescens	N/A	N/A	NA	NA	N/A	NA	N/A	N/A	NA	NA	JQ513141, EU266725, EU266724, EU266723, KM384873, JX244447, KM494765, KM494764, KM494735, KM494734	NA
Sphingonotus caerulans	N/A	N/A	NA	NA	N/A	NA	N/A	N/A	NA	NA	MW939473, MW939472, MW939471, MW939470, MW939469, JQ513143, JQ513142, EU266722, EU266721, JX244448	NA
Sphingonotus corsicus	N/A	N/A	NA	NA	N/A	NA	N/A	N/A	NA	NA	EU266720, EU266719, KM494749, JQ286628	NA
Sphingonotus finotianus	N/A	N/A	NA	NA	N/A	NA	N/A	N/A	NA	NA	JQ513147, EU266746, EU266745, KC208725	NA
Sphingonotus fuerteventurae	N/A	N/A	NA	NA	N/A	NA	N/A	N/A	NA	NA	JQ513148, EU266738, EU266737, EU266736, KM494751	NA
Sphingonotus fuscoirroratus	N/A	N/A	NA	NA	N/A	NA	N/A	N/A	NA	NA	KP201200, KP201199, KP201198, KJ923404, KJ923403, KJ923402, KJ923401, KJ923400, KJ923399, KJ923398	NA

Species	Gene											
	12S	*16S*	*ATP6*	*ATP8*	*COX1*	*COX2*	*CYTB*	*ND2*	*ND4*	*ND4L*	*ND5*	*ND6*
Sphingonotus lluciapomaresi	N/A	N/A	NA	NA	N/A	NA	N/A	N/A	NA	NA	KM494742, KM494741, KC208720, KC208719	NA
Sphingonotus pachecoi	N/A	N/A	NA	NA	N/A	NA	N/A	N/A	NA	NA	EU266735, EU266734, EU266733, EF151919, KM494769, KM494767	NA
Sphingonotus picteti	N/A	N/A	NA	NA	N/A	NA	N/A	N/A	NA	NA	EU266742, EU266741, EU266740, KM494771, KM494722	NA
Sphingonotus rubescens	N/A	N/A	NA	NA	N/A	NA	N/A	N/A	NA	NA	JQ513154, JQ513153, EU266716, EU266715, EU266714, EU266713, EU266712, EU266711, EU266710, KM494772	NA
Sphingonotus savignyi	N/A	N/A	NA	NA	N/A	NA	N/A	N/A	NA	NA	JQ513155, EU266729, EU266728, EU266727, KM494759, KM494747, KM494744, KM494724, KM494721, KM494719	NA
Sphingonotus sublaevis	N/A	N/A	NA	NA	N/A	NA	N/A	N/A	NA	NA	EU266732, EU266731, EU266730, KM494730, KM494729, KM494725	NA
Trilophidia annulata	N/A	N/A	NA	NA	PP228023, OK037584, MT325830, MT325829,	NA	MK903576, EU366107, EU366108, AY382875,	N/A	NA	NA	N/A	NA

Species	Gene											
	12S	*16S*	*ATP6*	*ATP8*	*COX1*	*COX2*	*CYTB*	*ND2*	*ND4*	*ND4L*	*ND5*	*ND6*
					MT325828, MT325827, MK903576, MT859410, MN907781, KP233803		AY157560, KP233803					
Trimerotropis andeana	N/A	N/A	NA	NA	N/A	NA	N/A	N/A	NA	NA	JQ286693, JQ286642, JQ286639, JQ286637	NA
Trimerotropis cyaneipennis	N/A	N/A	NA	NA	N/A	NA	N/A	N/A	NA	NA	JQ513161, KJ923417, KJ923416, KJ923415	NA
Trimerotropis maritima	N/A	N/A	NA	NA	N/A	NA	N/A	N/A	NA	NA	KJ923421, KJ923420, JQ286630, JQ286629	NA
Trimerotropis ochraceipennis	N/A	N/A	NA	NA	N/A	NA	N/A	N/A	NA	NA	JQ286691, JQ286689, JQ286688, JQ286683, JQ286682, JQ286681, JQ286680, JQ286679, JQ286678	NA
Trimerotropis pallidipennis	N/A	N/A	NA	NA	N/A	NA	N/A	N/A	NA	NA	JQ286693, JQ286691, JQ286689, JQ286688	NA
Trimerotropis saxatilis	N/A	N/A	NA	NA	N/A	NA	N/A	N/A	NA	NA	KJ923431, KJ923430, JQ286635, JQ286634, JQ286633	NA
Uvarovistia munzurensis	N/A	N/A	NA	NA	N/A	NA	N/A	MW261375, MW320700, MW320699, MW320698, MW320697, MW320696, MW320695, MW320694, MW320693	NA	NA	N/A	NA

Species	Gene										
	12S	***16S***	***ATP6***	***ATP8***	***COX1***	***COX2***	***CYTB***	***ND2***	***ND4***	***ND4L***	***ND5***	***ND6***
Uvarovistia satunini	N/A	N/A	NA	NA	N/A	NA	N/A	MW261383, MW261382, MW261381, MW261380, MW261379, MW261378, MW261377, MW261376, MW320699	NA	NA	N/A	NA
Uvarovistia zebra	N/A	N/A	NA	NA	N/A	NA	N/A	MW261387, MW261386, MW261385, MW261384, MW320700	NA	NA	N/A	NA
Xenocatantops brachycerus	N/A	N/A	NA	NA	MT916716, MW056475, MW056474, MW053534, KC542806	NA	MT916716, EU366109, EU366110, DQ366747, KC542806, DQ365906, DQ366807	MT916716, DQ092559, KC542806, JQ283267	NA	NA	N/A	NA
Xenocatantops humilis	N/A	N/A	NA	NA	OQ282992, OP373199, MT325832, MT325831	NA	OP373199, EU366111, EU366112, DQ366768, AY382873, DQ366831	OQ282992, OP373199, DQ092560, JQ283268	NA	NA	N/A	NA
Xenogryllus marmoratus	MK903577, MK761284, MK761283, MK033622, KY595484, KR904024	MK903577, MK761262, MK761261, MK033622, KY595510, KR903830, JQ06763	NA	NA	N/A	NA	N/A	N/A	NA	NA	N/A	NA
