# Supplementary material for: Expanding the mitochondrial genomic toolkit for Polyneoptera: New mitogenomes and evaluation of reduced marker sets for phylogeny and DNA barcoding
Source: Genet Mol Biol. 2026 Jul 24;49(3):e20250282. doi: 10.1590/1678-4685-GMB-2025-0282 (PMC13403772; doi:10.1590/1678-4685-GMB-2025-0282)
Supplement: Table S4 - [file 1415-4757-GMB-49-3-e20250282-s4.pdf]

Genetics and Molecular Biology

DOI: https://doi.org/10.1590/1678-4685-GMB-2025-0282

Supplementary Material to "Expanding the mitochondrial genomic toolkit for Polyneoptera: New mitogenomes and evaluation of reduced marker sets for phylogeny and DNA barcoding"

Table S4 - Nucleotide composition of mitochondrial genomes from Polyneoptera species, including AT content, GC-skew, and AT-skew calculated for the complete genome and for different genomic partitions (protein-coding genes, rRNAs, and tRNAs).

			Genome			PCG			rRNA			tRNA		
Order	Species	NCBI accession	AT	GC-skew	AT-skew	AT	GC-skew	AT-skew	AT	GC-skew	AT-skew	AT	GC-skew	AT-skew
	Acholotermes chirotus	NC_034135.1	65.89	-0.28	0.29	64.86	-0.06	-0.09	69.58	0.37	-0.33	68.10	0.14	0.02
	Aciculitermes aciculatus	NC_034089.1	68.49	-0.26	0.26	68.07	-0.02	-0.11	70.41	0.38	-0.32	68.76	0.13	0.03
	Aciculitermes maymyoensis	NC_034107.1	66.78	-0.26	0.26	65.86	-0.04	-0.11	70.66	0.35	-0.30	67.89	0.13	0.01
	Acidnotermes praus	NC_034122.1	65.79	-0.23	0.27	64.81	-0.03	-0.11	69.53	0.34	-0.33	67.54	0.15	0.03
	Agnathotermes crassinasus	NC_034025.1	66.37	-0.28	0.29	65.58	-0.04	-0.11	68.97	0.37	-0.33	68.38	0.13	0.03
	Allodontermes schultzei	NC_034079.1	63.35	-0.32	0.36	62.08	-0.07	-0.09	67.79	0.39	-0.38	66.28	0.13	0.03
	Alyscotermes kilimandjaricus	NC_034022.1	67.51	-0.25	0.26	66.75	-0.03	-0.10	70.97	0.35	-0.33	68.11	0.13	0.02
	Amalotermes phaeocephalus	NC_034099.1	66.74	-0.27	0.28	65.97	-0.04	-0.10	69.87	0.34	-0.34	67.86	0.15	0.02
	Amitermes capito	NC_034038.1	67.70	-0.26	0.27	67.16	-0.04	-0.10	69.54	0.34	-0.33	68.98	0.14	0.03
	Amitermes dentatus	NC_034075.1	67.00	-0.28	0.29	66.36	-0.04	-0.10	69.42	0.36	-0.35	68.18	0.13	0.04
	Amitermes meridionalis	NC_034062.1	67.12	-0.27	0.28	66.49	-0.05	-0.10	69.24	0.34	-0.33	68.60	0.14	0.03
	Amitermes obeuntis	NC_034124.1	67.86	-0.27	0.27	67.15	-0.04	-0.10	71.06	0.37	-0.34	68.78	0.14	0.03
	Anhangatermes macarthuri	NC_034092.1	67.15	-0.26	0.27	66.37	-0.03	-0.10	70.22	0.35	-0.31	68.36	0.13	0.02
	Anoplotermes janus	NC_034120.1	65.55	-0.25	0.29	64.20	-0.04	-0.10	71.54	0.34	-0.37	67.68	0.15	0.04
	Anoplotermes parvus	NC_034123.1	64.96	-0.22	0.28	63.74	-0.04	-0.10	69.33	0.32	-0.35	67.59	0.12	0.04
Blattodea	*Apilitermes longiceps*	NC_034031.1	68.16	-0.28	0.29	67.42	-0.04	-0.10	70.56	0.36	-0.33	70.11	0.15	0.03

Order	Species	NCBI accession	Genome			PCG			rRNA			tRNA		
			AT	GC-skew	AT-skew	AT	GC-skew	AT-skew	AT	GC-skew	AT-skew	AT	GC-skew	AT-skew
	Araujotermes parvellus	NC_034057.1	67.54	-0.26	0.27	66.80	-0.03	-0.10	70.36	0.35	-0.32	68.85	0.12	0.03
	Astalotermes murcus	NC_034132.1	67.56	-0.28	0.28	66.99	-0.04	-0.10	70.14	0.37	-0.35	68.01	0.13	0.02
	Ateuchotermes retifaciens	NC_034069.1	66.73	-0.31	0.31	66.00	-0.05	-0.09	69.20	0.41	-0.37	68.71	0.13	0.03
	Atlantitermes oculatissimus	NC_034148.1	67.56	-0.26	0.27	66.95	-0.02	-0.11	69.95	0.38	-0.33	68.57	0.14	0.01
	Atlantitermes snyderi	NC_034102.1	67.10	-0.26	0.27	66.41	-0.03	-0.10	69.54	0.37	-0.33	68.63	0.15	0.02
	Blaptica dubia	KT893459.1	70.83	-0.28	0.07	70.83	-0.03	-0.12	74.81	0.36	-0.08	73.69	0.19	0.01
	Blattella bisignata	NC_018549.1	74.03	-0.20	0.05	73.74	0.02	-0.15	76.05	0.34	-0.11	73.39	0.18	0.02
	Blattella germanica	NC_012901.1	74.22	-0.18	0.05	74.22	0.02	-0.15	76.40	0.33	-0.10	73.65	0.19	0.01
	Bulbitermes laticephalus	NC_034059.1	68.33	-0.28	0.27	67.84	-0.03	-0.10	70.67	0.38	-0.31	68.57	0.13	0.03
	Bulbitermes makhamensis	NC_034029.1	66.86	-0.28	0.28	65.90	-0.04	-0.10	70.65	0.37	-0.30	68.41	0.13	0.02
	Bulbitermes singaporiensis	NC_034147.1	65.57	-0.28	0.28	64.47	-0.04	-0.10	69.83	0.38	-0.32	67.49	0.14	0.03
	Cavitermes tuberosus	NC_034097.1	65.58	-0.26	0.28	64.67	-0.05	-0.10	69.01	0.35	-0.32	67.25	0.15	0.02
	Cephalotermes rectangularis	NC_034136.1	66.24	-0.28	0.29	65.27	-0.05	-0.10	69.68	0.35	-0.33	68.44	0.15	0.02
	Coatitermes kartaboensis	NC_034141.1	67.72	-0.26	0.26	67.15	-0.03	-0.10	70.09	0.36	-0.33	68.54	0.13	0.04
	Compositermes vindai	NC_034144.1	68.03	-0.25	0.28	67.46	-0.03	-0.10	70.42	0.36	-0.35	68.78	0.16	0.03
	Constrictotermes cyphergaster	NC_034044.1	66.04	-0.27	0.28	65.07	-0.05	-0.09	69.11	0.40	-0.32	68.72	0.15	0.03
	Coptotermes amanii	KU925200.1	62.95	-0.34	0.36	62.60	-0.07	-0.08	66.65	0.41	-0.38	65.55	0.11	0.03
	Coptotermes elisae	KU925201.1	63.51	-0.34	0.35	63.26	-0.06	-0.09	66.82	0.41	-0.38	65.36	0.11	0.03
	Coptotermes formosanus	NC_015800.1	64.57	-0.31	0.32	64.57	-0.05	-0.09	68.98	0.40	-0.37	65.89	0.12	0.04
	Coptotermes frenchi	KU925204.1	63.27	-0.33	0.35	62.92	-0.07	-0.08	66.82	0.39	-0.36	65.89	0.12	0.03
	Coptotermes gestroi	KU925205.1	63.36	-0.32	0.35	63.10	-0.05	-0.09	67.22	0.40	-0.37	65.33	0.13	0.03
	Coptotermes heimi	KU925206.1	63.01	-0.32	0.35	62.67	-0.06	-0.08	66.76	0.40	-0.37	65.60	0.13	0.03
	Coptotermes kalshoveni	KU925209.1	64.22	-0.35	0.35	64.06	-0.06	-0.09	67.03	0.41	-0.37	65.41	0.13	0.03
	Coptotermes lacteus	NC_018125.1	63.93	-0.32	0.33	63.18	-0.08	-0.08	66.85	0.40	-0.37	65.35	0.12	0.03

Order	Species	NCBI accession	Genome			PCG			rRNA			tRNA		
			AT	GC-skew	AT-skew	AT	GC-skew	AT-skew	AT	GC-skew	AT-skew	AT	GC-skew	AT-skew
	Coptotermes michaelseni	KU925212.1	63.61	-0.32	0.34	63.32	-0.06	-0.08	67.23	0.39	-0.35	65.80	0.12	0.02
	Coptotermes remotus	KU925213.1	63.87	-0.32	0.34	63.63	-0.06	-0.09	64.47	0.38	-0.36	65.68	0.14	0.02
	Coptotermes sepangensis	NC_030019.1	64.18	-0.32	0.34	63.90	-0.06	-0.09	64.49	0.37	-0.35	65.95	0.14	0.03
	Coptotermes sjoestedti	NC_030020.1	62.97	-0.30	0.35	61.94	-0.06	-0.09	66.22	0.39	-0.37	65.95	0.13	0.03
	Coptotermes suzhouensis	NC_037018.1	65.32	-0.32	0.33	64.58	-0.05	-0.09	68.93	0.41	-0.37	65.89	0.12	0.04
	Coptotermes testaceus	NC_028722.1	63.09	-0.32	0.35	62.01	-0.05	-0.09	66.34	0.39	-0.36	66.51	0.14	0.03
	Coptotermes travians	NC_030021.1	63.42	-0.32	0.35	62.45	-0.05	-0.09	66.83	0.40	-0.38	65.68	0.12	0.02
	Cornitermes cumulans	NC_034086.1	67.94	-0.26	0.26	67.13	-0.04	-0.10	71.42	0.35	-0.30	68.79	0.12	0.03
	Cornitermes pugnax	NC_034055.1	67.64	-0.25	0.26	66.72	-0.03	-0.11	71.57	0.36	-0.30	68.61	0.13	0.03
	Crenetermes albotarsalis	NC_034113.1	67.78	-0.27	0.27	67.10	-0.04	-0.10	70.35	0.34	-0.31	69.09	0.12	0.01
	Crepititermes verruculosus	NC_034041.1	66.75	-0.26	0.27	65.75	-0.04	-0.10	70.45	0.34	-0.32	68.68	0.12	0.02
	Cryptocercus changbaiensis	NC_059066.1	73.04	-0.25	0.24	72.38	-0.01	-0.08	75.73	0.35	-0.30	74.26	0.17	0.00
	Cryptocercus kyebangensis	NC_030191.1	73.96	-0.24	0.24	73.42	0.00	-0.09	76.49	0.34	-0.31	74.52	0.16	0.00
	Cryptocercus laojunensis	NC_059069.1	74.49	-0.25	0.23	74.11	-0.01	-0.08	76.84	0.33	-0.30	74.08	0.16	-0.01
	Cryptocercus meridianus	NC_037496.1	73.84	-0.25	0.22	73.28	-0.02	-0.09	76.17	0.36	-0.29	74.90	0.13	0.00
	Cryptocercus pudacuoensis	NC_059068.1	75.06	-0.21	0.21	74.80	-0.01	-0.09	77.09	0.32	-0.29	74.19	0.15	0.01
	Cryptocercus relictus	NC_018132.1	73.20	-0.23	0.23	72.55	-0.01	-0.08	75.71	0.34	-0.30	74.69	0.15	0.01
	Cryptocercus sanchaensis	NC_059064.1	73.95	-0.24	0.21	73.60	-0.02	-0.09	76.34	0.35	-0.28	73.22	0.14	0.00
	Cryptocercus tianbaensis	NC_059065.1	73.91	-0.22	0.21	73.47	-0.01	-0.09	76.47	0.34	-0.28	73.67	0.12	0.00
	Cryptocercus weixiensis	NC_059067.1	74.87	-0.23	0.22	74.67	-0.01	-0.09	76.34	0.35	-0.30	74.34	0.13	0.01
	Cryptotermes declivis	NC_045866.1	64.75	-0.26	0.27	63.77	-0.04	-0.13	67.84	0.34	-0.36	67.73	0.11	0.01
	Cryptotermes havilandi	NC_056118.1	65.65	-0.29	0.28	64.87	-0.03	-0.12	68.51	-0.35	0.36	67.45	0.15	-0.01
	Nitiditermes fulvus	NC_034033.1	67.79	-0.25	0.26	67.10	-0.04	-0.11	70.66	0.36	-0.30	68.67	0.17	0.01
	Cubitermes oblectatus	NC_034056.1	67.38	-0.26	0.27	66.73	-0.05	-0.10	69.60	0.36	-0.32	68.98	0.15	0.01

Order	Species	NCBI accession	Genome			PCG			rRNA			tRNA		
			AT	GC-skew	AT-skew	AT	GC-skew	AT-skew	AT	GC-skew	AT-skew	AT	GC-skew	AT-skew
	Polyspathotermes sulcifrons	NC_034109.1	68.10	-0.25	0.26	67.48	-0.05	-0.10	71.20	0.36	-0.32	68.78	0.17	0.00
	Isognathotermes ugandensis	NC_026113.1	67.06	-0.26	0.25	66.62	-0.04	-0.11	70.71	0.34	-0.31	70.23	0.12	0.02
	Cylindrotermes parvignathus	NC_034096.1	66.29	-0.30	0.31	65.32	-0.05	-0.09	69.66	0.38	-0.36	68.48	0.14	0.03
	Drepanotermes sp. SLC-2012	NC_018129.1	66.29	-0.27	0.26	65.44	-0.05	-0.10	69.53	0.35	-0.34	67.95	0.16	0.03
	Embiratermes brevinasus	NC_034101.1	65.90	-0.27	0.29	64.74	-0.04	-0.10	69.47	0.35	-0.33	69.25	0.13	0.02
	Embiratermes neotenicus	NC_034930.1	66.02	-0.28	0.28	65.08	-0.03	-0.10	69.67	0.40	-0.34	67.74	0.13	0.02
	Ephelotermes melachoma	NC_034019.1	65.41	-0.28	0.29	64.32	-0.05	-0.10	68.90	0.37	-0.33	68.39	0.11	0.03
	Ephelotermes taylori	NC_034149.1	65.83	-0.29	0.29	64.84	-0.06	-0.10	69.20	0.38	-0.33	68.18	0.12	0.03
	Euhamitermes hamatus	NC_034064.1	65.23	-0.24	0.30	64.12	-0.04	-0.09	69.22	0.33	-0.35	67.56	0.16	0.02
	Eupolyphaga sinensis	NC_014274.1	70.93	-0.25	0.12	70.93	0.01	-0.13	73.97	0.41	-0.20	74.35	0.18	0.03
	Foraminitermes rhinoceros	NC_034116.1	64.33	-0.29	0.33	63.15	-0.05	-0.08	68.42	0.34	-0.34	67.05	0.14	0.03
	Furculitermes cubitalis	NC_034131.1	64.65	-0.26	0.27	63.55	-0.04	-0.11	68.09	0.38	-0.32	67.79	0.13	0.03
	Furculitermes longilabius	NC_034128.1	64.50	-0.27	0.28	63.25	-0.04	-0.11	68.79	0.38	-0.33	67.90	0.13	0.02
	Furculitermes soyeri	NC_034082.1	64.29	-0.25	0.27	63.05	-0.04	-0.11	69.16	0.39	-0.33	67.03	0.14	0.02
	Furculitermes winifredae	NC_034063.1	64.07	-0.25	0.27	62.95	-0.04	-0.11	67.42	0.37	-0.33	67.41	0.14	0.02
	Geoscapheus dilatatus	MW600997.1	74.30	0.20	-0.06	74.20	-0.02	-0.14	77.05	0.36	-0.14	75.09	0.13	0.01
	Globitermes globosus	NC_034095.1	65.60	-0.30	0.30	64.50	-0.06	-0.09	69.21	0.38	-0.33	68.55	0.15	0.03
	Globitermes sulphureus	NC_034139.1	64.91	-0.29	0.30	63.79	-0.06	-0.10	68.08	0.38	-0.35	68.57	0.15	0.03
	Gromphadorhina portentosa	NC_030001.1	70.11	-0.23	0.03	69.26	-0.04	-0.14	72.64	0.33	-0.07	73.09	0.13	0.01
	Havilanditermes proatripennis	NC_034070.1	67.39	-0.28	0.27	66.72	-0.04	-0.10	69.69	0.42	-0.32	68.93	0.15	0.01
	Heterotermes cf. occiduus 3.12.2.AUS	NC_030028.1	64.70	-0.30	0.33	63.90	-0.05	-0.10	68.08	0.37	-0.37	65.75	0.11	0.03
	Heterotermes cf. occiduus 3.15.2AUS	NC_030029.1	64.15	-0.30	0.34	63.29	-0.05	-0.09	67.74	0.37	-0.37	65.33	0.11	0.03
	Heterotermes cf. paradoxus AUS103	NC_030023.1	65.02	-0.30	0.32	64.28	-0.05	-0.09	68.39	0.37	-0.36	65.60	0.11	0.03
	Heterotermes cf. paradoxus AUS121	NC_030024.1	64.39	-0.30	0.33	63.62	-0.05	-0.09	67.41	0.35	-0.36	65.62	0.11	0.03

Order	Species	NCBI accession	Genome			PCG			rRNA			tRNA		
			AT	GC-skew	AT-skew	AT	GC-skew	AT-skew	AT	GC-skew	AT-skew	AT	GC-skew	AT-skew
	Heterotermes cf. paradoxus AUS88	NC_030022.1	65.04	-0.30	0.32	64.32	-0.05	-0.09	68.35	0.37	-0.36	65.53	0.11	0.03
	Heterotermes crinitus	KU925226.1	60.87	-0.27	0.33	60.35	-0.06	-0.09	66.97	0.37	-0.35	64.78	0.11	0.02
	Heterotermes malabaricus	KU925227.1	62.61	-0.30	0.34	62.23	-0.05	-0.09	66.36	0.38	-0.37	65.51	0.14	0.02
	Heterotermes nr. tenuis COL.4PUERTO	NC_030027.1	63.09	-0.28	0.33	61.93	-0.05	-0.09	67.09	0.36	-0.36	65.91	0.10	0.03
	Heterotermes platycephalus	NC_030030.1	64.51	-0.30	0.33	63.67	-0.04	-0.10	68.12	0.37	-0.36	65.57	0.12	0.03
	Heterotermes sp. SLC-2012	NC_018127.1	64.33	-0.30	0.32	63.34	-0.05	-0.09	68.21	0.36	-0.37	66.20	0.11	0.03
	Heterotermes tenuior	NC_030031.1	64.42	-0.28	0.32	63.53	-0.06	-0.09	67.48	0.38	-0.34	66.64	0.13	0.03
	Heterotermes tenuis	KU925233.1	63.01	-0.28	0.33	62.57	-0.04	-0.09	67.65	0.36	-0.36	66.29	0.12	0.01
	Heterotermes vagus	KU925234.1	61.55	-0.30	0.34	61.11	-0.06	-0.09	66.84	0.37	-0.37	64.88	0.14	0.02
	Heterotermes validus	KU925235.1	64.40	-0.30	0.32	64.21	-0.05	-0.09	68.46	0.38	-0.36	65.87	0.12	0.03
	Hirtitermes hirtiventris	NC_034134.1	67.58	-0.31	0.29	66.76	-0.05	-0.09	70.52	0.38	-0.33	69.38	0.13	0.03
	Hospitalitermes hospitalis	NC_034074.1	64.84	-0.28	0.29	63.79	-0.05	-0.09	68.46	0.40	-0.32	67.25	0.14	0.02
	Hospitalitermes medioflavus	NC_036047.1	64.93	-0.28	0.29	63.91	-0.04	-0.10	68.40	0.40	-0.32	67.34	0.14	0.02
	Humutermes krishnai	NC_034129.1	65.96	-0.22	0.27	64.86	-0.05	-0.09	69.94	0.33	-0.34	68.20	0.16	0.02
	Hypotermes makhamensis	NC_034037.1	63.35	-0.31	0.36	62.33	-0.07	-0.08	66.61	0.38	-0.38	66.06	0.13	0.04
	Incisitermes minor	NC_037511.1	65.17	-0.23	0.26	64.65	-0.03	-0.12	66.98	0.32	-0.36	66.46	0.12	0.01
	Inquilinitermes inquilinus	NC_034118.1	66.22	-0.28	0.28	65.30	-0.05	-0.11	69.61	0.37	-0.32	68.02	0.16	0.01
	Jugositermes tuberculatus	NC_034083.1	65.43	-0.28	0.31	64.51	-0.05	-0.10	68.70	0.36	-0.36	67.52	0.16	0.03
	Labiotermes labralis	NC_034929.1	68.58	-0.25	0.24	67.86	-0.01	-0.13	71.39	0.35	-0.29	69.78	0.15	0.02
	Labritermes buttelreepeni	NC_034058.1	64.74	-0.28	0.32	63.63	-0.07	-0.08	68.67	0.35	-0.34	67.11	0.15	0.02
	Leucopitermes leucops	NC_034047.1	67.82	-0.26	0.25	67.27	-0.04	-0.11	70.31	0.35	-0.30	68.20	0.14	0.02
	Longustitermes manni	NC_034094.1	65.58	-0.28	0.31	64.41	-0.05	-0.10	70.07	0.38	-0.36	68.18	0.14	0.03
	Lophotermes septentrionalis	NC_034105.1	65.79	-0.30	0.30	64.80	-0.05	-0.10	68.97	0.38	-0.34	68.65	0.11	0.03
	Macrognathotermes errator	NC_018130.1	66.03	-0.28	0.27	65.07	-0.04	-0.11	69.31	0.37	-0.34	68.45	0.13	0.03

Order	Species	NCBI accession	Genome			PCG			rRNA			tRNA		
			AT	GC-skew	AT-skew	AT	GC-skew	AT-skew	AT	GC-skew	AT-skew	AT	GC-skew	AT-skew
	Macrotermes annandalei	NC_034078.1	65.93	-0.32	0.35	64.98	-0.07	-0.08	68.98	0.37	-0.39	68.49	0.15	0.03
	Macrotermes barneyi	NC_018599.1	65.53	-0.33	0.34	65.53	-0.06	-0.09	69.69	0.39	-0.41	68.98	0.14	0.03
	Macrotermes carbonarius	NC_034046.1	65.53	-0.33	0.35	64.34	-0.06	-0.09	69.48	0.38	-0.40	68.65	0.13	0.03
	Macrotermes falciger	NC_034050.1	65.81	-0.32	0.34	64.97	-0.06	-0.08	68.51	0.36	-0.39	68.08	0.14	0.02
	Macrotermes gilvus	NC_034110.1	65.78	-0.33	0.35	64.86	-0.06	-0.08	68.80	0.39	-0.40	68.20	0.15	0.03
	Macrotermes malaccensis	NC_034030.1	65.88	-0.32	0.34	64.77	-0.07	-0.09	70.38	0.37	-0.39	68.53	0.14	0.04
	Macrotermes muelleri	NC_034127.1	64.34	-0.31	0.36	63.09	-0.06	-0.08	68.10	0.37	-0.41	68.11	0.15	0.03
	Macrotermes natalensis	NC_025522.1	64.39	-0.33	0.35	64.39	-0.06	-0.08	67.83	0.38	-0.41	67.94	0.13	0.02
	Macrotermes subhyalinus	NC_018128.1	65.16	-0.33	0.34	64.12	-0.06	-0.09	68.35	0.38	-0.40	68.41	0.13	0.02
	Macrotermes vitrialatus	NC_034054.1	65.05	-0.32	0.35	63.92	-0.05	-0.09	68.28	0.40	-0.40	68.66	0.17	0.02
	Macrotermes yunnanensis	KU900578.1	65.40	-0.33	0.34	65.40	-0.06	-0.08	69.15	0.38	-0.41	68.53	0.15	0.03
	Mastotermes darwiniensis	NC_018120.1	67.73	-0.25	0.17	67.10	-0.02	-0.13	69.67	0.34	-0.23	69.78	0.15	0.00
	Microcerotermes baluchistanicus	NC_034065.1	65.81	-0.26	0.28	64.77	-0.03	-0.10	69.21	0.35	-0.31	68.46	0.12	0.03
	Microcerotermes crassus	NC_034036.1	65.55	-0.26	0.28	64.45	-0.04	-0.10	69.45	0.36	-0.31	68.03	0.14	0.01
	Microcerotermes fuscotibialis	NC_034067.1	64.11	-0.27	0.30	62.83	-0.05	-0.10	67.69	0.39	-0.34	68.30	0.13	0.03
	Microcerotermes havilandi	NC_034084.1	65.80	-0.25	0.27	64.70	-0.04	-0.10	69.51	0.36	-0.31	68.47	0.15	0.02
	Microcerotermes nervosus	NC_034104.1	66.26	-0.26	0.28	65.29	-0.04	-0.10	69.42	0.36	-0.32	68.76	0.15	0.02
	Microcerotermes newmani	NC_034021.1	66.26	-0.25	0.27	65.29	-0.03	-0.10	69.38	0.36	-0.31	68.83	0.16	0.02
	Microcerotermes parvus	NC_026114.1	63.67	-0.27	0.28	63.07	-0.05	-0.10	67.82	0.39	-0.33	67.99	0.13	0.03
	Microcerotermes progrediens	NC_034133.1	64.87	-0.27	0.29	63.73	-0.06	-0.10	68.97	0.37	-0.34	67.81	0.13	0.02
	Microcerotermes serrula	NC_034142.1	66.98	-0.26	0.27	66.01	-0.04	-0.11	70.65	0.35	-0.31	68.72	0.15	0.01
	Microhodotermes viator	NC_018122.1	66.91	-0.29	0.33	66.12	-0.07	-0.08	70.11	0.33	-0.36	68.22	0.13	0.04
	Microtermes obesi	NC_034072.1	63.75	-0.32	0.34	62.60	-0.07	-0.09	67.06	0.38	-0.38	67.43	0.14	0.01
	Mirocapritermes connectens	NC_034085.1	67.53	-0.27	0.29	66.68	-0.04	-0.10	70.81	0.34	-0.34	68.99	0.13	0.03

Order	Species	NCBI accession	Genome			PCG			rRNA			tRNA		
			AT	GC-skew	AT-skew	AT	GC-skew	AT-skew	AT	GC-skew	AT-skew	AT	GC-skew	AT-skew
	Nasutitermes arborum	NC_034108.1	65.79	-0.29	0.30	64.81	-0.05	-0.10	69.19	0.37	-0.34	67.98	0.13	0.03
	Nasutitermes banksi	NC_034026.1	64.72	-0.27	0.29	63.52	-0.06	-0.10	68.82	0.38	-0.32	67.58	0.15	0.02
	Nasutitermes corniger	NC_026115.1	63.91	-0.28	0.27	63.31	-0.06	-0.10	68.73	0.37	-0.32	68.25	0.13	0.05
	Nasutitermes diabolus	NC_034020.1	66.01	-0.28	0.29	65.10	-0.04	-0.10	69.30	0.37	-0.33	67.96	0.13	0.03
	Nasutitermes exitiosus	NC_034115.1	64.84	-0.29	0.30	63.64	-0.06	-0.10	68.78	0.38	-0.34	67.87	0.14	0.02
	Nasutitermes graveolus	NC_034040.1	64.99	-0.30	0.30	63.65	-0.06	-0.09	70.26	0.41	-0.35	68.11	0.13	0.02
	Nasutitermes latifrons	NC_034117.1	64.64	-0.29	0.30	63.36	-0.06	-0.10	69.41	0.40	-0.35	67.83	0.14	0.02
	Nasutitermes longipennis	NC_034060.1	65.28	-0.30	0.30	64.16	-0.06	-0.09	69.18	0.39	-0.34	67.78	0.14	0.02
	Nasutitermes longirostris	NC_034023.1	67.64	-0.28	0.27	66.88	-0.03	-0.10	70.56	0.38	-0.29	68.97	0.14	0.03
	Nasutitermes lujae	NC_034042.1	65.49	-0.28	0.28	64.40	-0.04	-0.10	69.21	0.37	-0.31	68.00	0.14	0.04
	Nasutitermes macrocephalus	NC_034146.1	64.35	-0.28	0.30	63.02	-0.06	-0.10	68.74	0.39	-0.34	67.74	0.13	0.02
	Nasutitermes matangensis	NC_034034.1	63.74	-0.28	0.30	62.32	-0.05	-0.10	68.60	0.39	-0.33	67.16	0.13	0.04
	Nasutitermes neoparvus	NC_034080.1	67.41	-0.29	0.28	66.79	-0.03	-0.10	69.88	0.38	-0.31	68.35	0.13	0.03
	Nasutitermes octopilis	NC_034045.1	64.25	-0.28	0.29	62.91	-0.05	-0.10	68.90	0.38	-0.33	67.31	0.14	0.03
	Nasutitermes similis	NC_034093.1	64.31	-0.27	0.29	62.98	-0.05	-0.11	68.57	0.38	-0.32	67.90	0.14	0.03
	Nasutitermes triodiae	NC_018131.1	65.47	-0.29	0.28	64.35	-0.05	-0.11	69.17	0.38	-0.34	68.54	0.15	0.02
	Nauphoeta cinerea	NC_035052.1	71.24	-0.21	0.04	70.73	-0.03	-0.12	73.50	0.35	-0.08	72.03	0.13	0.03
	Neocapritermes angusticeps	NC_034053.1	68.09	-0.25	0.27	67.34	-0.04	-0.10	70.99	0.34	-0.34	69.37	0.14	0.02
	Neocapritermes taracua	NC_026116.1	66.69	-0.28	0.28	66.45	-0.04	-0.10	70.76	0.36	-0.34	68.43	0.14	0.02
	Neostylopyga rhombifolia	NC_034842.1	74.58	-0.19	0.13	74.06	0.02	-0.11	77.07	0.34	-0.25	74.95	0.15	0.02
	Neotermes insularis	NC_018124.1	67.71	-0.27	0.26	67.34	-0.04	-0.11	69.57	0.36	-0.34	67.78	0.12	-0.01
	Neotermes koshunensis	NC_046741.1	68.06	-0.24	0.26	67.72	-0.02	-0.10	69.98	0.31	-0.34	67.91	0.16	-0.02
	Noditermes cristifrons	NC_034145.1	66.32	-0.24	0.26	65.24	-0.05	-0.09	69.95	0.35	-0.30	68.93	0.16	0.02
	Occasitermes occasus	NC_034088.1	66.34	-0.27	0.28	65.32	-0.04	-0.10	70.84	0.38	-0.35	68.05	0.13	0.03

Order	Species	NCBI accession	Genome			PCG			rRNA			tRNA		
			AT	GC-skew	AT-skew	AT	GC-skew	AT-skew	AT	GC-skew	AT-skew	AT	GC-skew	AT-skew
	Odontotermes hainanensis	NC_034028.1	63.41	-0.31	0.36	62.25	-0.08	-0.07	67.05	0.38	-0.39	66.58	0.12	0.04
	Odontotermes javanicus	NC_034106.1	62.96	-0.30	0.36	61.81	-0.08	-0.08	66.33	0.37	-0.39	66.46	0.13	0.03
	Odontotermes longignathus	NC_034130.1	63.55	-0.31	0.36	62.61	-0.08	-0.08	66.41	0.37	-0.39	66.31	0.14	0.03
	Odontotermes mathuri	NC_034035.1	63.92	-0.31	0.36	62.94	-0.09	-0.08	67.15	0.37	-0.38	66.51	0.13	0.04
	Odontotermes minutus	NC_034061.1	63.80	-0.31	0.36	62.83	-0.08	-0.08	66.82	0.38	-0.39	66.58	0.13	0.04
	Odontotermes obesus	NC_034027.1	63.26	-0.30	0.36	62.18	-0.07	-0.08	66.36	0.38	-0.38	66.76	0.13	0.03
	Ophiotermes grandilabius	NC_034076.1	63.87	-0.26	0.28	62.50	-0.04	-0.11	68.74	0.38	-0.33	67.02	0.12	0.02
	Ophiotermes mirandus	NC_034068.1	63.48	-0.25	0.28	62.05	-0.04	-0.11	68.05	0.37	-0.32	67.27	0.12	0.02
	Opisthoplatia orientalis	KT893460.1	74.44	-0.24	0.05	74.44	0.00	-0.12	78.05	0.30	-0.07	74.98	0.15	0.01
	Oriensubulitermes inanis	NC_034087.1	68.01	-0.27	0.26	67.29	-0.02	-0.11	71.60	0.39	-0.32	68.41	0.13	0.02
	Orientotermes emersoni	NC_034048.1	70.25	-0.25	0.22	69.80	-0.02	-0.11	72.64	0.34	-0.30	70.05	0.12	0.03
	Orthotermes depressifrons	NC_034125.1	66.15	-0.26	0.28	65.05	-0.05	-0.10	69.92	0.33	-0.33	68.81	0.14	0.03
	Orthotermes mansuetus	NC_034100.1	66.13	-0.26	0.28	65.10	-0.06	-0.09	69.50	0.33	-0.32	68.88	0.16	0.01
	Panchlora nivea	NC_030002.1	73.57	-0.19	0.06	72.86	-0.01	-0.13	77.61	0.30	-0.14	73.21	0.14	0.01
	Patawatermes nigripunctatus	NC_034032.1	67.30	-0.19	0.25	66.68	-0.04	-0.11	70.04	0.29	-0.33	67.91	0.16	0.02
	Patawatermes turricola	NC_034137.1	66.87	-0.21	0.26	66.01	-0.04	-0.10	70.17	0.30	-0.34	68.45	0.15	0.03
	Pericapritermes dolichocephalus	NC_034112.1	68.19	-0.24	0.27	67.42	-0.02	-0.12	71.78	0.32	-0.35	69.07	0.14	0.03
	Pericapritermes nitobei	NC_039398.1	67.29	-0.25	0.28	66.36	-0.04	-0.12	70.42	0.33	-0.33	69.72	0.13	0.01
	Periplaneta americana	NC_016956.1	73.00	-0.21	0.13	73.00	0.01	-0.12	76.06	0.32	-0.23	75.80	0.17	0.02
	Periplaneta australasiae	NC_034841.1	74.54	-0.19	0.12	74.08	0.04	-0.13	76.37	0.35	-0.23	75.40	0.16	0.03
	Periplaneta brunnea	NC_039940.1	74.50	-0.19	0.13	73.87	0.03	-0.11	77.45	0.33	-0.22	75.07	0.16	0.02
	Periplaneta fuliginosa	NC_006076.1	74.59	-0.17	0.12	74.59	0.03	-0.12	77.49	0.31	-0.22	75.32	0.15	0.04
	Planicapritermes planiceps	NC_034090.1	67.40	-0.27	0.28	66.75	-0.04	-0.10	69.81	0.36	-0.34	68.62	0.15	0.03
	Polyphaga plancyi	NC_049567.1	72.93	-0.29	0.16	72.39	0.00	-0.12	75.71	0.39	-0.23	73.01	0.16	0.01

Order	Species	NCBI accession	Genome			PCG			rRNA			tRNA		
			AT	GC-skew	AT-skew	AT	GC-skew	AT-skew	AT	GC-skew	AT-skew	AT	GC-skew	AT-skew
	Porotermes adamsoni	NC_018121.1	66.38	-0.28	0.28	65.68	-0.03	-0.12	68.54	0.32	-0.35	68.62	0.19	0.04
	Postsubulitermes parviconstrictus	NC_034114.1	67.59	-0.29	0.28	66.98	-0.04	-0.10	70.41	0.37	-0.32	68.01	0.11	0.03
	Proboscitermes tubuliferus	NC_034071.1	66.13	-0.25	0.28	65.08	-0.04	-0.10	69.49	0.34	-0.32	68.93	0.16	0.02
	Procapritermes martyni	NC_034119.1	69.69	-0.24	0.25	68.94	-0.03	-0.10	73.31	0.32	-0.31	69.89	0.12	0.04
	Procubitermes undulans	NC_034138.1	67.21	-0.24	0.25	66.31	-0.04	-0.11	70.50	0.32	-0.29	69.06	0.13	0.03
	Prohamitermes mirabilis	NC_034039.1	66.44	-0.30	0.30	65.53	-0.05	-0.09	69.64	0.37	-0.34	68.53	0.15	0.03
	Promirotermes pygmaeus	NC_034081.1	64.90	-0.29	0.30	63.72	-0.05	-0.10	69.03	0.39	-0.34	67.59	0.15	0.01
	Protermes prorepens	NC_034126.1	64.71	-0.31	0.35	63.89	-0.07	-0.08	66.97	0.36	-0.37	67.50	0.12	0.03
	Pseudacanthotermes militaris	NC_034077.1	65.26	-0.33	0.34	64.04	-0.06	-0.09	69.48	0.41	-0.39	68.01	0.12	0.04
	Pseudacanthotermes spiniger	NC_034024.1	66.38	-0.34	0.34	65.50	-0.06	-0.09	69.82	0.40	-0.40	68.24	0.13	0.05
	Reticulitermes aculabialis	NC_026695.1	64.52	-0.29	0.30	64.52	-0.04	-0.09	67.92	0.36	-0.35	67.54	0.12	0.04
	Reticulitermes chinensis	NC_025567.1	64.09	-0.29	0.30	64.09	-0.04	-0.10	68.09	0.35	-0.35	68.27	0.08	0.05
	Reticulitermes flaviceps	NC_031162.1	65.79	-0.28	0.29	64.90	-0.04	-0.10	68.36	0.35	-0.35	68.89	0.09	0.04
	Reticulitermes flavipes	NC_009498.1	64.75	-0.29	0.30	64.75	-0.05	-0.09	68.52	0.34	-0.35	67.92	0.12	0.04
	Reticulitermes grassei	KU925237.1	64.62	-0.27	0.30	64.23	-0.03	-0.11	68.31	0.34	-0.34	67.58	0.11	0.05
	Reticulitermes hageni	NC_009501.1	63.82	-0.29	0.30	63.82	-0.04	-0.10	68.98	0.34	-0.34	68.03	0.12	0.04
	Reticulitermes labralis	NC_030262.1	64.83	-0.28	0.30	63.74	-0.04	-0.10	68.49	0.35	-0.34	68.02	0.10	0.04
	Reticulitermes leptomandibularis	NC_042419.1	65.13	-0.29	0.31	64.32	-0.04	-0.09	67.92	0.36	-0.35	67.40	0.10	0.05
	Reticulitermes lucifugus	NC_045240.1	64.65	-0.27	0.29	64.24	-0.03	-0.10	67.67	0.33	-0.36	67.76	0.12	0.04
	Reticulitermes nelsonae	KU925238.1	64.35	-0.29	0.31	63.86	-0.05	-0.10	64.58	0.30	-0.31	68.01	0.12	0.04
	Reticulitermes ovatilabrum	NC_053728.1	65.29	-0.28	0.30	64.37	-0.04	-0.10	68.02	0.36	-0.33	68.38	0.11	0.03
	Reticulitermes santonensis	NC_009499.1	64.57	-0.29	0.30	64.57	-0.04	-0.10	68.34	0.35	-0.35	67.98	0.12	0.04
	Reticulitermes tibialis	NC_045231.1	64.40	-0.27	0.31	63.98	-0.03	-0.10	67.64	0.34	-0.34	67.51	0.12	0.04
	Reticulitermes virginicus	NC_009500.1	64.77	-0.29	0.30	64.77	-0.05	-0.09	68.11	0.34	-0.34	67.71	0.11	0.04

Order	Species	NCBI accession	Genome			PCG			rRNA			tRNA		
			AT	GC-skew	AT-skew	AT	GC-skew	AT-skew	AT	GC-skew	AT-skew	AT	GC-skew	AT-skew
	Rhinotermes hispidus	KU925240.1	65.15	-0.30	0.33	64.82	-0.07	-0.10	70.03	0.36	-0.34	67.55	0.11	0.05
	Roisinitermes ebogoensis	NC_040119.1	66.39	-0.30	0.24	66.10	-0.04	-0.11	66.78	0.38	-0.32	68.05	0.15	-0.01
	Rubeotermes jheringi	NC_034111.1	67.67	-0.24	0.26	67.08	-0.03	-0.12	69.96	0.32	-0.34	68.67	0.14	0.03
	Ruptitermes arboreus	NC_034140.1	65.65	-0.27	0.30	64.77	-0.04	-0.10	69.63	0.32	-0.37	66.51	0.15	0.02
	Schedorhinotermes breinli	NC_018126.1	65.29	-0.31	0.33	63.95	-0.07	-0.09	71.01	0.36	-0.36	67.09	0.13	0.03
	Periplaneta lateralis	NC_030003.1	73.74	-0.21	0.13	73.06	0.01	-0.12	76.49	0.34	-0.23	74.93	0.17	0.03
	Silvestritermes heyeri	NC_034066.1	68.14	-0.25	0.27	67.51	-0.03	-0.10	71.13	0.34	-0.32	68.37	0.13	0.04
	Sphaerotermes sphaerothorax	NC_034103.1	65.70	-0.28	0.28	64.90	-0.06	-0.11	68.32	0.39	-0.31	67.79	0.13	0.02
	Spinitermes trispinosus	NC_034091.1	64.53	-0.27	0.29	63.42	-0.05	-0.10	68.63	0.40	-0.35	66.69	0.14	0.02
	Termes comis	NC_034121.1	65.65	-0.28	0.30	64.60	-0.05	-0.10	69.28	0.38	-0.34	68.10	0.11	0.03
	Termes fatalis	NC_034049.1	65.56	-0.28	0.29	64.48	-0.06	-0.09	69.54	0.36	-0.33	67.70	0.15	0.02
	Termes hospes	NC_026117.1	64.83	-0.29	0.28	64.41	-0.05	-0.10	68.71	0.36	-0.33	67.97	0.13	0.03
	Termes rostratus	NC_034043.1	65.64	-0.29	0.30	64.60	-0.05	-0.09	69.01	0.36	-0.34	68.23	0.11	0.03
	Thoracotermes macrothorax	NC_034143.1	67.49	-0.27	0.27	66.65	-0.05	-0.10	70.35	0.38	-0.32	69.47	0.16	0.02
	Trichotermes ducis	NC_034073.1	66.24	-0.27	0.30	65.43	-0.04	-0.10	68.96	0.36	-0.36	68.22	0.14	0.03
	Tuberculitermes bycanistes	NC_034052.1	66.04	-0.28	0.28	65.12	-0.05	-0.10	68.93	0.38	-0.34	68.96	0.12	0.03
	Tumulitermes pastinator	NC_034098.1	65.25	-0.29	0.30	64.06	-0.06	-0.10	69.16	0.38	-0.33	68.32	0.13	0.03
	Tumulitermes recalvus	NC_034051.1	66.22	-0.29	0.29	65.30	-0.06	-0.09	69.21	0.38	-0.34	68.62	0.13	0.03
	Zootermopsis angusticollis	NC_018123.1	68.82	-0.30	0.33	68.03	-0.01	-0.11	71.72	0.34	-0.39	70.75	0.15	0.04
	Zootermopsis nevadensis	NC_024658.1	68.04	-0.29	0.32	68.04	0.00	-0.12	71.64	0.33	-0.39	71.29	0.15	0.04
	Average		66.49	-0.27	0.28	65.73	-0.04	-0.10	69.80	0.36	-0.33	68.54	0.14	0.03
	Standard deviation (SD)		2.92	0.05	0.07	3.07	0.02	0.01	2.65	0.05	0.07	2.31	0.02	0.01
	Coefficient of variation (CV)		0.04	-0.17	0.23	0.05	-0.49	-0.13	0.04	0.15	-0.22	0.03	0.13	0.44
Mantodea	*Amantis nawai*	NC_037203.1	75.38	-0.18	0.07	74.50	0.01	-0.13	79.13	0.34	-0.10	75.74	0.16	0.02

Order	Species	NCBI accession	Genome			PCG			rRNA			tRNA		
			AT	GC-skew	AT-skew	AT	GC-skew	AT-skew	AT	GC-skew	AT-skew	AT	GC-skew	AT-skew
	Amorphoscelis hainana	NC_057070.1	72.56	-0.27	0.02	71.09	-0.05	-0.14	75.89	0.40	-0.07	75.72	0.12	-0.01
	Anaxarcha zhengi	NC_030268.1	77.79	-0.16	0.07	77.00	0.04	-0.12	80.24	0.32	-0.10	77.35	0.16	0.01
	Arria pallida	NC_051892.1	78.22	-0.20	0.03	77.20	-0.01	-0.13	80.58	0.35	-0.06	79.19	0.15	0.01
	Creobroter gemmatus	NC_030267.1	75.99	-0.21	0.04	75.48	0.01	-0.13	77.39	0.35	-0.05	75.22	0.18	0.01
	Creobroter jiangxiensis	NC_037234.1	75.87	-0.20	0.04	75.33	0.01	-0.13	77.31	0.35	-0.05	75.30	0.15	0.01
	Eomantis yunnanensis	NC_037208.1	73.73	-0.23	0.03	72.95	-0.02	-0.15	75.00	0.39	-0.08	75.68	0.11	0.01
	Titanodula formosana	NC_029326.1	75.77	-0.20	0.05	75.26	-0.01	-0.13	78.29	0.40	-0.07	76.73	0.16	0.00
	Hierodula membranacea	NC_048984.1	74.84	-0.23	0.05	74.17	0.01	-0.14	77.96	0.39	-0.08	75.98	0.17	0.01
	Hierodula patellifera	NC_034283.1	74.45	-0.24	0.05	74.22	-0.02	-0.14	76.62	0.42	-0.11	76.41	0.16	0.02
	Humbertiella nada	NC_030264.1	70.12	-0.26	0.01	68.93	-0.05	-0.15	73.07	0.41	-0.01	73.12	0.15	-0.01
	Leptomantella albella	NC_024028.1	73.66	-0.21	0.02	72.36	-0.04	-0.15	76.81	0.38	-0.02	74.97	0.15	0.01
	Mantis religiosa	NC_030265.1	76.70	-0.19	0.03	76.21	0.02	-0.15	77.91	0.36	-0.06	75.84	0.15	0.01
	Paratoxodera polyacantha	NC_037697.1	74.81	-0.19	0.04	73.87	-0.01	-0.13	77.01	0.34	-0.04	76.82	0.16	0.01
	Pliacanthopus bimaculatus	NC_051490.1	73.12	-0.23	0.03	71.64	-0.01	-0.17	75.83	0.43	-0.06	75.93	0.16	0.03
	Psychomantis borneensis	NC_045876.1	75.52	-0.21	0.04	74.51	-0.01	-0.14	78.23	0.39	-0.06	76.68	0.14	0.03
	Rhombodera brachynota	NC_034282.1	74.80	-0.20	0.04	75.19	0.00	-0.14	77.57	0.39	-0.09	76.18	0.16	0.02
	Hierodula longa	NC_051489.1	76.15	-0.22	0.05	75.57	-0.01	-0.13	77.88	0.39	-0.07	77.24	0.17	0.00
	Rhombodera valida	NC_034284.1	74.82	-0.23	0.07	74.63	-0.01	-0.13	77.74	0.40	-0.09	77.51	0.14	0.01
	Sceptuchus simplex	NC_037206.1	73.37	-0.23	0.05	72.56	-0.03	-0.16	75.46	0.42	-0.08	75.15	0.16	0.01
	Schizocephala bicornis	NC_037207.1	74.94	-0.20	0.02	74.46	0.00	-0.14	77.47	0.39	-0.04	75.53	0.21	0.02
	Sibylla pretiosa	NC_037235.1	75.50	-0.22	0.03	74.39	0.03	-0.16	77.55	0.43	-0.08	76.45	0.16	0.01
	Sphodromantis lineola	NC_037204.1	74.68	-0.22	0.04	73.85	0.01	-0.14	77.67	0.39	-0.08	75.39	0.17	0.00
	Tamolanica tamolana	NC_007702.1	75.27	-0.24	0.06	74.79	0.00	-0.14	76.99	0.42	-0.11	76.54	0.13	0.01
	Tenodera sinensis	NC_030266.1	75.49	-0.22	0.06	74.78	0.02	-0.13	77.77	0.37	-0.10	75.54	0.15	0.02

Order	Species	NCBI accession	Genome			PCG			rRNA			tRNA		
			AT	GC-skew	AT-skew	AT	GC-skew	AT-skew	AT	GC-skew	AT-skew	AT	GC-skew	AT-skew
	Tropidomantis tenera	NC_037205.1	74.27	-0.22	0.04	73.18	0.00	-0.14	76.01	0.40	-0.06	76.13	0.10	0.01
	Average		74.92	-0.22	0.04	74.16	0.00	-0.14	77.28	0.39	-0.07	76.09	0.15	0.01
	Standard deviation (SD)		1.62	0.02	0.02	1.81	0.02	0.01	1.55	0.03	0.02	1.10	0.02	0.01
	Coefficient of variation (CV)		0.02	-0.11	0.39	0.02	-4.40	-0.09	0.02	0.08	-0.35	0.01	0.14	1.04
	Abracris flavolineata	BK068635	74.68	-0.18	0.15	73.53	-0.03	-0.13	76.34	0.24	-0.15	73.11	0.11	0.02
	Acosmetura nigrogeniculata	NC_045212.1	71.19	-0.27	0.02	71.13	-0.02	-0.15	74.62	0.39	-0.03	74.56	0.15	0.02
	Acrida cinerea	NC_014887.1	76.07	-0.16	0.17	75.33	0.00	-0.12	76.31	0.27	-0.18	74.37	0.13	0.01
	Acrida willemsei	NC_011303.1	76.22	-0.16	0.17	75.42	0.00	-0.12	76.97	0.26	-0.17	74.32	0.11	0.02
	Aiolopus thalassinus	NC_034674.1	75.25	-0.16	0.18	74.46	-0.02	-0.11	77.16	0.25	-0.19	72.85	0.12	0.02
	Alulatettix yunnanensis	NC_018542.1	75.24	-0.21	0.19	74.52	-0.01	-0.11	77.95	0.31	-0.26	75.19	0.15	0.01
	Anabropsis carli	NC_035420.1	75.39	-0.20	0.04	74.30	0.03	-0.14	76.61	0.33	-0.07	76.89	0.15	0.00
	Anabropsis carnarius	NC_035552.1	74.76	-0.24	0.05	73.27	-0.01	-0.13	76.42	0.34	-0.08	76.54	0.17	0.01
	Anabropsis crenatis	NC_035553.1	75.02	-0.23	0.05	73.44	0.00	-0.13	77.30	0.32	-0.08	77.06	0.16	0.00
	Anabrus simplex	NC_009967.1	69.44	-0.28	0.03	67.68	-0.05	-0.17	71.39	0.35	-0.05	73.05	0.13	0.01
	Anapodisma miramae	NC_052715.1	76.64	-0.15	0.13	76.32	0.00	-0.14	77.31	0.25	-0.15	75.00	0.12	0.02
	Angaracris barabensis	NC_025558.1	75.49	-0.17	0.16	74.53	-0.01	-0.11	76.08	0.25	-0.17	73.87	0.14	0.01
	Angaracris rhodopa	NC_025946.1	75.38	-0.17	0.16	74.38	-0.01	-0.11	75.99	0.24	-0.17	74.15	0.13	0.01
	Anterastes babadaghi	NC_046894.1	70.84	-0.25	0.03	70.03	-0.03	-0.16	72.02	0.35	-0.07	73.23	0.15	0.01
	Apalacris nigrogeniculata	NC_046527.1	73.99	-0.18	0.15	73.19	-0.02	-0.12	74.95	0.27	-0.16	72.39	0.08	0.04
	Arcyptera coreana	NC_013805.1	76.34	-0.13	0.13	75.85	0.00	-0.13	76.39	0.21	-0.14	73.26	0.10	0.03
	Arcyptera meridionalis	NC_039962.1	76.38	-0.15	0.12	75.82	0.00	-0.13	75.95	0.23	-0.13	73.78	0.10	0.04
	Asiotmethis jubatus	NC_025904.1	72.28	-0.18	0.14	71.99	-0.03	-0.14	71.57	0.24	-0.16	70.18	0.10	0.04
	Asiotmethis zacharjini	NC_020328.1	72.25	-0.18	0.15	71.74	-0.01	-0.14	72.27	0.23	-0.17	70.22	0.09	0.04
Orthoptera	*Atractomorpha psittacina*	NC_046552.1	74.38	-0.17	0.17	73.71	0.00	-0.13	75.27	0.26	-0.22	74.06	0.12	0.03

Order	Species	NCBI accession	Genome			PCG			rRNA			tRNA		
			AT	GC-skew	AT-skew	AT	GC-skew	AT-skew	AT	GC-skew	AT-skew	AT	GC-skew	AT-skew
	Atractomorpha sinensis	NC_011824.1	74.29	-0.18	0.16	73.57	0.01	-0.14	75.68	0.27	-0.21	73.60	0.13	0.02
	Bryodema dolichoptera	NC_046543.1	75.48	-0.17	0.16	74.63	-0.01	-0.11	76.04	0.23	-0.16	73.96	0.14	0.01
	Bryodema kozlovi	NC_052731.1	75.40	-0.19	0.16	74.34	-0.02	-0.11	76.50	0.25	-0.17	74.08	0.14	0.01
	Bryodema nigroptera	NC_046535.1	75.38	-0.18	0.16	74.38	-0.01	-0.11	76.47	0.25	-0.17	74.15	0.13	0.01
	Bryodemacris uvarovi	NC_046553.1	75.37	-0.19	0.16	74.37	-0.01	-0.11	76.47	0.25	-0.17	74.13	0.13	0.01
	Bryodemella holdereri holdereri	NC_046536.1	75.53	-0.17	0.16	74.64	-0.01	-0.11	76.10	0.24	-0.17	74.02	0.14	0.00
	Bryodemella tuberculata diluta	NC_046554.1	75.33	-0.18	0.16	74.35	-0.01	-0.12	76.37	0.26	-0.17	73.77	0.14	0.01
	Cacoplistes rogenhoferi	NC_039664.1	73.31	-0.33	0.07	73.06	-0.02	-0.11	74.33	0.41	-0.08	75.38	0.19	0.02
	Calliptamus abbreviatus	NC_030626.1	73.35	-0.16	0.14	72.72	-0.01	-0.14	75.02	0.22	-0.15	70.56	0.12	0.02
	Calliptamus barbarus	NC_046544.1	72.35	-0.18	0.16	71.59	-0.02	-0.14	73.36	0.25	-0.18	70.90	0.12	0.01
	Calliptamus italicus	NC_011305.1	73.28	-0.16	0.14	72.45	-0.01	-0.14	73.72	0.23	-0.16	70.44	0.10	0.02
	Camptonotus carolinensis	NC_028060.1	69.33	-0.31	0.07	68.06	-0.06	-0.16	72.64	0.40	-0.06	74.91	0.13	0.03
	Cardiodactylus muiri	NC_037914.1	76.48	-0.29	0.08	75.54	0.00	-0.13	77.24	0.39	-0.10	77.46	0.20	0.02
	Caryanda elegans	NC_036750.1	74.89	-0.15	0.14	74.20	0.01	-0.15	76.73	0.23	-0.15	72.65	0.11	0.03
	Caryanda sp. ZH-2016	NC_030165.1	75.27	-0.16	0.15	74.86	0.01	-0.14	76.16	0.27	-0.16	72.28	0.09	0.03
	Caryandoides hunanica	NC_053659.1	76.68	-0.13	0.11	75.84	0.00	-0.15	77.84	0.24	-0.13	74.73	0.11	0.03
	Ceracris fasciata fasciata	NC_043956.1	74.72	-0.16	0.14	73.93	-0.01	-0.13	75.62	0.23	-0.12	73.02	0.13	0.00
	Ceracris kiangsu	NC_019994.1	74.90	-0.17	0.16	74.01	0.00	-0.13	76.11	0.24	-0.14	73.49	0.12	0.04
	Ceracris versicolor	NC_025285.1	75.23	-0.15	0.13	74.44	-0.01	-0.14	75.72	0.20	-0.12	72.98	0.09	0.03
	Chondracris rosea	NC_019993.1	73.85	-0.16	0.15	72.84	0.01	-0.14	74.55	0.21	-0.17	72.44	0.10	0.02
	Choroedocus capensis	NC_041116.1	73.48	-0.16	0.14	71.64	-0.01	-0.14	74.66	0.23	-0.15	70.39	0.09	0.02
	Choroedocus violaceipes	NC_034673.1	73.23	-0.17	0.15	71.59	-0.02	-0.14	77.08	0.21	-0.17	71.02	0.07	0.02
	Chorthippus fallax	NC_048465.1	75.13	-0.15	0.14	74.84	0.01	-0.14	75.89	-0.22	0.16	71.67	0.09	0.04
	Chorthippus parallelus erythropus	NC_056786.1	75.51	-0.15	0.14	75.04	0.00	-0.14	75.89	0.25	-0.17	72.93	0.05	0.04

Order	Species	NCBI accession	Genome			PCG			rRNA			tRNA		
			AT	GC-skew	AT-skew	AT	GC-skew	AT-skew	AT	GC-skew	AT-skew	AT	GC-skew	AT-skew
	Chorthippus parallelus parallelus	NC_056785.1	75.44	-0.14	0.14	74.97	0.00	-0.14	75.88	0.25	-0.17	73.00	0.06	0.04
	Comicus campestris	NC_028062.1	75.04	-0.24	0.02	73.70	0.01	-0.14	76.42	0.38	-0.01	77.41	0.14	0.03
	Compsorhipis davidiana	NC_029408.1	75.42	-0.19	0.17	74.33	-0.02	-0.11	76.47	0.25	-0.17	74.08	0.13	0.01
	Conanalus pieli	NC_033987.1	74.31	-0.20	0.03	73.11	-0.03	-0.13	76.45	0.33	-0.03	74.78	0.14	0.01
	Conocephalus maculatus	NC_045065.1	72.26	-0.19	0.04	71.15	-0.02	-0.15	73.62	0.34	-0.04	73.84	0.13	0.03
	Conocephalus melaenus	NC_033988.1	71.54	-0.22	0.04	70.01	-0.04	-0.16	74.32	0.36	-0.04	72.91	0.14	0.03
	Conophymacris viridis	NC_046528.1	75.02	-0.17	0.13	74.43	0.00	-0.14	76.51	0.25	-0.13	72.14	0.11	0.03
	Curvipennis wixiensis	NC_031397.1	75.10	-0.14	0.13	74.66	0.01	-0.14	75.53	0.22	-0.14	73.14	0.11	0.02
	Cyphoderris monstrosa	NC_028059.1	71.70	-0.31	0.11	69.89	-0.06	-0.12	74.35	0.38	-0.15	73.47	0.14	0.03
	Dasyhippus barbipes	NC_041412.1	77.82	-0.16	0.01	76.06	0.03	-0.15	79.75	0.30	-0.02	76.96	0.08	-0.01
	Decma fissa	NC_033981.1	74.87	-0.21	0.03	74.15	0.02	-0.15	76.81	0.37	-0.05	76.00	0.17	0.03
	Deracantha onos	NC_011813.1	69.24	-0.29	0.04	67.84	-0.06	-0.15	71.38	0.41	-0.06	71.98	0.13	0.01
	Dericorys annulata	NC_046555.1	72.40	-0.17	0.13	71.75	-0.02	-0.14	73.33	0.24	-0.13	70.76	0.13	0.02
	Diabolocatantops pinguis	NC_042904.1	73.56	-0.17	0.16	72.53	0.00	-0.14	75.01	0.25	-0.16	71.40	0.10	0.01
	Dianemobius fascipes	NC_045846.1	72.28	-0.34	-0.01	71.37	-0.02	-0.15	72.89	0.43	-0.01	74.63	0.24	0.01
	Dianemobius furumagiensis	NC_045847.1	73.77	-0.33	-0.01	72.95	-0.01	-0.15	74.55	0.40	0.02	75.71	0.24	0.03
	Diestrammena asynamora	NC_033989.1	75.04	-0.19	0.10	74.20	0.01	-0.13	78.10	0.31	-0.14	75.32	0.13	0.00
	Dnopherula yuanmowensis	NC_039408.1	74.90	-0.15	0.14	74.19	0.02	-0.14	76.37	0.24	-0.16	72.78	0.12	0.02
	Ducetia japonica	NC_031652.1	74.02	-0.23	0.01	73.08	0.01	-0.16	75.50	0.37	-0.04	75.44	0.14	0.01
	Eclipophleps carinata	NC_054195.1	74.46	-0.17	0.15	73.89	0.00	-0.14	76.05	0.25	-0.14	72.16	0.10	0.01
	Ellipes minuta	NC_014488.1	66.49	-0.30	0.01	65.02	-0.08	-0.14	70.54	0.40	-0.01	70.03	0.10	0.01
	Emeiacris maculata	NC_046556.1	75.46	-0.15	0.13	75.34	0.00	-0.14	73.89	0.30	-0.12	73.97	0.11	0.02
	Epacromius coerulipes	NC_052732.1	75.51	-0.15	0.17	74.75	-0.01	-0.12	76.02	0.24	-0.19	73.79	0.11	0.02
	Ergatettix dorsifera	NC_046540.1	72.18	-0.23	0.21	70.58	-0.03	-0.11	77.64	0.30	-0.27	74.15	0.14	0.02

Order	Species	NCBI accession	Genome			PCG			rRNA			tRNA		
			AT	GC-skew	AT-skew	AT	GC-skew	AT-skew	AT	GC-skew	AT-skew	AT	GC-skew	AT-skew
	Euchorthippus fusigeniculatus	NC_014449.1	75.04	-0.15	0.14	74.46	0.01	-0.13	75.83	0.25	-0.14	73.22	0.14	0.03
	Euchorthippus unicolor	NC_045237.1	76.13	-0.14	0.14	75.59	0.01	-0.14	77.36	0.24	-0.14	73.50	0.12	0.02
	Euconocephalus nasutus	NC_053383.1	71.19	-0.24	0.03	70.18	-0.02	-0.16	74.65	0.34	-0.05	73.35	0.12	0.04
	Eumigus monticolus	BK068638	70.78	-0.20	0.17	69.52	-0.03	-0.14	72.33	0.25	-0.19	69.48	0.08	0.05
	Euparatettix bimaculatus	NC_046541.1	72.86	-0.26	0.22	71.82	-0.03	-0.11	76.54	0.34	-0.30	74.30	0.15	0.02
	Euparatettix variabilis	NC_046542.1	73.53	-0.25	0.20	72.51	-0.03	-0.11	76.20	0.31	-0.27	75.28	0.14	0.02
	Euthystira luteifemora	NC_046557.1	75.13	-0.14	0.12	74.49	0.00	-0.15	76.26	0.25	-0.12	73.44	0.12	0.03
	Eyprepocnemis plorans	BK068639	73.15	-0.17	0.12	72.39	-0.02	-0.15	72.86	0.23	-0.13	70.50	0.11	0.03
	Fer nigripennis	NC_053658.1	76.44	-0.20	0.15	75.63	-0.02	-0.13	77.16	0.26	-0.15	75.31	0.09	0.04
	Filchnerella beicki	NC_024923.1	72.26	-0.19	0.16	71.78	-0.02	-0.14	72.56	-0.05	0.03	69.47	0.11	0.02
	Filchnerella helanshanensis	NC_020329.1	72.44	-0.19	0.16	72.03	-0.02	-0.14	72.68	0.25	-0.18	69.57	0.11	0.02
	Filchnerella qilianshanensis	NC_046558.1	72.56	-0.19	0.16	72.11	-0.03	-0.14	72.60	0.26	-0.18	70.17	0.11	0.02
	Filchnerella rubrimargina	NC_052733.1	72.26	-0.19	0.16	71.70	-0.01	-0.14	72.88	0.24	-0.19	69.92	0.11	0.03
	Filchnerella tenggerensis	NC_046559.1	72.60	-0.19	0.16	72.18	-0.03	-0.14	72.54	0.25	-0.18	70.01	0.12	0.02
	Fruhstorferiola huayinensis	NC_031379.1	75.63	-0.16	0.14	74.62	0.00	-0.14	75.47	0.24	-0.15	73.51	0.12	0.01
	Fruhstorferiola kulinga	NC_026716.1	75.38	-0.15	0.14	74.82	0.00	-0.14	75.33	0.23	-0.15	73.41	0.12	0.02
	Fruhstorferiola omei	NC_046545.1	75.18	-0.15	0.14	74.70	0.00	-0.14	75.43	0.24	-0.16	73.65	0.12	0.02
	Fruhstorferiola tonkinensis	NC_031817.1	75.30	-0.16	0.13	74.59	0.00	-0.14	75.47	0.22	-0.14	73.40	0.07	0.03
	Gampsocleis gratiosa	NC_011200.1	65.31	-0.30	0.06	63.63	-0.08	-0.16	69.10	0.33	-0.06	71.53	0.16	0.03
	Gastrimargus marmoratus	NC_011114.1	75.18	-0.23	0.21	73.96	-0.05	-0.10	77.19	0.28	-0.18	74.83	0.13	0.03
	Gesonula punctifrons	NC_046411.1	75.50	-0.16	0.15	74.92	-0.01	-0.14	76.84	0.24	-0.15	73.46	0.09	0.01
	Gomphocerippus rufus	NC_014349.1	74.36	-0.16	0.14	73.71	0.00	-0.14	75.94	0.24	-0.16	72.16	0.13	0.02
	Gomphocerus licenti	NC_013847.1	74.80	-0.14	0.14	74.31	0.02	-0.15	75.86	0.25	-0.15	72.14	0.10	0.03
	Gomphocerus sibiricus	NC_021103.1	74.88	-0.13	0.13	74.44	0.02	-0.14	75.80	0.24	-0.15	72.40	0.10	0.02

Order	Species	NCBI accession	Genome			PCG			rRNA			tRNA		
			AT	GC-skew	AT-skew	AT	GC-skew	AT-skew	AT	GC-skew	AT-skew	AT	GC-skew	AT-skew
	Gomphocerus sibiricus tibetanus	NC_015478.1	74.73	-0.14	0.14	74.35	0.01	-0.14	75.72	0.22	-0.16	71.94	0.11	0.03
	Gonista bicolor	NC_029205.1	76.28	-0.13	0.14	75.69	0.01	-0.13	77.01	0.22	-0.15	74.26	0.10	0.01
	Gryllodes sigillatus	NC_057195.1	70.40	-0.31	0.07	70.06	-0.05	-0.15	71.74	0.39	-0.08	73.93	0.21	-0.01
	Gryllotalpa orientalis	NC_006678.1	70.49	-0.30	0.04	69.50	-0.04	-0.16	71.57	0.35	-0.09	73.88	0.15	0.01
	Gryllotalpa pluvialis	NC_011302.1	72.20	-0.31	0.04	71.19	-0.03	-0.16	73.80	0.35	-0.10	74.15	0.14	0.03
	Gryllotalpa unispina	NC_029148.1	70.84	-0.29	0.05	69.62	-0.04	-0.17	72.59	0.39	-0.11	74.15	0.13	0.02
	Gryllus bimaculatus	NC_053546.1	74.11	-0.30	0.09	73.58	0.00	-0.14	74.85	0.31	-0.07	76.17	0.17	-0.02
	Gryllus lineaticeps	NC_057052.1	72.53	-0.29	0.10	72.89	-0.01	-0.13	72.58	0.40	-0.12	73.98	0.14	0.00
	Gryllus veletis	NC_057053.1	73.63	-0.28	0.09	73.58	-0.01	-0.13	74.66	0.40	-0.11	76.19	0.20	0.02
	Henicus brevimucronatus	NC_028063.1	73.27	-0.24	0.06	72.21	-0.01	-0.15	75.16	0.34	-0.10	75.88	0.13	0.00
	Heteropternis respondens	NC_046537.1	75.71	-0.18	0.17	74.17	-0.02	-0.10	76.62	0.26	-0.18	73.03	0.12	0.03
	Hexacentrus japonicus	NC_033983.1	69.26	-0.28	0.05	67.69	-0.01	-0.14	73.08	0.40	-0.05	73.00	0.15	0.03
	Hexacentrus unicolor	NC_033999.1	69.97	-0.26	0.05	68.40	-0.01	-0.14	73.36	0.38	-0.05	73.26	0.13	0.04
	Hieroglyphus tonkinensis	NC_030587.1	74.18	-0.17	0.16	73.28	-0.01	-0.13	75.58	0.23	-0.18	72.21	0.09	0.03
	Holochlora fruhstorferi	NC_033993.1	70.16	-0.32	0.00	68.75	-0.05	-0.16	73.56	0.38	-0.02	74.35	0.10	0.02
	Homoeoxipha nigripes	NC_045841.1	77.63	-0.27	0.03	76.46	-0.01	-0.16	78.96	0.38	-0.05	78.90	0.22	0.01
	Homogryllacris anelytra	NC_033998.1	70.90	-0.35	0.07	69.65	-0.08	-0.15	73.23	0.45	-0.07	74.41	0.15	0.03
	Humphaplotropis culaishanensis (nomen nudum)	NC_023535.1	72.25	-0.19	0.16	71.78	-0.02	-0.14	72.77	0.25	-0.17	70.06	0.09	0.02
	Indopodisma kingdoni	NC_046529.1	75.82	-0.15	0.13	75.23	0.00	-0.14	75.74	0.22	-0.14	73.22	0.12	0.01
	Isophya major	NC_042666.1	67.25	-0.35	0.07	65.32	-0.07	-0.14	69.52	0.44	-0.09	71.82	0.13	0.03
	Kingdonella bicollina	NC_023920.1	75.61	-0.14	0.12	75.57	0.00	-0.14	75.92	0.23	-0.16	72.84	0.08	0.03
	Kuwayamaea chinensis	NC_033995.1	72.07	-0.27	0.03	70.78	-0.02	-0.15	74.23	0.40	-0.08	74.61	0.14	0.02
	Lentula callani	NC_020774.1	76.05	-0.15	0.15	75.04	0.01	-0.13	77.66	0.22	-0.16	75.41	0.08	0.06

Order	Species	NCBI accession	Genome			PCG			rRNA			tRNA		
			AT	GC-skew	AT-skew	AT	GC-skew	AT-skew	AT	GC-skew	AT-skew	AT	GC-skew	AT-skew
	Lipotactes tripyrga	NC_033996.1	72.17	-0.31	0.03	71.27	-0.08	-0.12	75.79	0.39	-0.01	75.90	0.12	0.02
	Lithidiopsis carinatus	NC_020775.1	70.32	-0.24	0.18	69.27	-0.05	-0.15	71.72	0.26	-0.18	69.98	0.09	0.02
	Locusta migratoria manilensis	NC_014891.1	75.33	-0.19	0.19	74.15	-0.04	-0.11	77.43	0.24	-0.17	74.12	0.14	0.03
	Locusta migratoria migratoria	NC_011119.1	75.53	-0.19	0.19	74.27	-0.04	-0.11	77.63	0.25	-0.18	73.96	0.15	0.02
	Locusta migratoria tibetensis	NC_015624.1	75.35	-0.17	0.18	74.28	-0.04	-0.11	77.52	0.24	-0.18	74.57	0.14	0.02
	Longchuanacris curvifurcula	NC_036994.1	75.39	-0.16	0.14	74.88	0.01	-0.15	76.03	0.27	-0.15	72.79	0.09	0.03
	Loxoblemmus doenitzi	NC_033985.1	73.25	-0.30	0.12	72.33	-0.02	-0.13	74.14	0.41	-0.18	75.61	0.22	0.01
	Mecopoda elongata	NC_021380.1	71.81	-0.25	0.01	71.15	-0.03	-0.16	73.93	0.36	-0.01	73.16	0.12	0.01
	Mecopoda niponensis	NC_021379.1	72.36	-0.23	-0.01	71.82	-0.03	-0.16	74.16	0.36	-0.01	73.47	0.15	0.03
	Mecostethus alliaceus	NC_046539.1	75.79	-0.18	0.19	74.28	-0.04	-0.10	77.09	0.24	-0.18	74.10	0.12	0.03
	Megaulacobothrus chinensis	NC_011095.1	75.11	-0.14	0.14	74.56	0.00	-0.14	76.11	0.23	-0.16	72.44	0.09	0.03
	Mekongiana xiangchengensis	NC_014450.1	74.56	-0.19	0.15	73.95	-0.01	-0.13	75.91	0.26	-0.18	73.05	0.11	0.01
	Mekongiella kingdoni	NC_023921.1	73.09	-0.21	0.16	72.20	-0.02	-0.13	75.07	0.26	-0.19	72.38	0.10	0.02
	Mekongiella xizangensis	NC_014451.1	73.55	-0.21	0.16	72.82	-0.03	-0.12	74.78	0.26	-0.19	72.78	0.06	0.03
	Melanoplus differentialis	NC_057646.1	74.78	-0.17	0.13	73.89	0.00	-0.14	75.30	0.24	-0.14	72.41	0.10	0.03
	Meloimorpha japonica	NC_039665.1	72.43	-0.29	0.07	71.70	-0.02	-0.13	74.10	0.39	-0.13	75.28	0.18	0.03
	Metrioptera bonneti	NC_033986.1	67.79	-0.28	0.03	67.46	-0.04	-0.17	71.54	0.36	-0.06	72.76	0.13	0.03
	Mirhipipteryx andensis	NC_028065.1	70.03	-0.22	-0.01	69.21	-0.05	-0.14	72.97	0.34	0.00	72.64	0.16	0.00
	Myrmecophilus manni	NC_011301.1	70.18	-0.29	0.07	69.04	-0.07	-0.14	72.81	0.35	-0.09	73.13	0.14	0.00
	Natula pravdini	NC_050742.1	78.77	-0.27	0.01	77.73	0.00	-0.15	79.31	0.38	0.01	79.71	0.22	0.01
	Nomadacris japonica	NC_036062.1	72.44	-0.17	0.16	71.21	-0.02	-0.14	73.87	0.22	-0.17	71.48	0.09	0.02
	Oecanthus sinensis	NC_034799.1	77.39	-0.25	0.02	76.92	0.02	-0.12	78.83	0.36	-0.04	77.00	0.17	0.03
	Oedaleus abruptus	NC_046538.1	75.79	-0.18	0.19	74.28	-0.04	-0.10	77.11	0.24	-0.19	74.10	0.12	0.03
	Oedaleus asiaticus	NC_011115.1	75.39	-0.18	0.19	73.93	-0.04	-0.10	77.11	0.26	-0.19	74.64	0.13	0.02

Order	Species	NCBI accession	Genome			PCG			rRNA			tRNA		
			AT	GC-skew	AT-skew	AT	GC-skew	AT-skew	AT	GC-skew	AT-skew	AT	GC-skew	AT-skew
	Oedaleus infernalis	NC_029327.1	76.01	-0.18	0.20	74.98	-0.03	-0.10	77.07	0.26	-0.20	74.30	0.06	0.06
	Oedaleus manjius	NC_052734.1	75.83	-0.18	0.19	74.98	-0.03	-0.10	76.96	0.27	-0.20	74.32	0.14	0.02
	Ognevia longipennis	NC_013701.1	76.01	-0.14	0.12	75.30	0.01	-0.14	76.47	0.21	-0.14	73.83	0.11	0.03
	Ommexecha virens	NC_020778.1	73.02	-0.19	0.17	72.28	-0.03	-0.14	74.57	0.23	-0.17	70.84	0.11	0.04
	Omocestus viridulus	NC_046560.1	74.38	-0.14	0.14	74.06	0.01	-0.14	75.98	0.25	-0.15	72.84	0.12	0.02
	Orinhippus tibetanus	NC_023467.1	74.24	-0.19	0.18	73.15	-0.03	-0.12	76.74	0.26	-0.19	72.91	0.13	0.02
	Ornebius bimaculatus	NC_039666.1	76.47	-0.32	0.02	75.03	-0.02	-0.12	76.91	0.45	-0.06	79.07	0.12	0.03
	Ornebius fuscicerci	NC_039739.1	74.95	-0.32	0.01	73.34	-0.04	-0.13	76.57	0.44	0.00	77.13	0.14	0.01
	Ornebius kanetataki	NC_039667.1	74.24	-0.34	0.00	73.02	-0.04	-0.14	75.40	0.46	0.00	77.24	0.16	0.02
	Oxya agavisa	NC_045883.1	75.82	-0.15	0.13	75.01	0.01	-0.14	78.40	0.23	-0.13	72.23	0.11	0.04
	Oxya chinensis	NC_010219.1	75.89	-0.13	0.12	75.22	0.02	-0.15	77.88	0.22	-0.13	73.29	0.09	0.05
	Oxya hainanensis	NC_045928.1	75.67	-0.14	0.13	74.97	0.01	-0.15	77.95	0.22	-0.13	72.85	0.12	0.04
	Oxya hyla	NC_032076.1	74.75	-0.16	0.14	73.88	0.01	-0.15	76.75	0.23	-0.14	72.73	0.10	0.04
	Oxya japonica	NC_043773.1	75.41	-0.13	0.13	74.77	0.02	-0.14	77.47	-0.19	0.12	72.44	0.14	0.03
	Oxytauchira brachyptera	NC_046570.1	75.89	-0.20	0.17	75.23	-0.02	-0.12	77.07	0.25	-0.15	74.69	0.15	0.05
	Oxytauchira flange	NC_053745.1	75.66	-0.20	0.18	74.85	-0.02	-0.13	77.48	0.25	-0.14	74.28	0.13	0.04
	Pacris xizangensis	NC_023919.1	74.96	-0.15	0.13	74.55	0.00	-0.14	75.32	0.23	-0.13	72.48	0.10	0.02
	Paratoacris reticulipennis	NC_053660.1	75.14	-0.16	0.15	74.43	-0.02	-0.14	77.05	0.24	-0.13	73.05	0.10	0.02
	Paratonkinacris vittifemoralis	NC_046530.1	75.97	-0.15	0.13	75.52	-0.01	-0.14	75.81	0.23	-0.14	73.41	0.10	0.05
	Pedopodisma emeiensis	NC_046561.1	76.54	-0.12	0.12	75.90	0.02	-0.14	76.58	0.20	-0.15	74.34	0.11	0.03
	Peripolus nepalensis	NC_029135.1	74.30	-0.17	0.14	73.69	-0.01	-0.14	74.79	0.27	-0.17	71.33	0.12	0.02
	Phaneroptera gracilis	NC_034756.1	72.22	-0.24	0.03	71.06	-0.03	-0.15	73.76	0.35	-0.08	74.42	0.15	0.01
	Phaneroptera nigroantennata	NC_034757.1	70.90	-0.26	0.00	70.65	-0.02	-0.16	73.79	0.36	-0.03	73.33	0.13	0.03
	Phlaeoba albonema	NC_011827.1	74.11	-0.17	0.15	73.54	0.00	-0.13	75.23	0.23	-0.17	71.74	0.10	0.03

Order	Species	NCBI accession	Genome			PCG			rRNA			tRNA		
			AT	GC-skew	AT-skew	AT	GC-skew	AT-skew	AT	GC-skew	AT-skew	AT	GC-skew	AT-skew
	Phlaeoba infumata	NC_031506.1	74.08	-0.17	0.15	73.47	0.00	-0.13	75.13	0.24	-0.17	71.89	0.11	0.02
	Phlaeoba tenebrosa	NC_029150.1	73.80	-0.18	0.16	73.09	-0.02	-0.13	76.03	0.23	-0.17	72.13	0.12	0.02
	Phryganogryllacris xiai	NC_033994.1	72.32	-0.36	0.06	71.16	-0.09	-0.14	74.14	0.44	-0.08	75.67	0.13	0.03
	Phyllomimus detersus	NC_028158.1	71.69	-0.32	0.05	70.39	-0.02	-0.12	73.88	0.43	-0.08	72.78	0.15	0.02
	Phyllomimus sinicus	NC_033997.1	71.85	-0.33	0.05	70.52	-0.02	-0.12	73.72	0.42	-0.08	73.07	0.08	0.03
	Physemacris variolosa	NC_014491.1	75.46	-0.25	0.16	73.31	-0.09	-0.09	75.99	0.29	-0.20	75.40	0.16	0.02
	Pielomastax zhengi	NC_016182.1	71.77	-0.11	0.10	70.24	-0.02	-0.13	75.43	0.20	-0.08	73.00	0.12	0.02
	Podisma pedestris	BK068645	76.02	-0.14	0.12	75.26	0.00	-0.14	76.21	0.21	-0.13	73.81	0.11	0.03
	Poecilimon luschani	NC_042665.1	69.89	-0.31	0.04	68.46	-0.03	-0.16	72.15	0.40	-0.05	72.59	0.14	0.02
	Polionemobius taprobanensis	NC_045848.1	70.34	-0.38	-0.01	69.49	-0.05	-0.17	72.85	0.45	0.02	74.93	0.20	0.02
	Prumna arctica	NC_013835.1	76.07	-0.14	0.13	75.23	-0.02	-0.14	77.55	0.22	-0.15	73.26	0.12	0.03
	Pseudocosmetura anjiensis	NC_033853.1	71.34	-0.26	0.04	69.68	-0.01	-0.15	74.65	0.40	-0.03	75.17	0.17	0.03
	Pseudoeoscyllina brevipennisoides	NC_046534.1	76.13	-0.14	0.14	75.60	0.01	-0.14	77.41	0.23	-0.14	73.28	0.11	0.02
	Pseudokuzicus pieli	NC_033982.1	73.35	-0.23	0.02	73.03	-0.01	-0.15	76.17	-0.39	0.03	75.15	0.17	0.02
	Pseudophyllus titan	NC_034773.1	72.08	-0.34	0.08	71.07	0.00	-0.14	74.15	0.43	-0.13	75.51	0.10	0.04
	Pseudorhynchus acuminatus	NC_033992.1	71.73	-0.26	0.06	70.20	-0.05	-0.15	73.78	0.39	-0.05	75.17	0.08	0.04
	Pseudorhynchus crassiceps	NC_033990.1	72.60	-0.25	0.06	71.33	-0.04	-0.14	74.74	0.39	-0.06	74.81	0.12	0.04
	Pseudothericles compressifrons	NC_028061.1	72.30	-0.18	0.13	71.48	-0.02	-0.13	75.40	0.26	-0.11	72.68	0.10	0.01
	Pseudotmethis rubimarginis	NC_020330.1	72.36	-0.19	0.16	71.86	-0.02	-0.14	72.82	0.24	-0.18	69.72	0.12	0.02
	Pseudoxya diminuta	NC_025765.1	75.90	-0.17	0.15	75.05	0.00	-0.14	77.25	0.24	-0.14	72.92	0.13	0.04
	Pternoscirta caliginosa	NC_035227.1	75.36	-0.18	0.18	74.10	0.01	-0.13	77.61	0.27	-0.18	73.87	0.14	0.03
	Pyrgacris descampsi	NC_020776.1	71.16	-0.20	0.14	69.92	-0.05	-0.13	73.41	0.28	-0.13	71.20	0.09	0.02
	Pyrgomorpha conica	BK068644	75.15	-0.18	0.11	74.43	0.01	-0.14	76.17	0.23	-0.14	73.94	0.09	0.02
	Qinlingacris taibaiensis	NC_027187.1	75.27	-0.15	0.12	75.50	0.00	-0.14	76.75	0.21	-0.14	73.44	0.11	0.02

Order	Species	NCBI accession	Genome			PCG			rRNA			tRNA		
			AT	GC-skew	AT-skew	AT	GC-skew	AT-skew	AT	GC-skew	AT-skew	AT	GC-skew	AT-skew
	Ronderosia bergii	BK068647	74.75	-0.18	0.14	73.73	0.00	-0.14	75.48	0.24	-0.12	72.82	0.12	0.03
	Ruidocollaris convexipennis	NC_046548.1	73.01	-0.32	0.00	71.34	-0.06	-0.16	75.86	0.37	-0.01	76.06	0.15	0.02
	Ruidocollaris obscura	NC_028160.1	73.48	-0.32	0.01	72.10	-0.06	-0.16	75.08	0.39	-0.03	76.34	0.12	0.02
	Ruspolia dubia	NC_009876.1	70.86	-0.24	0.02	69.98	-0.02	-0.16	73.53	0.33	-0.04	73.47	0.11	0.02
	Ruspolia lineosa	NC_033991.1	71.24	-0.23	0.03	69.84	-0.03	-0.16	74.82	0.35	-0.03	73.64	0.11	0.02
	Schistocerca gregaria gregaria	NC_013240.1	73.18	-0.17	0.16	72.23	-0.02	-0.14	74.03	0.22	-0.17	71.23	0.11	0.02
	Shirakiacris shirakii	NC_021610.1	72.33	-0.16	0.13	71.47	-0.01	-0.15	73.27	0.23	-0.15	70.22	0.10	0.03
	Shirakiacris yunkweiensis	NC_046531.1	72.52	-0.17	0.13	71.70	-0.01	-0.15	73.31	0.24	-0.15	70.07	0.10	0.03
	Shoveliteratura triangula	NC_048466.1	70.45	-0.27	0.05	70.22	-0.04	-0.15	74.29	0.40	-0.03	74.00	0.15	0.04
	Sinochlora longifissa	NC_021424.1	69.05	-0.30	0.00	68.95	-0.05	-0.15	72.34	0.36	-0.02	73.84	0.13	0.01
	Sinochlora szechwanensis	NC_034994.1	69.47	-0.31	-0.01	69.71	-0.05	-0.15	73.12	-0.13	-0.01	73.89	0.09	0.03
	Sinopodisma funiushana	NC_046546.1	76.63	-0.13	0.12	75.92	0.01	-0.14	76.47	0.21	-0.14	74.17	0.12	0.02
	Sinopodisma houshana	NC_033905.1	76.36	-0.13	0.13	75.68	0.02	-0.14	76.26	0.23	-0.15	74.18	0.10	0.01
	Sinopodisma lofaoshana	NC_046562.1	76.18	-0.13	0.13	75.58	0.01	-0.14	76.29	0.22	-0.14	73.51	0.10	0.02
	Sinopodisma lushiensis	NC_046549.1	76.73	-0.12	0.12	76.10	0.02	-0.14	76.88	0.21	-0.14	73.91	0.11	0.01
	Sinopodisma pieli	NC_051867.1	75.97	-0.13	0.13	75.37	0.00	-0.13	75.79	0.22	-0.14	73.50	0.13	0.02
	Sinopodisma qinlingensis	NC_056238.1	76.80	-0.12	0.12	76.22	0.02	-0.14	76.73	0.21	-0.14	74.03	0.11	0.01
	Sinopodisma rostellocerca	NC_052716.1	76.00	-0.14	0.13	75.46	0.00	-0.13	76.40	0.24	-0.16	73.49	0.11	0.02
	Sinopodisma tsinlingensis	NC_032303.1	75.72	-0.14	0.13	75.08	0.01	-0.14	75.82	0.23	-0.14	73.17	0.12	0.03
	Sinopodisma wudangshanensis	NC_046547.1	76.34	-0.13	0.12	75.95	0.01	-0.14	76.02	0.22	-0.14	74.26	0.11	0.02
	Sinopodisma wulingshanensis	NC_033906.1	76.48	-0.13	0.13	75.80	0.00	-0.14	76.36	0.21	-0.16	74.56	0.10	0.02
	Sinotmethis brachypterus	NC_026525.1	72.59	-0.19	0.16	72.05	-0.02	-0.14	72.79	0.26	-0.18	70.28	0.12	0.02
	Spathosternum prasiniferum prasiniferum	NC_046532.1	74.10	-0.16	0.14	73.20	0.00	-0.15	75.39	0.24	-0.16	72.41	0.12	0.01
	Sphingonotus menglaensis	NC_046550.1	74.84	-0.20	0.19	73.18	-0.02	-0.12	76.93	0.25	-0.18	73.64	0.14	0.03

Order	Species	NCBI accession	Genome			PCG			rRNA			tRNA		
			AT	GC-skew	AT-skew	AT	GC-skew	AT-skew	AT	GC-skew	AT-skew	AT	GC-skew	AT-skew
	Sphingonotus ningsianus	NC_046563.1	74.17	-0.21	0.20	72.47	-0.04	-0.11	76.48	0.24	-0.19	72.73	0.13	0.03
	Sphingonotus yenchihensis	NC_046564.1	74.90	-0.20	0.19	73.44	-0.02	-0.12	76.74	0.25	-0.19	72.73	0.13	0.03
	Stenocatantops mistshenkoi	NC_052717.1	72.85	-0.18	0.17	71.73	0.01	-0.14	74.20	0.24	-0.17	71.18	0.12	0.03
	Stenocatantops splendens	NC_041115.1	72.77	-0.18	0.17	71.67	0.00	-0.14	74.07	0.23	-0.16	70.64	0.12	0.02
	Stenopelmatus fuscus	NC_028058.1	70.34	-0.25	0.06	69.25	-0.02	-0.15	74.16	0.37	-0.06	73.49	0.11	0.00
	Svistella anhuiensis	NC_053543.1	73.75	-0.35	0.06	71.64	-0.04	-0.16	75.11	0.47	-0.08	76.86	0.26	-0.01
	Tachycines zorzini	NC_057442.1	73.91	-0.22	0.14	72.66	-0.01	-0.10	76.91	0.33	-0.16	75.00	0.12	0.00
	Tagasta indica	NC_045930.1	73.43	-0.20	0.15	72.50	0.02	-0.14	75.68	0.26	-0.19	73.43	0.12	0.03
	Tanaocerus koebelei	NC_020777.1	71.34	-0.24	0.16	69.86	-0.04	-0.14	73.56	0.27	-0.14	72.27	0.11	0.01
	Tarragoilus diuturnus	NC_021397.1	67.21	-0.28	0.08	66.03	-0.06	-0.14	69.90	0.35	-0.10	72.42	0.12	0.01
	Teleogryllus emma	NC_011823.1	73.12	-0.27	0.11	72.61	-0.01	-0.14	73.87	0.42	-0.15	74.86	0.22	-0.01
	Teleogryllus oceanicus	NC_028619.1	73.04	-0.27	0.11	72.49	0.00	-0.15	73.73	0.42	-0.15	75.09	0.22	-0.01
	Tetrix japonica	NC_018543.1	75.57	-0.21	0.19	74.79	0.00	-0.11	78.20	0.31	-0.26	75.07	0.14	0.02
	Tetrix ruyuanensis	NC_046412.1	75.50	-0.22	0.19	74.76	0.00	-0.11	78.11	0.31	-0.26	75.38	0.13	0.02
	Thrinchus schrenkii	NC_014610.1	71.25	-0.20	0.17	70.75	-0.03	-0.14	71.93	0.24	-0.18	69.43	0.09	0.03
	Tonkinacris sinensis	NC_032716.1	75.77	-0.15	0.13	75.19	0.00	-0.14	76.14	0.23	-0.15	73.68	0.08	0.03
	Traulia lofaoshana	NC_046551.1	74.37	-0.18	0.16	73.68	-0.01	-0.13	75.74	0.26	-0.16	72.42	0.11	0.02
	Traulia minuta	NC_036063.1	74.52	-0.15	0.14	73.88	0.00	-0.13	75.30	0.25	-0.16	72.62	0.10	0.01
	Traulia nigritibialis	NC_041114.1	74.63	-0.15	0.14	73.99	0.00	-0.14	75.25	0.25	-0.15	72.21	0.09	0.02
	Traulia orchotibialis	NC_046565.1	74.57	-0.15	0.14	73.91	0.00	-0.14	75.34	0.24	-0.16	72.25	0.10	0.02
	Traulia szetschuanensis	NC_013826.1	74.55	-0.16	0.14	73.95	0.00	-0.13	75.47	0.28	-0.16	72.22	0.09	0.02
	Trigonidium sjostedti	NC_032077.1	76.89	-0.29	0.02	75.94	-0.03	-0.14	78.23	0.41	-0.04	78.00	0.11	0.01
	Trilophidia annulata	NC_027179.1	74.96	-0.16	0.15	73.71	-0.02	-0.09	77.55	0.26	-0.16	72.34	0.07	0.03
	Tristira magellanica	NC_020773.1	74.68	-0.17	0.12	73.92	0.00	-0.14	74.82	0.25	-0.15	73.67	0.12	0.03

Order	Species	NCBI accession	Genome			PCG			rRNA			tRNA		
			AT	GC-skew	AT-skew	AT	GC-skew	AT-skew	AT	GC-skew	AT-skew	AT	GC-skew	AT-skew
	Troglophilus neglectus	NC_011306.1	73.37	-0.26	0.01	72.52	-0.04	-0.16	75.46	0.37	-0.03	75.38	0.10	0.05
	Truljalia hibinonis	NC_034797.1	75.39	-0.29	0.07	74.61	-0.04	-0.12	77.02	0.38	-0.09	77.77	0.18	0.01
	Uvaroviola multispinosa	NC_053942.1	75.16	-0.19	0.17	74.37	-0.02	-0.11	75.87	0.25	-0.16	74.06	0.13	0.01
	Vandiemenella viatica	BK068658	75.73	-0.16	0.12	74.66	0.00	-0.10	77.63	0.25	-0.11	73.61	0.11	0.03
	Velarifictorus hemelytrus	NC_030762.1	72.65	-0.35	0.09	71.76	-0.03	-0.14	74.53	0.44	-0.09	75.72	0.21	0.01
	Xenocatantops brachycerus	NC_021609.1	73.85	-0.16	0.16	72.87	0.00	-0.14	74.87	0.23	-0.15	72.08	0.12	0.02
	Xenogryllus marmoratus	NC_041236.1	72.12	-0.38	0.13	70.84	-0.03	-0.12	72.95	0.46	-0.15	75.84	0.23	0.00
	Xiangelilacris zhongdianensis	NC_046533.1	74.59	-0.18	0.13	74.24	0.00	-0.14	76.57	0.25	-0.13	71.89	0.11	0.03
	Xiphidiopsis gurneyi	NC_039981.1	67.50	-0.31	0.07	66.78	-0.05	-0.16	72.89	0.43	-0.06	73.93	0.15	0.01
	Xizicus fascipes	NC_018765.1	70.17	-0.28	0.05	69.55	-0.01	-0.16	74.01	0.41	-0.06	74.26	0.15	0.01
	Xizicus maculatus	NC_040974.1	69.21	-0.31	0.07	68.26	-0.04	-0.15	74.49	0.40	-0.05	74.14	0.15	0.01
	Xyleus modestus	NC_014490.1	72.47	-0.19	0.17	71.39	0.00	-0.14	73.39	0.23	-0.14	70.76	0.10	0.02
	Xyleus discoideus angulatus	BK068659	72.95	-0.19	0.17	71.77	-0.01	-0.14	73.80	0.24	-0.16	71.11	0.13	0.01
	Xyleus discoideus discoideus	BK068660	72.62	-0.19	0.16	71.68	-0.02	-0.14	73.35	0.23	-0.14	70.68	0.09	0.01
	Yunnanacris yunnaneus	NC_030586.1	75.08	-0.15	0.14	74.67	0.00	-0.14	75.38	0.24	-0.15	72.75	0.10	0.02
	Zichya baranovi	NC_033984.1	71.77	-0.28	0.03	70.70	-0.04	-0.16	73.10	0.38	-0.05	73.83	0.12	0.01
	Average		73.78	-0.21	0.11	72.90	-0.02	-0.14	75.25	0.28	-0.12	73.49	0.12	0.02
	Standard deviation (SD)		2.24	0.06	0.06	2.36	0.02	0.02	1.84	0.10	0.06	1.83	0.03	0.01
	Coefficient of variation (CV)		0.03	-0.30	0.50	0.03	-1.50	-0.11	0.02	0.36	-0.52	0.02	0.26	0.56
	Heteropteryx dilatata	NC_014680.1	76.10	-0.21	0.19	75.60	-0.02	-0.10	76.98	0.34	-0.25	78.02	0.16	-0.01
	Micadina phluctainoides	NC_014673.1	78.45	-0.10	0.17	76.81	0.00	-0.10	80.27	0.26	-0.19	79.59	0.16	0.03
	Phraortes illepidus	NC_014695.1	77.61	-0.17	0.18	76.62	-0.01	-0.10	78.46	0.25	-0.22	78.23	0.17	0.02
	Phraortes sp. Iriomote Island	NC_014705.1	78.82	-0.14	0.18	77.25	-0.01	-0.09	79.84	0.24	-0.22	80.47	0.17	0.00
Phasmatodea	*Entoria okinawaensis*	NC_014694.1	76.00	-0.15	0.15	75.68	-0.01	-0.11	78.75	0.29	-0.20	79.27	0.18	-0.01

Order	Species	NCBI accession	Genome AT	Genome GC-skew	Genome AT-skew	PCG AT	PCG GC-skew	PCG AT-skew	rRNA AT	rRNA GC-skew	rRNA AT-skew	tRNA AT	tRNA GC-skew	tRNA AT-skew
	Eurycantha calcarata	NC_058255.1	78.16	-0.22	0.15	76.73	0.00	-0.11	78.06	0.28	-0.20	80.14	0.16	0.04
	Extatosoma tiaratum	NC_017748.1	75.97	-0.26	0.20	75.23	-0.04	-0.08	77.12	0.35	-0.24	78.40	0.19	0.01
	Medauroidea extradentata	BK068643	77.59	-0.16	0.14	76.12	0.02	-0.11	78.12	0.23	-0.20	78.29	0.19	0.01
	Megacrania alpheus adan	NC_014688.1	76.87	-0.20	0.20	75.50	-0.01	-0.08	78.21	0.32	-0.23	79.03	0.17	0.00
	Phobaeticus serratipes	NC_014678.1	77.02	-0.22	0.19	75.94	-0.01	-0.09	79.21	0.28	-0.23	77.98	0.14	0.02
	Ramulus hainanense	NC_013185.1	73.10	-0.21	0.18	71.65	-0.02	-0.11	77.72	0.31	-0.24	76.73	0.18	0.02
	Ramulus mikado	NC_014702.1	75.99	-0.11	0.14	75.56	0.00	-0.11	78.62	0.26	-0.19	78.37	0.19	-0.01
	Timema bartmani	BK068649	76.11	-0.16	0.07	72.17	-0.01	-0.15	76.88	0.30	-0.14	76.36	0.17	0.01
	Timema genevievae	BK068650	75.28	-0.18	0.08	71.73	-0.01	-0.15	77.03	0.30	-0.15	75.90	0.18	0.00
	Timema monikensis	BK068651	73.56	-0.20	0.08	70.87	-0.04	-0.14	76.43	0.30	-0.12	76.57	0.14	0.00
	Timema podura	BK068652	74.42	-0.20	0.08	71.70	-0.01	-0.15	76.99	0.30	-0.14	76.38	0.16	0.01
	Timema poppensis	BK068664	73.54	-0.13	0.07	71.05	0.00	-0.14	76.70	0.28	-0.13	76.26	0.17	0.00
	Timema shepardi	BK068665	73.70	-0.16	0.07	71.14	-0.01	-0.14	76.37	0.28	-0.13	76.78	0.16	0.01
	Average		76.02	-0.18	0.14	74.30	-0.01	-0.11	77.88	0.29	-0.19	77.93	0.17	0.01
	Standard deviation (SD)		1.79	0.04	0.05	2.39	0.01	0.02	1.16	0.03	0.04	1.42	0.01	0.01
	Coefficient of variation (CV)		0.02	-0.24	0.36	0.03	-1.17	-0.22	0.01	0.11	-0.23	0.02	0.08	1.53
	Acroneuria carolinensis	NC_053852.1	62.45	-0.30	0.07	62.70	-0.05	-0.19	68.61	0.38	-0.10	67.79	0.10	-0.01
	Acroneuria hainana	NC_026104.1	68.26	0.32	-0.14	60.45	-0.08	-0.20	66.94	0.40	-0.04	66.17	0.09	-0.02
	Amphinemura bulla	NC_057056.1	66.31	-0.26	0.06	67.18	-0.03	-0.18	70.93	0.29	-0.09	70.62	0.13	-0.01
	Amphinemura longispina	NC_044748.1	69.31	-0.23	0.06	63.93	-0.05	-0.17	70.56	0.27	-0.09	70.69	0.14	0.00
	Amphinemura yao	NC_044749.1	70.94	-0.21	0.04	66.56	-0.06	-0.18	71.59	0.27	-0.10	71.02	0.13	-0.01
	Amphinemura sulcicollis	BK068636	64.59	-0.26	0.07	65.90	-0.05	-0.17	71.08	0.28	-0.09	70.77	0.12	0.00
	Antarctoperla michaelseni	NC_042199.1	67.81	-0.23	0.02	68.10	-0.03	-0.19	72.65	0.30	-0.08	70.56	0.14	0.00
Plecoptera	*Apteroperla tikumana*	NC_027698.1	67.80	-0.23	0.03	65.73	-0.02	-0.18	72.16	0.27	-0.01	70.66	0.13	-0.01

Order	Species	NCBI accession	Genome			PCG			rRNA			tRNA		
			AT	GC-skew	AT-skew	AT	GC-skew	AT-skew	AT	GC-skew	AT-skew	AT	GC-skew	AT-skew
	Brachyptera seticornis	BK068663	62.62	-0.30	0.06	65.72	-0.04	-0.19	71.48	0.28	-0.04	71.94	0.13	-0.01
	Capnia zijinshana	NC_034661.1	69.56	-0.25	0.02	68.28	0.00	-0.18	72.72	0.27	-0.03	70.53	0.11	0.00
	Diamphipnoa annulata	NC_042205.1	68.55	-0.21	0.05	66.22	-0.03	-0.16	72.28	0.28	-0.15	70.92	0.13	-0.02
	Dinocras cephalotes	NC_022843.1	70.80	-0.19	0.04	59.94	-0.08	-0.21	68.00	0.37	-0.06	67.54	0.13	-0.03
	Flavoperla hatakeyamae	NC_057436.1	69.79	-0.22	0.06	64.36	-0.01	-0.20	70.53	0.40	-0.08	70.43	0.14	-0.02
	Indonemoura jacobsoni	NC_044750.1	67.31	-0.28	0.09	67.36	-0.01	-0.17	72.25	0.32	-0.10	70.78	0.12	-0.02
	Indonemoura nohirae	NC_044751.1	69.94	-0.27	0.04	69.57	0.01	-0.18	72.93	0.30	-0.08	70.79	0.13	0.00
	Isoperla bilineata	NC_038190.1	64.14	-0.29	0.05	66.92	0.00	-0.20	70.85	0.31	-0.07	69.07	0.11	0.00
	Isoperla eximia	NC_038167.1	67.45	-0.21	0.04	66.09	-0.04	-0.18	71.11	0.31	-0.09	70.18	0.11	-0.01
	Kamimuria chungnanshana	NC_028076.1	64.78	-0.30	0.07	68.01	-0.02	-0.18	71.28	0.36	-0.07	71.71	0.13	-0.01
	Kathroperla doma	BK068661	68.94	-0.21	0.06	66.05	-0.02	-0.18	71.16	0.36	-0.12	68.97	0.14	-0.01
	Kathroperla siskiyou	BK068662	66.34	-0.26	0.08	69.95	0.00	-0.18	71.43	0.33	-0.12	69.99	0.13	0.01
	Leuctra hippopus	BK068663	68.54	-0.23	0.08	66.62	-0.02	-0.16	71.30	0.35	-0.07	69.57	0.13	-0.01
	Mesonemoura metafiligera	NC_044719.1	71.84	-0.18	0.03	66.95	0.00	-0.19	72.63	0.30	-0.09	71.16	0.14	-0.01
	Mesonemoura tritaenia	NC_044720.1	69.17	-0.19	0.04	66.46	-0.03	-0.17	72.02	0.30	-0.10	71.31	0.12	-0.01
	Nemoura meniscata	NC_057513.1	69.00	-0.20	0.06	68.94	0.01	-0.19	73.53	0.27	-0.09	71.25	0.13	-0.02
	Nemoura nankinensis	NC_034939.1	69.69	-0.19	0.04	68.95	0.01	-0.18	73.55	0.28	-0.10	71.25	0.13	0.00
	Neonemura barrosi	NC_042206.1	66.50	-0.28	0.07	68.76	-0.03	-0.15	73.96	0.30	-0.07	71.16	0.13	0.00
	Neuroperla schedingi	NC_042200.1	67.73	-0.24	0.05	66.40	-0.07	-0.17	70.33	0.36	-0.13	69.89	0.12	-0.02
	Oyamia nigribasis	NC_056285.1	71.46	-0.22	0.04	68.48	-0.03	-0.17	71.81	0.36	-0.07	70.22	0.14	-0.02
	Paragnetina indentata	NC_057280.1	70.68	-0.20	0.02	61.91	-0.07	-0.18	68.04	0.40	-0.05	68.28	0.11	0.02
	Paraleuctra cercia	NC_053557.1	69.19	-0.26	0.02	65.50	-0.01	-0.19	71.47	0.32	-0.09	68.75	0.13	0.00
	Paraperla wilsoni	BK068646	69.41	-0.20	0.03	66.68	-0.03	-0.17	72.21	0.32	-0.11	69.30	0.09	0.00
	Perlesta teaysia	NC_057281.1	69.77	-0.26	0.06	63.10	-0.04	-0.18	68.57	0.37	-0.10	67.07	0.11	-0.01

Order	Species	NCBI accession	Genome			PCG			rRNA			tRNA		
			AT	GC-skew	AT-skew	AT	GC-skew	AT-skew	AT	GC-skew	AT-skew	AT	GC-skew	AT-skew
	Perlomyia isobeae	NC_053558.1	68.83	-0.22	0.06	70.52	0.02	-0.18	72.72	0.33	-0.07	71.26	0.13	0.01
	Protonemura kohnoae	NC_044752.1	67.10	-0.24	0.07	67.22	0.01	-0.18	72.63	0.30	-0.08	70.15	0.12	-0.02
	Protonemura meyeri	NC_050322.1	66.78	-0.23	0.07	66.89	0.00	-0.19	71.76	0.29	-0.09	71.19	0.14	0.00
	Protonemura orbiculata	NC_044753.1	70.69	-0.24	0.06	67.66	0.01	-0.18	72.53	0.28	-0.08	70.71	0.13	-0.01
	Pseudomegarcys japonica	NC_038168.1	66.70	-0.30	0.09	64.07	-0.04	-0.18	71.66	0.33	-0.09	69.57	0.10	-0.02
	Pteronarcella badia	NC_029248.1	69.76	-0.15	0.02	67.02	-0.07	-0.17	70.54	0.32	-0.07	67.77	0.11	0.01
	Pteronarcys princeps	NC_006133.1	63.68	-0.31	0.05	70.48	-0.01	-0.18	71.95	0.33	-0.08	70.07	0.14	-0.02
	Rhopalopsole bulbifera	NC_042207.1	66.23	-0.26	0.06	69.87	0.02	-0.16	74.33	0.34	-0.05	71.71	0.13	0.01
	Scopura longa	NC_041105.1	68.02	-0.22	0.06	67.93	-0.02	-0.19	71.76	0.34	-0.06	70.28	0.11	0.02
	Siphonoperla torrentium	BK068648	66.51	-0.19	0.03	65.85	-0.03	-0.17	72.15	0.29	-0.11	69.99	0.12	-0.01
	Soliperla sp. ZTC-2018	NC_038189.1	67.49	-0.20	0.03	67.98	-0.03	-0.16	72.43	0.32	-0.13	70.55	0.13	0.00
	Sphaeronemoura acutispina	NC_044755.1	68.04	-0.27	0.09	67.60	-0.01	-0.18	72.90	0.31	-0.10	70.74	0.12	0.00
	Sphaeronemoura elephas	NC_057512.1	71.27	-0.24	0.08	64.57	-0.07	-0.17	71.01	0.31	-0.11	70.51	0.11	0.00
	Sphaeronemoura grandicauda	NC_044754.1	68.21	-0.23	0.04	64.32	-0.04	-0.19	71.31	0.31	-0.10	70.24	0.13	-0.01
	Styloperla spinicercia	NC_034809.1	68.37	-0.27	0.08	69.32	0.00	-0.18	73.49	0.36	-0.10	71.13	0.13	-0.01
	Suwallia errata	NC_037754.1	68.17	-0.24	0.08	64.15	-0.05	-0.17	71.49	0.34	-0.12	69.20	0.10	0.00
	Taeniopteryx ugola	NC_037897.1	70.75	-0.22	0.08	68.56	0.03	-0.20	72.72	0.28	-0.05	71.31	0.13	-0.02
	Togoperla limbata	NC_053853.1	71.53	-0.20	0.06	61.44	-0.07	-0.18	66.83	0.40	-0.03	68.67	0.10	-0.01
	Utaperla gaspesiana	BK068655	68.47	-0.18	0.01	68.79	0.00	-0.16	72.76	0.30	-0.11	69.93	0.07	0.00
	Utaperla lepnevae	BK068656	72.58	0.35	-0.17	69.79	0.01	-0.16	73.53	0.30	-0.11	69.63	0.09	-0.01
	Utaperla sopladora	BK068657	67.53	-0.21	0.09	69.16	-0.01	-0.16	73.04	0.30	-0.11	69.52	0.09	0.00
	Zelandoperla fenestrata	NC_034997.1	70.67	-0.23	0.08	64.65	-0.06	-0.17	70.93	0.30	-0.11	71.28	0.13	-0.01
	Average		68.30	-0.21	0.05	66.59	-0.02	-0.18	71.56	0.32	-0.09	70.11	0.12	-0.01
	Standard deviation (SD)		2.27	0.11	0.05	2.49	0.03	0.01	1.63	0.04	0.03	1.24	0.02	0.01

Order	Species	NCBI accession	Genome			PCG			rRNA			tRNA		
			AT	GC-skew	AT-skew	AT	GC-skew	AT-skew	AT	GC-skew	AT-skew	AT	GC-skew	AT-skew
	Coefficient of variation (CV)		0.03	-0.53	0.97	0.04	-1.17	-0.07	0.02	0.12	-0.32	0.02	0.14	-1.44
