# Supplementary material for: Expanding the mitochondrial genomic toolkit for Polyneoptera: New mitogenomes and evaluation of reduced marker sets for phylogeny and DNA barcoding
Source: Genet Mol Biol. 2026 Jul 24;49(3):e20250282. doi: 10.1590/1678-4685-GMB-2025-0282 (PMC13403772; doi:10.1590/1678-4685-GMB-2025-0282)
Supplement: Table S5 - [file 1415-4757-GMB-49-3-e20250282-s5.pdf]

Genetics and Molecular Biology

DOI: https://doi.org/10.1590/1678-4685-GMB-2025-0282

Supplementary Material to "Expanding the mitochondrial genomic toolkit for Polyneoptera: New mitogenomes and evaluation of reduced marker sets for phylogeny and DNA barcoding"

Table S5 - Codon usage patterns across newly assembled mitochondrial genomes, showing relative synonymous codon usage (RSCU) values for protein-coding genes in representative Polyneoptera species.

		**Orthoptera**									**Plecoptera**										**Phasmatodea**						
**Amino Acid**	**Codon**	***Abracris flavolineata***	***Eumigus monticolus***	***Eyprepocnemis plorans***	***Podisma pedestris***	***Pygomorpha conica***	***Ronderosia bergii***	***Vandiemenella viatica***	***Xyleus discoideus angulatus***	***Xyleus discoideus discoideus***	***Amphinemura sulcicollis***	***Brachyptera seticornis***	***Kathroperla doma***	***Kathroperla siskiyou***	***Leuctra hippopus***	***Paraperla wilsoni***	***Siphonoperla torrentium***	***Utaperla gaspesiana***	***Utaperla lepnevae***	***Utaperla sopladora***	***Medauroidea extradentata***	***Timema bartmani***	***Timema genevievae***	***Timema monikensis***	***Timema podura***	***Timema poppensis***	***Timema shepardi***
Ala	GCG	0.12	0.09	0.06	0.05	0	0	0.12	0.05	0.07	0.19	0.23	0.3	0.13	0.41	0.38	0.35	0.25	0.1	0.21	0	0.11	0.27	0.08	0.08	0.21	0.16
Ala	GCC	0.22	0.68	0.49	0.24	0.34	0.31	0.6	0.5	0.39	0.9	1	1.12	0.73	0.94	1.11	1.16	0.82	0.82	1.07	0.2	0.48	0.59	0.85	0.48	0.52	0.62
Ala	GCA	1.96	1.8	1.59	1.99	1.84	1.73	2.12	1.89	2	1.13	0.97	0.8	0.78	1.01	0.77	0.96	1.03	1.13	0.95	1.9	1.4	1.17	1.18	1.39	1.39	1.32
Ala	GCU	1.69	1.43	1.85	1.72	1.82	1.96	1.16	1.57	1.54	1.78	1.8	1.78	2.36	1.63	1.75	1.53	1.89	1.95	1.77	1.9	2.01	1.97	1.9	2.05	1.88	1.91
Arg	CGC	0.21	0.14	0.07	0	0.21	0	0.08	0	0.07	0.26	0.36	0.19	0.13	0.06	0.25	0.38	0.13	0.06	0	0.08	0.36	0.29	0.15	0.43	0.22	0.35
Arg	CGG	0.21	0.55	0.21	0.22	0.21	0.14	0.49	0.21	0.14	0.79	0.9	0.77	0.45	0.71	0.51	0.56	0.33	0.44	0.7	0.15	0.14	0.29	0.22	0.21	0.73	0.49
Arg	CGU	1.36	1.52	1.4	1.53	2.07	1.21	1.39	1.52	1.26	1.38	1.13	1.1	1.48	1.1	1.21	1	1.18	1.06	1.33	1.43	1.36	1.43	1.38	1.29	0.95	1.12
Arg	CGA	2.21	1.79	2.32	2.25	1.5	2.64	2.04	2.28	2.53	1.57	1.61	1.94	1.94	2.13	2.03	2.06	2.36	2.44	1.97	2.34	2.14	2	2.25	2.07	2.11	2.04
Asn	AAC	0.44	0.57	0.46	0.27	0.33	0.33	0.33	0.49	0.55	0.68	0.51	0.44	0.38	0.57	0.36	0.45	0.29	0.33	0.3	0.28	0.43	0.45	0.63	0.42	0.56	0.45
Asn	AAU	1.56	1.43	1.54	1.73	1.67	1.67	1.67	1.51	1.45	1.32	1.49	1.56	1.62	1.43	1.64	1.55	1.71	1.67	1.7	1.72	1.57	1.55	1.37	1.58	1.44	1.55
Asp	GAC	0.4	0.6	0.31	0.21	0.16	0.35	0.5	0.47	0.4	0.7	0.58	0.53	0.45	0.73	0.5	0.66	0.43	0.47	0.56	0.35	0.38	0.44	0.65	0.33	0.34	0.47

Amino Acid	Codon	Orthoptera									Plecoptera										Phasmatodea						
		Abracris flavolineata	***Eumigus monticolus***	***Eyprepocnemis plorans***	***Podisma pedestris***	***Pyrgomorpha conica***	***Ronderosia bergii***	***Vandiemenella viatica***	***Xyleus discoideus angulatus***	***Xyleus discoideus discoideus***	***Amphinemura sulcicollis***	***Brachyptera seticornis***	***Kathroperla doma***	***Kathroperla siskiyou***	***Leuctra hippopus***	***Paraperla wilsoni***	***Siphonoperla torrentium***	***Utaperla gaspesiana***	***Utaperla lepnevae***	***Utaperla sopladora***	***Medauroidea extradentata***	***Timema bartmani***	***Timema genevievae***	***Timema monikensis***	***Timema podura***	***Timema poppensis***	***Timema shepardi***
Asp	GAU	1.6	1.4	1.69	1.79	1.84	1.65	1.5	1.53	1.6	1.3	1.42	1.47	1.55	1.27	1.5	1.34	1.57	1.53	1.44	1.65	1.62	1.56	1.35	1.67	1.66	1.53
Cys	UGC	0.09	0.35	0.24	0.19	0.11	0.15	0.71	0.05	0.15	0.23	0.17	0.47	0.36	0.24	0.55	0.41	0.19	0.09	0.23	0.18	0.35	0.31	0.45	0.31	0.38	0.42
Cys	UGU	1.91	1.65	1.76	1.81	1.89	1.85	1.29	1.95	1.85	1.77	1.83	1.53	1.64	1.76	1.45	1.59	1.81	1.91	1.77	1.82	1.65	1.69	1.55	1.69	1.62	1.58
Gln	CAG	0.22	0.35	0.22	0.27	0.27	0.31	0.18	0.28	0.34	0.25	0.33	0.38	0.24	0.19	0.36	0.48	0.24	0.15	0.3	0.34	0.32	0.29	0.26	0.29	0.34	0.29
Gln	CAA	1.78	1.65	1.78	1.73	1.73	1.69	1.82	1.72	1.66	1.75	1.67	1.62	1.76	1.81	1.64	1.52	1.76	1.85	1.7	1.66	1.68	1.71	1.74	1.71	1.66	1.71
Glu	GAG	0.22	0.31	0.21	0.35	0.23	0.4	0.23	0.39	0.52	0.35	0.46	0.67	0.37	0.35	0.55	0.6	0.44	0.42	0.43	0.27	0.51	0.44	0.41	0.55	0.38	0.58
Glu	GAA	1.78	1.69	1.79	1.65	1.77	1.6	1.77	1.61	1.48	1.65	1.54	1.33	1.63	1.65	1.45	1.4	1.56	1.58	1.57	1.73	1.49	1.56	1.59	1.45	1.62	1.42
Gly	GGC	0.09	0.17	0.12	0.04	0.07	0.02	0.22	0.03	0.14	0.4	0.36	0.31	0.16	0.26	0.34	0.31	0.39	0.21	0.12	0.14	0.17	0.24	0.24	0.11	0.46	0.44
Gly	GGG	0.14	0.89	0.31	0.29	0.36	0.16	0.46	0.28	0.26	1.29	1.55	1.01	0.75	1.55	1.11	1.48	1.01	1.2	0.87	0.64	0.85	0.93	0.86	0.93	1.01	1.06
Gly	GGU	1.66	1.65	1.73	1.5	1.5	1.88	1.15	1.88	1.57	0.81	0.76	1.11	1.31	0.71	0.85	0.98	1.14	1.13	1.25	1.99	1.71	1.66	1.53	1.74	1.28	1.28
Gly	GGA	2.11	1.29	1.84	2.17	2.07	1.95	2.17	1.81	2.03	1.5	1.32	1.57	1.77	1.47	1.7	1.24	1.45	1.46	1.77	1.24	1.27	1.17	1.36	1.21	1.25	1.21
His	CAC	0.55	0.76	0.49	0.37	0.38	0.62	0.41	0.55	0.47	0.65	0.7	0.85	0.57	0.61	0.85	0.8	0.69	0.59	0.63	0.45	0.73	0.81	0.73	0.86	0.57	0.65
His	CAU	1.45	1.24	1.51	1.63	1.62	1.38	1.59	1.45	1.53	1.35	1.3	1.15	1.42	1.39	1.15	1.2	1.31	1.41	1.37	1.55	1.27	1.19	1.27	1.14	1.43	1.35
Ile	AUC	0.3	0.41	0.25	0.17	0.19	0.24	0.35	0.28	0.33	0.41	0.43	0.52	0.25	0.35	0.43	0.37	0.27	0.26	0.21	0.23	0.42	0.47	0.42	0.44	0.4	0.38
Ile	AUU	1.7	1.59	1.75	1.83	1.81	1.76	1.65	1.72	1.67	1.59	1.57	1.48	1.75	1.65	1.57	1.63	1.73	1.74	1.79	1.77	1.58	1.53	1.58	1.56	1.6	1.62
Leu	CUG	0.08	0.17	0.07	0.05	0.02	0.08	0.1	0.07	0.08	0.22	0.21	0.18	0.07	0.16	0.14	0.26	0.1	0.1	0.09	0.06	0.07	0.1	0.22	0.06	0.15	0.19
Leu	CUC	0.14	0.22	0.07	0.05	0.1	0.08	0.03	0.09	0.07	0.4	0.33	0.26	0.19	0.3	0.31	0.34	0.22	0.09	0.2	0.04	0.13	0.12	0.13	0.15	0.1	0.14
Leu	UUG	0.55	0.84	0.55	0.38	0.54	0.67	0.56	0.72	0.69	0.68	0.77	1.11	0.71	0.85	0.82	0.62	0.6	0.54	0.56	0.59	0.64	0.67	0.74	0.74	0.77	0.76

		Orthoptera									Plecoptera										Phasmatodea						
**Amino Acid**	**Codon**	***Abracris flavolineata***	***Eumigus monticolus***	***Eyprepocnemis plorans***	***Podisma pedestris***	***Pyrgomorpha conica***	***Ronderosia bergii***	***Vandiemenella viatica***	***Xyleus discoideus angulatus***	***Xyleus discoideus discoideus***	***Amphinemura sulcicollis***	***Brachyptera seticornis***	***Kathroperla doma***	***Kathroperla siskiyou***	***Leuctra hippopus***	***Paraperla wilsoni***	***Siphonoperla torrentium***	***Utaperla gaspesiana***	***Utaperla lepnevae***	***Utaperla sopladora***	***Medauroidea extradentata***	***Timema bartmani***	***Timema genevievae***	***Timema monikensis***	***Timema podura***	***Timema poppensis***	***Timema shepardi***
Leu	CUU	0.7	0.87	0.83	0.78	0.56	0.69	0.62	0.8	0.86	0.84	1.06	0.61	0.58	0.9	0.74	0.65	0.75	0.82	0.68	0.45	0.67	0.71	0.52	0.65	0.7	0.75
Leu	CUA	0.63	1.06	0.86	0.51	0.59	0.77	0.88	1.05	1.02	0.89	0.92	0.95	0.61	0.68	1.04	0.93	0.75	0.64	0.8	0.59	0.63	0.68	0.82	0.67	0.85	0.75
Leu	UUA	3.9	2.84	3.64	4.24	4.19	3.72	3.81	3.28	3.27	2.98	2.71	2.89	3.85	3.11	2.95	3.2	3.59	3.81	3.68	4.28	3.86	3.73	3.57	3.73	3.43	3.41
Lys	AAG	0.34	0.76	0.73	0.59	0.54	0.59	0.37	0.79	0.85	1.01	0.95	0.83	0.61	0.8	0.6	0.96	0.75	0.82	0.78	0.35	0.59	0.55	0.49	0.67	0.65	0.82
Lys	AAA	1.66	1.24	1.27	1.41	1.46	1.41	1.63	1.21	1.15	0.99	1.05	1.17	1.39	1.2	1.4	1.04	1.25	1.18	1.22	1.65	1.41	1.45	1.51	1.33	1.35	1.18
Met	AUG	0.35	0.37	0.29	0.17	0.32	0.26	0.29	0.33	0.37	0.52	0.71	0.62	0.34	0.4	0.52	0.54	0.37	0.33	0.44	0.13	0.38	0.36	0.43	0.33	0.39	0.33
Met	AUA	1.65	1.63	1.71	1.83	1.68	1.74	1.71	1.67	1.63	1.48	1.29	1.38	1.66	1.6	1.48	1.46	1.63	1.67	1.56	1.87	1.62	1.64	1.57	1.67	1.61	1.67
Phe	UUC	0.4	0.62	0.37	0.25	0.35	0.32	0.48	0.56	0.5	0.59	0.56	0.57	0.36	0.52	0.53	0.53	0.44	0.41	0.49	0.23	0.53	0.56	0.51	0.58	0.41	0.42
Phe	UUU	1.6	1.38	1.63	1.75	1.65	1.68	1.52	1.44	1.5	1.41	1.44	1.43	1.64	1.48	1.47	1.47	1.56	1.59	1.51	1.77	1.47	1.44	1.49	1.42	1.59	1.58
Pro	CCG	0.06	0.22	0.03	0.06	0.06	0.03	0.4	0.03	0.06	0.16	0.31	0.21	0.24	0.13	0.31	0.13	0.29	0.13	0.11	0.09	0.15	0.16	0.39	0.15	0.39	0.39
Pro	CCC	0.06	0.25	0.32	0.18	0.21	0.09	0.47	0.29	0.35	0.79	0.87	1.33	0.75	0.68	1.2	1.39	0.63	0.53	1.06	0.32	0.61	0.81	0.53	0.71	0.66	0.61
Pro	CCA	2.42	1.94	1.93	1.96	1.64	2.12	1.85	2.19	1.96	0.93	1.03	0.93	1.05	1	0.94	1.05	1.39	1.62	1.35	1.71	1.04	1.03	0.98	0.98	1.22	1.06
Pro	CCU	1.46	1.58	1.73	1.81	2.09	1.76	1.28	1.5	1.64	2.12	1.79	1.52	1.96	2.18	1.54	1.44	1.68	1.72	1.48	1.88	2.2	2	2.1	2.15	1.73	1.94
Ser	AGG	0.15	0.17	0	0.09	0.04	0.04	0.24	0.15	0.13	0	0.07	0.07	0	0.02	0.09	0.05	0.12	0	0.02	0.05	0.05	0.07	0.11	0.07	0.07	0.05
Ser	UCG	0.12	0.15	0.07	0.11	0.06	0.02	0.19	0.15	0.13	0.18	0.44	0.29	0.15	0.26	0.07	0.29	0.16	0.21	0.09	0.17	0.05	0.09	0.16	0.07	0.23	0.26
Ser	AGC	0.04	0.09	0.14	0.06	0.02	0	0.24	0.02	0.11	0.39	0.62	0.29	0.22	0.55	0.33	0.34	0.35	0.31	0.19	0.02	0.16	0.14	0.3	0.16	0.28	0.35
Ser	UCC	0.25	0.26	0.46	0.36	0.4	0.29	0.48	0.3	0.19	0.67	0.39	0.68	0.46	0.43	0.44	0.69	0.46	0.4	0.49	0.21	0.41	0.38	0.57	0.48	0.39	0.49
Ser	AGU	0.58	0.75	0.57	0.64	0.57	0.6	1.13	0.64	0.62	1.43	1.31	1.16	1.23	1.04	1.31	1.25	1.29	1.21	1.23	0.91	0.76	0.68	0.64	0.71	0.6	0.7

		Orthoptera									Plecoptera										Phasmatodea						
**Amino Acid**	**Codon**	***Abracris flavolineata***	***Eumigus monticolus***	***Eyprepocnemis plorans***	***Podisma pedestris***	***Pyrgomorpha conica***	***Ronderosia bergii***	***Vandiemenella viatica***	***Xyleus discoideus angulatus***	***Xyleus discoideus discoideus***	***Amphinemura sulcicollis***	***Brachyptera seticornis***	***Kathroperla doma***	***Kathroperla siskiyou***	***Leuctra hippopus***	***Paraperla wilsoni***	***Siphonoperla torrentium***	***Utaperla gaspesiana***	***Utaperla lepnevae***	***Utaperla sopladora***	***Medauroidea extradentata***	***Timema bartmani***	***Timema genevievae***	***Timema monikensis***	***Timema podura***	***Timema poppensis***	***Timema shepardi***
Ser	AGA	1.79	1.63	1.71	1.65	2.09	1.8	1.35	1.77	1.8	1.29	1.15	1.6	1.47	1.45	1.31	1.44	1.34	1.43	1.58	1.7	1.66	1.63	1.51	1.66	1.69	1.52
Ser	UCU	2.29	2.06	2.42	2.22	2.18	2.33	1.76	2.49	2.6	2.49	2.34	2.1	2.51	2.02	2.2	2.35	2.1	2.21	2.2	2.48	2.32	2.24	2.43	2.23	2.27	2.31
Ser	UCA	2.78	2.88	2.63	2.87	2.64	2.91	2.6	2.49	2.42	1.54	1.7	1.81	1.96	2.23	2.25	1.59	2.17	2.21	2.2	2.46	2.6	2.76	2.27	2.62	2.47	2.33
Ter	UAG	0.46	0.92	0.31	0.15	0.17	0.31	0.67	0.31	0.31	0.62	0.82	0.46	0.46	0.31	0.46	0.62	0.46	0.31	0.31	0.31	0.62	0.62	0.46	0.62	0.62	0.3
Ter	UAA	1.54	1.08	1.69	1.85	1.83	1.69	1.33	1.69	1.69	1.38	1.18	1.54	1.54	1.69	1.54	1.38	1.54	1.69	1.69	1.69	1.38	1.38	1.54	1.38	1.38	1.7
Thr	ACG	0.04	0.16	0.02	0.08	0.02	0.09	0.02	0.08	0.08	0.21	0.13	0.21	0.13	0.21	0.21	0.21	0.17	0.06	0.12	0	0.1	0.11	0.2	0.12	0.25	0.17
Thr	ACC	0.29	0.54	0.43	0.29	0.42	0.34	0.46	0.3	0.39	0.71	0.73	0.81	0.55	0.52	0.84	0.83	0.63	0.66	0.62	0.29	0.54	0.58	0.55	0.62	0.49	0.53
Thr	ACU	1.27	1.08	1.36	1.38	1.31	1.23	1.26	1.43	1.1	1.66	1.92	1.62	1.77	1.5	1.5	1.51	1.66	1.53	1.68	1.18	2.01	2.01	1.61	1.94	1.66	1.64
Thr	ACA	2.4	2.22	2.19	2.24	2.24	2.34	2.25	2.19	2.43	1.42	1.23	1.37	1.54	1.76	1.44	1.45	1.54	1.76	1.58	2.53	1.34	1.3	1.63	1.32	1.6	1.66
Trp	UGG	0.19	0.46	0.16	0.2	0.21	0.28	0.61	0.32	0.18	0.27	0.3	0.34	0.3	0.26	0.38	0.38	0.53	0.34	0.37	0.33	0.38	0.44	0.42	0.4	0.47	0.53
Trp	UGA	1.81	1.54	1.84	1.8	1.79	1.72	1.39	1.68	1.82	1.73	1.7	1.66	1.7	1.74	1.62	1.62	1.47	1.66	1.63	1.67	1.62	1.56	1.58	1.6	1.53	1.47
Tyr	UAC	0.31	0.39	0.29	0.24	0.22	0.19	0.27	0.24	0.34	0.77	0.63	0.56	0.39	0.57	0.5	0.56	0.45	0.36	0.4	0.18	0.29	0.25	0.54	0.29	0.42	0.37
Tyr	UAU	1.69	1.61	1.71	1.76	1.78	1.81	1.73	1.76	1.66	1.23	1.37	1.44	1.61	1.43	1.5	1.44	1.55	1.64	1.6	1.82	1.71	1.75	1.46	1.71	1.58	1.63
Val	GUC	0.13	0.29	0.13	0.12	0.13	0.11	0.17	0.1	0.15	0.54	0.5	0.48	0.22	0.57	0.46	0.54	0.27	0.23	0.28	0.11	0.18	0.17	0.2	0.16	0.25	0.23
Val	GUG	0.15	0.18	0.13	0.09	0.21	0.17	0.22	0.19	0.08	0.42	0.54	0.46	0.28	0.59	0.32	0.4	0.39	0.26	0.38	0.15	0.29	0.29	0.26	0.27	0.41	0.29
Val	GUA	1.4	1.31	1.59	1.81	1.58	1.51	2.04	1.61	1.68	1.61	1.18	1.46	1.63	1.46	1.38	1.33	1.61	1.59	1.59	1.95	1.59	1.61	1.71	1.64	1.53	1.66
Val	GUU	2.32	2.22	2.14	1.98	2.08	2.22	1.58	2.11	2.09	1.44	1.78	1.6	1.87	1.38	1.84	1.73	1.73	1.92	1.76	1.79	1.94	1.93	1.83	1.94	1.8	1.83
