# Supplementary material for: Expanding the mitochondrial genomic toolkit for Polyneoptera: New mitogenomes and evaluation of reduced marker sets for phylogeny and DNA barcoding
Source: Genet Mol Biol. 2026 Jul 24;49(3):e20250282. doi: 10.1590/1678-4685-GMB-2025-0282 (PMC13403772; doi:10.1590/1678-4685-GMB-2025-0282)
Supplement: Table S6 - [file 1415-4757-GMB-49-3-e20250282-s6.pdf]

Genetics and Molecular Biology

DOI: https://doi.org/10.1590/1678-4685-GMB-2025-0282

Supplementary Material to "Expanding the mitochondrial genomic toolkit for Polyneoptera: New mitogenomes and evaluation of reduced marker sets for phylogeny and DNA barcoding"

Table S6 - Structural characteristics of mitochondrial genomes from Polyneoptera species analyzed in this study, including genome size, gene content, and lengths of coding and non-coding regions.

					Numbers of				Total size				
**Order**	**Species**	**Family**	**Reference (NCBI access number)**	**Genome Size**	**Nº Genes**	**Nº PCG**	**Nº tRNAs**	**Nº rRNAs**	**Non-coding portion**	**Coding portion**	**PCG**	**tRNA**	**rRNA**
	Acholotermes chirotus	Termitidae	NC_034135.1	14950	37	13	22	2	286	14664	10989	1489	2186
	Aciculitermes aciculatus	Termitidae	NC_034089.1	14950	37	13	22	2	230	14720	11025	1498	2197
	Aciculitermes maymyoensis	Termitidae	NC_034107.1	14950	37	13	22	2	241	14709	11016	1498	2195
	Acidnotermes praus	Termitidae	NC_034122.1	14913	37	13	22	2	287	14626	10965	1482	2179
	Agnathotermes crassinasus	Termitidae	NC_034025.1	14950	37	13	22	2	225	14725	11041	1496	2188
	Allodontermes schultzei	Termitidae	NC_034079.1	14932	37	13	22	2	241	14691	11022	1480	2189
	Alyscotermes kilimandjaricus	Termitidae	NC_034022.1	14935	37	13	22	2	312	14623	10973	1483	2167
	Amalotermes phaeocephalus	Termitidae	NC_034099.1	14950	37	13	22	2	258	14692	11040	1478	2174
	Amitermes capito	Termitidae	NC_034038.1	14950	37	13	22	2	263	14687	10995	1499	2193
	Amitermes dentatus	Termitidae	NC_034075.1	14950	37	13	22	2	235	14715	11025	1499	2191
	Amitermes meridionalis	Termitidae	NC_034062.1	14950	37	13	22	2	282	14668	10980	1503	2185
	Amitermes obeuntis	Termitidae	NC_034124.1	14949	37	13	22	2	485	14464	10971	1499	1994
	Anhangatermes macarthuri	Termitidae	NC_034092.1	14950	37	13	22	2	261	14689	10995	1498	2196
	Anoplotermes janus	Termitidae	NC_034120.1	14778	37	13	22	2	342	14436	11004	1482	1950
	Anoplotermes parvus	Termitidae	NC_034123.1	14914	37	13	22	2	221	14693	11031	1481	2181
	Apilitermes longiceps	Termitidae	NC_034031.1	14910	37	13	22	2	224	14686	11007	1502	2177
Blattodea	*Araujotermes parvellus*	Termitidae	NC_034057.1	14950	37	13	22	2	249	14701	11022	1493	2186

Order	Species	Family	Reference (NCBI access number)	Genome Size	Numbers of					Total size			
					Nº Genes	Nº PCG	Nº tRNAs	Nº rRNAs	Non-coding portion	Coding portion	PCG	tRNA	rRNA
	Astalotermes murcus	Termitidae	NC_034132.1	14950	37	13	22	2	295	14655	10983	1485	2187
	Ateuchotermes retifaciens	Termitidae	NC_034069.1	14884	37	13	22	2	355	14529	11010	1470	2049
	Atlantitermes oculatissimus	Termitidae	NC_034148.1	14904	37	13	22	2	177	14727	11052	1492	2183
	Atlantitermes snyderi	Termitidae	NC_034102.1	14950	37	13	22	2	241	14709	11037	1492	2180
	Blaptica dubia	Blaberidae	KT893459.1	17340	37	13	22	2	2600	14740	11145	1471	2124
	Blattella bisignata	Blattellidae	NC_018549.1	16470	37	13	22	2	1775	14695	11141	1458	2096
	Blattella germanica	Blattellidae	NC_012901.1	15025	37	13	22	2	313	14712	11145	1465	2102
	Bulbitermes laticephalus	Termitidae	NC_034059.1	14897	37	13	22	2	168	14729	11031	1492	2206
	Bulbitermes makhamensis	Termitidae	NC_034029.1	14950	37	13	22	2	234	14716	11037	1488	2191
	Bulbitermes singaporiensis	Termitidae	NC_034147.1	14950	37	13	22	2	267	14683	11010	1492	2181
	Cavitermes tuberosus	Termitidae	NC_034097.1	14950	37	13	22	2	292	14658	10980	1487	2191
	Cephalotermes rectangularis	Termitidae	NC_034136.1	14949	37	13	22	2	252	14697	11034	1486	2177
	Coatitermes kartaboensis	Termitidae	NC_034141.1	14950	37	13	22	2	205	14745	11064	1491	2190
	Compositermes vindai	Termitidae	NC_034144.1	14950	37	13	22	2	291	14659	10992	1480	2187
	Constrictotermes cyphergaster	Termitidae	NC_034044.1	14950	37	13	22	2	183	14767	11079	1493	2195
	Coptotermes amanii	Rhinotermitidae	KU925200.1	14894	37	13	22	2	50	14844	11175	1495	2174
	Coptotermes elisae	Rhinotermitidae	KU925201.1	14900	37	13	22	2	33	14867	11172	1501	2194
	Coptotermes formosanus	Rhinotermitidae	NC_015800.1	16326	37	13	22	2	1615	14711	11166	1498	2047
	Coptotermes frenchi	Rhinotermitidae	KU925204.1	14912	37	13	22	2	69	14843	11172	1498	2173
	Coptotermes gestroi	Rhinotermitidae	KU925205.1	14919	37	13	22	2	79	14840	11162	1497	2181
	Coptotermes heimi	Rhinotermitidae	KU925206.1	14908	37	13	22	2	52	14856	11172	1494	2190
	Coptotermes kalshoveni	Rhinotermitidae	KU925209.1	14888	37	13	22	2	70	14818	11157	1489	2172
	Coptotermes lacteus	Rhinotermitidae	NC_018125.1	16326	37	13	22	2	1526	14800	11169	1495	2136

Order	Species	Family	Reference (NCBI access number)	Genome Size	Numbers of				Total size				
					Nº Genes	Nº PCG	Nº tRNAs	Nº rRNAs	Non-coding portion	Coding portion	PCG	tRNA	rRNA
	Coptotermes michaelseni	Rhinotermitidae	KU925212.1	14900	37	13	22	2	58	14842	11172	1494	2176
	Coptotermes remotus	Rhinotermitidae	KU925213.1	14742	37	13	22	2	854	13888	11166	1492	1230
	Coptotermes sepangensis	Rhinotermitidae	NC_030019.1	14716	37	13	22	2	837	13879	11145	1495	1239
	Coptotermes sjoestedti	Rhinotermitidae	NC_030020.1	14897	37	13	22	2	57	14840	11175	1492	2173
	Coptotermes suzhouensis	Rhinotermitidae	NC_037018.1	15764	37	13	22	2	1053	14711	11166	1498	2047
	Coptotermes testaceus	Rhinotermitidae	NC_028722.1	15752	37	13	22	2	953	14799	11167	1499	2133
	Coptotermes travians	Rhinotermitidae	NC_030021.1	14894	37	13	22	2	50	14844	11175	1486	2183
	Cornitermes cumulans	Termitidae	NC_034086.1	14950	37	13	22	2	291	14659	10986	1493	2180
	Cornitermes pugnax	Termitidae	NC_034055.1	14950	37	13	22	2	295	14655	10980	1494	2181
	Crenetermes albotarsalis	Termitidae	NC_034113.1	14911	37	13	22	2	208	14703	11043	1488	2172
	Crepititermes verruculosus	Termitidae	NC_034041.1	14950	37	13	22	2	271	14679	10998	1488	2193
	Cryptocercus changbaiensis	Cryptocercidae	NC_059066.1	15386	37	13	22	2	680	14706	11189	1449	2068
	Cryptocercus kyebangensis	Cryptocercidae	NC_030191.1	15720	37	13	22	2	1013	14707	11184	1456	2067
	Cryptocercus laojunensis	Cryptocercidae	NC_059069.1	15508	37	13	22	2	806	14702	11195	1447	2060
	Cryptocercus meridianus	Cryptocercidae	NC_037496.1	15322	37	13	22	2	622	14700	11190	1450	2060
	Cryptocercus pudacuoensis	Cryptocercidae	NC_059068.1	15580	37	13	22	2	883	14697	11171	1453	2073
	Cryptocercus relictus	Cryptocercidae	NC_018132.1	15373	37	13	22	2	677	14696	11183	1450	2063
	Cryptocercus sanchaensis	Cryptocercidae	NC_059064.1	15445	37	13	22	2	741	14704	11188	1449	2067
	Cryptocercus tianbaensis	Cryptocercidae	NC_059065.1	15473	37	13	22	2	776	14697	11176	1451	2070
	Cryptocercus weixiensis	Cryptocercidae	NC_059067.1	15439	37	13	22	2	757	14682	11174	1450	2058
	Cryptotermes declivis	Kalotermitidae	NC_045866.1	15678	37	13	22	2	956	14722	11120	1472	2130
	Cryptotermes havilandi	Kalotermitidae	NC_056118.1	15559	37	13	22	2	844	14715	11150	1453	2112
	Nitiditermes fulvus	Termitidae	NC_034033.1	14950	37	13	22	2	277	14673	10992	1500	2181
	Cubitermes oblectatus	Termitidae	NC_034056.1	14950	37	13	22	2	282	14668	10998	1499	2171

Order	Species	Family	Reference (NCBI access number)	Genome Size	Numbers of				Total size				
					Nº Genes	Nº PCG	Nº tRNAs	Nº rRNAs	Non-coding portion	Coding portion	PCG	tRNA	rRNA
	Polyspathotermes sulcifrons	Termitidae	NC_034109.1	14950	37	13	22	2	566	14384	11013	1496	1875
	Isognathotermes ugandensis	Termitidae	NC_026113.1	16491	39	13	24	2	1585	14906	11166	1562	2178
	Cylindrotermes parvignathus	Termitidae	NC_034096.1	14950	37	13	22	2	265	14685	11022	1488	2175
	Drepanotermes sp. SLC-2012	Termitidae	NC_018129.1	16542	37	13	22	2	1738	14804	11170	1501	2133
	Embiratermes brevinasus	Termitidae	NC_034101.1	14950	37	13	22	2	293	14657	10986	1496	2175
	Embiratermes neotenicus	Termitidae	NC_034930.1	15868	37	13	22	2	1231	14637	11003	1491	2143
	Ephelotermes melachoma	Termitidae	NC_034019.1	14950	37	13	22	2	248	14702	11019	1493	2190
	Ephelotermes taylori	Termitidae	NC_034149.1	14950	37	13	22	2	286	14664	10983	1493	2188
	Euhamitermes hamatus	Termitidae	NC_034064.1	14918	37	13	22	2	288	14630	10974	1486	2170
	Eupolyphaga sinensis	Corydiidae	NC_014274.1	15553	37	13	22	2	864	14689	11125	1470	2094
	Foraminitermes rhinoceros	Termitidae	NC_034116.1	14946	37	13	22	2	215	14731	11031	1490	2210
	Furculitermes cubitalis	Termitidae	NC_034131.1	14949	37	13	22	2	260	14689	11018	1487	2184
	Furculitermes longilabius	Termitidae	NC_034128.1	14950	37	13	22	2	363	14587	11060	1486	2041
	Furculitermes soyeri	Termitidae	NC_034082.1	14950	37	13	22	2	499	14451	11016	1483	1952
	Furculitermes winifredae	Termitidae	NC_034063.1	14950	37	13	22	2	207	14743	11051	1488	2204
	Geoscapheus dilatatus	Blaberidae	MW600997.1	14899	37	13	22	2	225	14674	11170	1465	2039
	Globitermes globosus	Termitidae	NC_034095.1	14950	37	13	22	2	274	14676	11025	1485	2166
	Globitermes sulphureus	Termitidae	NC_034139.1	14950	37	13	22	2	267	14683	11001	1492	2190
	Gromphadorhina portentosa	Blaberidae	NC_030001.1	15992	37	13	22	2	1376	14616	11127	1442	2047
	Havilanditermes proatripennis	Termitidae	NC_034070.1	14950	37	13	22	2	262	14688	11010	1487	2191
	Heterotermes cf. occiduus 3.12.2.AUS	Rhinotermitidae	NC_030028.1	14936	37	13	22	2	86	14850	11178	1498	2174
	Heterotermes cf. occiduus 3.15.2AUS	Rhinotermitidae	NC_030029.1	14919	37	13	22	2	68	14851	11178	1494	2179
	Heterotermes cf. paradoxus AUS103	Rhinotermitidae	NC_030023.1	14929	37	13	22	2	52	14877	11178	1497	2202

Order	Species	Family	Reference (NCBI access number)	Genome Size	Numbers of					Total size			
					Nº Genes	Nº PCG	Nº tRNAs	Nº rRNAs	Non-coding portion	Coding portion	PCG	tRNA	rRNA
	Heterotermes cf. paradoxus AUS121	Rhinotermitidae	NC_030024.1	14904	37	13	22	2	31	14873	11175	1495	2203
	Heterotermes cf. paradoxus AUS88	Rhinotermitidae	NC_030022.1	14929	37	13	22	2	52	14877	11178	1497	2202
	Heterotermes crinitus	Rhinotermitidae	KU925226.1	14882	37	13	22	2	24	14858	11178	1488	2192
	Heterotermes malabaricus	Rhinotermitidae	KU925227.1	14912	37	13	22	2	65	14847	11172	1493	2182
	Heterotermes nr. tenuis COL.4PUERTO	Rhinotermitidae	NC_030027.1	14944	37	13	22	2	79	14865	11178	1499	2188
	Heterotermes platycephalus	Rhinotermitidae	NC_030030.1	14919	37	13	22	2	68	14851	11178	1493	2180
	Heterotermes sp. SLC-2012	Rhinotermitidae	NC_018127.1	16370	37	13	22	2	1562	14808	11175	1497	2136
	Heterotermes tenuior	Rhinotermitidae	NC_030031.1	14916	37	13	22	2	77	14839	11169	1493	2177
	Heterotermes tenuis	Rhinotermitidae	KU925233.1	14940	37	13	22	2	55	14885	11177	1498	2210
	Heterotermes vagus	Rhinotermitidae	KU925234.1	14904	37	13	22	2	60	14844	11172	1495	2177
	Heterotermes validus	Rhinotermitidae	KU925235.1	14922	37	13	22	2	50	14872	11178	1500	2194
	Hirtitermes hirtiventris	Termitidae	NC_034134.1	14950	37	13	22	2	263	14687	11010	1486	2191
	Hospitalitermes hospitalis	Termitidae	NC_034074.1	14950	37	13	22	2	232	14718	11034	1490	2194
	Hospitalitermes medioflavus	Termitidae	NC_036047.1	14950	37	13	22	2	232	14718	11040	1491	2187
	Humutermes krishnai	Termitidae	NC_034129.1	14902	37	13	22	2	256	14646	10989	1481	2176
	Hypotermes makhamensis	Termitidae	NC_034037.1	14950	37	13	22	2	308	14642	10971	1491	2180
	Incisitermes minor	Kalotermitidae	NC_037511.1	15970	37	13	22	2	1233	14737	11144	1452	2141
	Inquilinitermes inquilinus	Termitidae	NC_034118.1	14950	37	13	22	2	297	14653	10983	1482	2188
	Jugositermes tuberculatus	Termitidae	NC_034083.1	14950	37	13	22	2	262	14688	11022	1487	2179
	Labiotermes labralis	Termitidae	NC_034929.1	15953	37	13	22	2	1300	14653	10997	1489	2167
	Labritermes buttelreepeni	Termitidae	NC_034058.1	14937	37	13	22	2	251	14686	11013	1490	2183
	Leucopitermes leucops	Termitidae	NC_034047.1	14950	37	13	22	2	241	14709	11016	1500	2193
	Longustitermes manni	Termitidae	NC_034094.1	14950	37	13	22	2	439	14511	11013	1480	2018

Order	Species	Family	Reference (NCBI access number)	Genome Size	Numbers of				Total size				
					Nº Genes	Nº PCG	Nº tRNAs	Nº rRNAs	Non-coding portion	Coding portion	PCG	tRNA	rRNA
	Lophotermes septentrionalis	Termitidae	NC_034105.1	14950	37	13	22	2	409	14541	10992	1496	2053
	Macrognathotermes errator	Termitidae	NC_018130.1	16330	37	13	22	2	1519	14811	11161	1493	2157
	Macrotermes annandalei	Termitidae	NC_034078.1	14950	37	13	22	2	285	14665	11000	1479	2186
	Macrotermes barneyi	Termitidae	NC_018599.1	15940	37	13	22	2	1025	14915	11159	1483	2273
	Macrotermes carbonarius	Termitidae	NC_034046.1	14853	37	13	22	2	163	14690	11031	1480	2179
	Macrotermes falciger	Termitidae	NC_034050.1	14950	37	13	22	2	260	14690	10995	1491	2204
	Macrotermes gilvus	Termitidae	NC_034110.1	14950	37	13	22	2	289	14661	11004	1481	2176
	Macrotermes malaccensis	Termitidae	NC_034030.1	14949	37	13	22	2	646	14303	10983	1487	1833
	Macrotermes muelleri	Termitidae	NC_034127.1	14950	37	13	22	2	293	14657	10992	1483	2182
	Macrotermes natalensis	Termitidae	NC_025522.1	16325	37	13	22	2	1527	14798	11159	1491	2148
	Macrotermes subhyalinus	Termitidae	NC_018128.1	16351	37	13	22	2	1557	14794	11164	1491	2139
	Macrotermes vitrialatus	Termitidae	NC_034054.1	14950	37	13	22	2	292	14658	10986	1487	2185
	Macrotermes yunnanensis	Termitidae	KU900578.1	15965	37	13	22	2	1056	14909	11156	1484	2269
	Mastotermes darwiniensis	Mastotermitidae	NC_018120.1	15487	37	13	22	2	732	14755	11169	1469	2117
	Microcerotermes baluchistanicus	Termitidae	NC_034065.1	14950	37	13	22	2	361	14589	10923	1490	2176
	Microcerotermes crassus	Termitidae	NC_034036.1	14950	37	13	22	2	298	14652	10983	1492	2177
	Microcerotermes fuscotibialis	Termitidae	NC_034067.1	14950	37	13	22	2	278	14672	11007	1492	2173
	Microcerotermes havilandi	Termitidae	NC_034084.1	14950	37	13	22	2	343	14607	10932	1494	2181
	Microcerotermes nervosus	Termitidae	NC_034104.1	14945	37	13	22	2	281	14664	10998	1495	2171
	Microcerotermes newmani	Termitidae	NC_034021.1	14950	37	13	22	2	335	14615	10935	1492	2188
	Microcerotermes parvus	Termitidae	NC_026114.1	16916	39	13	24	2	2016	14900	11169	1559	2172
	Microcerotermes progrediens	Termitidae	NC_034133.1	14950	37	13	22	2	497	14453	10980	1488	1985
	Microcerotermes serrula	Termitidae	NC_034142.1	14950	37	13	22	2	318	14632	10959	1496	2177

Order	Species	Family	Reference (NCBI access number)	Genome Size	Numbers of				Total size				
					Nº Genes	Nº PCG	Nº tRNAs	Nº rRNAs	Non-coding portion	Coding portion	PCG	tRNA	rRNA
	Microhodotermes viator	Hodotermitidae	NC_018122.1	15704	37	13	22	2	892	14812	11173	1498	2141
	Microtermes obesi	Termitidae	NC_034072.1	14907	37	13	22	2	129	14778	11103	1486	2189
	Mirocapritermes connectens	Termitidae	NC_034085.1	14950	37	13	22	2	261	14689	11013	1490	2186
	Nasutitermes arborum	Termitidae	NC_034108.1	14950	37	13	22	2	244	14706	11016	1493	2197
	Nasutitermes banksi	Termitidae	NC_034026.1	14949	37	13	22	2	285	14664	10987	1493	2184
	Nasutitermes corniger	Termitidae	NC_026115.1	16122	39	13	24	2	1210	14912	11169	1562	2181
	Nasutitermes diabolus	Termitidae	NC_034020.1	14949	37	13	22	2	252	14697	11016	1492	2189
	Nasutitermes exitiosus	Termitidae	NC_034115.1	14950	37	13	22	2	264	14686	11004	1494	2188
	Nasutitermes graveolus	Termitidae	NC_034040.1	14950	37	13	22	2	532	14418	11019	1496	1903
	Nasutitermes latifrons	Termitidae	NC_034117.1	14950	37	13	22	2	477	14473	11013	1492	1968
	Nasutitermes longipennis	Termitidae	NC_034060.1	14950	37	13	22	2	263	14687	11007	1493	2187
	Nasutitermes longirostris	Termitidae	NC_034023.1	14950	37	13	22	2	248	14702	11016	1492	2194
	Nasutitermes lujae	Termitidae	NC_034042.1	14950	37	13	22	2	278	14672	10983	1497	2192
	Nasutitermes macrocephalus	Termitidae	NC_034146.1	14950	37	13	22	2	264	14686	11004	1497	2185
	Nasutitermes matangensis	Termitidae	NC_034034.1	14950	37	13	22	2	245	14705	11034	1486	2185
	Nasutitermes neoparvus	Termitidae	NC_034080.1	14950	37	13	22	2	281	14669	10977	1504	2188
	Nasutitermes octopilis	Termitidae	NC_034045.1	14934	37	13	22	2	235	14699	11013	1496	2190
	Nasutitermes similis	Termitidae	NC_034093.1	14950	37	13	22	2	235	14715	11031	1492	2192
	Nasutitermes triodiae	Termitidae	NC_018131.1	15849	37	13	22	2	1044	14805	11167	1491	2147
	Nauphoeta cinerea	Blaberidae	NC_035052.1	15923	37	13	22	2	1286	14637	11143	1441	2053
	Neocapritermes angusticeps	Termitidae	NC_034053.1	14950	37	13	22	2	262	14688	11007	1492	2189
	Neocapritermes taracua	Termitidae	NC_026116.1	16346	37	13	22	2	1496	14850	11169	1492	2189
	Neostylopyga rhombifolia	Blattidae	NC_034842.1	15711	37	13	22	2	953	14758	11170	1477	2111
	Neotermes insularis	Kalotermitidae	NC_018124.1	15799	37	13	22	2	1050	14749	11155	1471	2123

Order	Species	Family	Reference (NCBI access number)	Genome Size	Numbers of					Total size			
					Nº Genes	Nº PCG	Nº tRNAs	Nº rRNAs	Non-coding portion	Coding portion	PCG	tRNA	rRNA
	Neotermes koshunensis	Kalotermitidae	NC_046741.1	15589	37	13	22	2	839	14750	11151	1474	2125
	Noditermes cristifrons	Termitidae	NC_034145.1	14950	37	13	22	2	240	14710	11043	1487	2180
	Occasitermes occasus	Termitidae	NC_034088.1	14950	37	13	22	2	495	14455	11028	1493	1934
	Odontotermes hainanensis	Termitidae	NC_034028.1	14950	37	13	22	2	352	14598	10935	1484	2179
	Odontotermes javanicus	Termitidae	NC_034106.1	14950	37	13	22	2	275	14675	11001	1485	2189
	Odontotermes longignathus	Termitidae	NC_034130.1	14950	37	13	22	2	304	14646	10977	1484	2185
	Odontotermes mathuri	Termitidae	NC_034035.1	14950	37	13	22	2	264	14686	11016	1484	2186
	Odontotermes minutus	Termitidae	NC_034061.1	14950	37	13	22	2	265	14685	11016	1484	2185
	Odontotermes obesus	Termitidae	NC_034027.1	14950	37	13	22	2	305	14645	10980	1486	2179
	Ophiotermes grandilabius	Termitidae	NC_034076.1	14950	37	13	22	2	232	14718	11060	1489	2169
	Ophiotermes mirandus	Termitidae	NC_034068.1	14950	37	13	22	2	192	14758	11060	1488	2210
	Opisthoplatia orientalis	Blaberidae	KT893460.1	18724	37	13	22	2	4019	14705	11142	1467	2096
	Oriensubulitermes inanis	Termitidae	NC_034087.1	14950	37	13	22	2	383	14567	11020	1494	2053
	Orientotermes emersoni	Termitidae	NC_034048.1	14950	37	13	22	2	292	14658	11004	1479	2175
	Orthotermes depressifrons	Termitidae	NC_034125.1	14949	37	13	22	2	241	14708	11043	1491	2174
	Orthotermes mansuetus	Termitidae	NC_034100.1	14894	37	13	22	2	198	14696	11031	1491	2174
	Panchlora nivea	Blaberidae	NC_030002.1	16034	37	13	22	2	1324	14710	11166	1467	2077
	Patawatermes nigripunctatus	Termitidae	NC_034032.1	14950	37	13	22	2	303	14647	11010	1471	2166
	Patawatermes turricola	Termitidae	NC_034137.1	14950	37	13	22	2	306	14644	11001	1474	2169
	Pericapritermes dolichocephalus	Termitidae	NC_034112.1	14950	37	13	22	2	437	14513	11016	1481	2016
	Pericapritermes nitobei	Termitidae	NC_039398.1	15224	37	13	22	2	449	14775	11149	1496	2130
	Periplaneta americana	Blattidae	NC_016956.1	15584	37	13	22	2	825	14759	11170	1492	2097
	Periplaneta australasiae	Blattidae	NC_034841.1	15605	37	13	22	2	831	14774	11170	1484	2120

Order	Species	Family	Reference (NCBI access number)	Genome Size	Numbers of				Total size				
					Nº Genes	Nº PCG	Nº tRNAs	Nº rRNAs	Non-coding portion	Coding portion	PCG	tRNA	rRNA
	Periplaneta brunnea	Blattidae	NC_039940.1	15604	37	13	22	2	825	14779	11176	1488	2115
	Periplaneta fuliginosa	Blattidae	NC_006076.1	14996	37	13	22	2	253	14743	11167	1475	2101
	Planicapritermes planiceps	Termitidae	NC_034090.1	14902	37	13	22	2	244	14658	10977	1498	2183
	Polyphaga plancyi	Corydiidae	NC_049567.1	15636	37	13	22	2	916	14720	11134	1482	2104
	Porotermes adamsoni	Termopsidae	NC_018121.1	16039	37	13	22	2	1305	14734	11161	1469	2104
	Postsubulitermes parviconstrictus	Termitidae	NC_034114.1	14884	37	13	22	2	213	14671	11010	1491	2170
	Proboscitermes tubuliferus	Termitidae	NC_034071.1	14949	37	13	22	2	244	14705	11022	1490	2193
	Procapritermes martyni	Termitidae	NC_034119.1	14950	37	13	22	2	355	14595	10923	1488	2184
	Procubitermes undulans	Termitidae	NC_034138.1	14950	37	13	22	2	219	14731	11055	1493	2183
	Prohamitermes mirabilis	Termitidae	NC_034039.1	14950	37	13	22	2	267	14683	11022	1500	2161
	Promirotermes pygmaeus	Termitidae	NC_034081.1	14950	37	13	22	2	242	14708	11022	1487	2199
	Protermes prorepens	Termitidae	NC_034126.1	14949	37	13	22	2	300	14649	10992	1474	2183
	Pseudacanthotermes militaris	Termitidae	NC_034077.1	14950	37	13	22	2	277	14673	10983	1485	2205
	Pseudacanthotermes spiniger	Termitidae	NC_034024.1	14950	37	13	22	2	469	14481	10977	1486	2018
	Reticulitermes aculabialis	Rhinotermitidae	NC_026695.1	16475	37	13	22	2	1762	14713	11168	1497	2048
	Reticulitermes chinensis	Rhinotermitidae	NC_025567.1	15925	37	13	22	2	1214	14711	11168	1500	2043
	Reticulitermes flaviceps	Rhinotermitidae	NC_031162.1	16485	37	13	22	2	1755	14730	11168	1498	2064
	Reticulitermes flavipes	Rhinotermitidae	NC_009498.1	16565	37	13	22	2	1849	14716	11168	1493	2055
	Reticulitermes grassei	Rhinotermitidae	KU925237.1	14910	37	13	22	2	37	14873	11171	1496	2206
	Reticulitermes hageni	Rhinotermitidae	NC_009501.1	16590	37	13	22	2	1864	14726	11168	1495	2063
	Reticulitermes labralis	Rhinotermitidae	NC_030262.1	15914	37	13	22	2	1204	14710	11168	1498	2044
	Reticulitermes leptomandibularis	Rhinotermitidae	NC_042419.1	15920	37	13	22	2	1207	14713	11168	1500	2045
	Reticulitermes lucifugus	Rhinotermitidae	NC_045240.1	16263	37	13	22	2	1565	14698	11168	1498	2032

Order	Species	Family	Reference (NCBI access number)	Genome Size	Numbers of				Total size				
					Nº Genes	Nº PCG	Nº tRNAs	Nº rRNAs	Non-coding portion	Coding portion	PCG	tRNA	rRNA
	Reticulitermes nelsonae	Rhinotermitidae	KU925238.1	14630	37	13	22	2	722	13908	11169	1494	1245
	Reticulitermes ovatilabrum	Rhinotermitidae	NC_053728.1	15913	37	13	22	2	1198	14715	11174	1496	2045
	Reticulitermes santonensis	Rhinotermitidae	NC_009499.1	16567	37	13	22	2	1850	14717	11168	1493	2056
	Reticulitermes tibialis	Rhinotermitidae	NC_045231.1	14990	37	13	22	2	194	14796	11168	1502	2126
	Reticulitermes virginicus	Rhinotermitidae	NC_009500.1	16513	37	13	22	2	1795	14718	11168	1496	2054
	Rhinotermes hispidus	Rhinotermitidae	KU925240.1	15057	37	13	22	2	167	14890	11178	1510	2202
	Roisinitermes ebogoensis	Kalotermitidae	NC_040119.1	15933	37	13	22	2	1307	14626	11138	1471	2017
	Rubeotermes jheringi	Termitidae	NC_034111.1	14898	37	13	22	2	266	14632	10974	1481	2177
	Ruptitermes arboreus	Termitidae	NC_034140.1	14846	37	13	22	2	276	14570	10995	1484	2091
	Schedorhinotermes breinli	Rhinotermitidae	NC_018126.1	15864	37	13	22	2	1047	14817	11177	1501	2139
	Periplaneta lateralis	Blattidae	NC_030003.1	15601	37	13	22	2	849	14752	11167	1488	2097
	Silvestritermes heyeri	Termitidae	NC_034066.1	14950	37	13	22	2	274	14676	10998	1489	2189
	Sphaerotermes sphaerothorax	Termitidae	NC_034103.1	14950	37	13	22	2	239	14711	11043	1484	2184
	Spinitermes trispinosus	Termitidae	NC_034091.1	14950	37	13	22	2	280	14670	11004	1492	2174
	Termes comis	Termitidae	NC_034121.1	14950	37	13	22	2	288	14662	10989	1492	2181
	Termes fatalis	Termitidae	NC_034049.1	14950	37	13	22	2	270	14680	11004	1486	2190
	Termes hospes	Termitidae	NC_026117.1	16461	37	13	22	2	1606	14855	11186	1486	2183
	Termes rostratus	Termitidae	NC_034043.1	14950	37	13	22	2	368	14582	10896	1495	2191
	Thoracotermes macrothorax	Termitidae	NC_034143.1	14950	37	13	22	2	288	14662	10980	1500	2182
	Trichotermes ducis	Termitidae	NC_034073.1	14950	37	13	22	2	265	14685	11025	1482	2178
	Tuberculitermes bycanistes	Termitidae	NC_034052.1	14950	37	13	22	2	455	14495	10998	1498	1999
	Tumulitermes pastinator	Termitidae	NC_034098.1	14950	37	13	22	2	274	14676	11004	1493	2179
	Tumulitermes recalvus	Termitidae	NC_034051.1	14950	37	13	22	2	263	14687	11013	1488	2186
	Zootermopsis angusticollis	Termopsidae	NC_018123.1	15483	37	13	22	2	817	14666	11148	1446	2072

Order	Species	Family	Reference (NCBI access number)	Genome Size	Numbers of Nº Genes	Nº PCG	Nº tRNAs	Nº rRNAs	Total size Non-coding portion	Coding portion	PCG	tRNA	rRNA
	Zootermopsis nevadensis	Termopsidae	NC_024658.1	15444	37	13	22	2	772	14672	11153	1442	2077
	Average			15237.90	37.03	13.00	22.03	2.00	546.20	14691.70	11071.94	1487.76	2132.00
	Standard deviation (SD)			565.24	0.23	0.00	0.23	0.00	544.58	134.96	82.82	15.56	124.23
	Coefficient of variation (CV)			0.04	0.01	0.00	0.01	0.00	1.00	0.01	0.01	0.01	0.06
	Amantis nawai	Gonypetidae	NC_037203.1	15684	37	13	22	2	1017	14667	11138	1459	2070
	Amorphoscelis hainana	Hymenopodidae	NC_057070.1	15792	37	13	22	2	1045	14747	11149	1487	2111
	Anaxarcha zhengi	Hymenopodidae	NC_030268.1	16620	37	13	22	2	1888	14732	11161	1466	2105
	Arria pallida	Haaniidae	NC_051892.1	16240	37	13	22	2	1471	14769	11164	1499	2106
	Creobroter gemmatus	Hymenopodidae	NC_030267.1	15716	39	13	24	2	859	14857	11150	1602	2105
	Creobroter jiangxiensis	Hymenopodidae	NC_037234.1	15801	40	13	25	2	900	14901	11136	1672	2093
	Eomantis yunnanensis	Nanomantidae	NC_037208.1	15466	37	13	22	2	758	14708	11144	1476	2088
	Titanodula formosana	Mantidae	NC_029326.1	16266	37	13	22	2	1488	14778	11151	1513	2114
	Hierodula membranacea	Mantidae	NC_048984.1	16122	37	13	22	2	1417	14705	11148	1474	2083
	Hierodula patellifera	Mantidae	NC_034283.1	16999	37	13	22	2	2264	14735	11151	1475	2109
	Humbertiella nada	Gonypetidae	NC_030264.1	15866	37	13	22	2	1138	14728	11149	1466	2113
	Leptomantella albella	Leptomantellidae	NC_024028.1	15534	37	13	22	2	872	14662	11042	1490	2130
	Mantis religiosa	Mantidae	NC_030265.1	15534	38	13	23	2	730	14804	11154	1540	2110
	Paratoxodera polyacantha	Toxoderidae	NC_037697.1	15999	41	13	26	2	860	15139	11165	1743	2231
	Pliacanthopus bimaculatus	Nanomantidae	NC_051490.1	15941	37	13	22	2	1235	14706	11146	1479	2081
	Psychomantis borneensis	Hymenopodidae	NC_045876.1	15493	37	13	22	2	790	14703	11150	1458	2095
	Rhombodera brachynota	Mantidae	NC_034282.1	16616	37	13	22	2	1883	14733	11151	1478	2104
	Hierodula longa	Mantidae	NC_051489.1	15886	37	13	22	2	1203	14683	11150	1476	2057
	Rhombodera valida	Mantidae	NC_034284.1	16308	37	13	22	2	1570	14738	11151	1476	2111
Mantodea	*Sceptuchus simplex*	Nanomantidae	NC_037206.1	15548	37	13	22	2	840	14708	11145	1481	2082

Order	Species	Family	Reference (NCBI access number)	Genome Size	Numbers of				Total size				
					Nº Genes	Nº PCG	Nº tRNAs	Nº rRNAs	Non-coding portion	Coding portion	PCG	tRNA	rRNA
	Schizocephala bicornis	Mantidae	NC_037207.1	16026	40	13	25	2	1139	14887	11159	1655	2073
	Sibylla pretiosa	Hymenopodidae	NC_037235.1	15829	37	13	22	2	1115	14714	11156	1469	2089
	Sphodromantis lineola	Mantidae	NC_037204.1	15475	37	13	22	2	762	14713	11147	1475	2091
	Tamolanica tamolana	Mantidae	NC_007702.1	16055	37	13	22	2	1317	14738	11151	1492	2095
	Tenodera sinensis	Mantidae	NC_030266.1	15531	37	13	22	2	776	14755	11152	1480	2123
	Tropidomantis tenera	Nanomantidae	NC_037205.1	15609	37	13	22	2	927	14682	11153	1466	2063
	Average			15921.38	37.50	13.00	22.50	2.00	1164.00	14757.38	11146.65	1509.50	2101.23
	Standard deviation (SD)			392.20	1.12	0.00	1.12	0.00	394.94	97.02	21.92	72.33	31.44
	Coefficient of variation (CV)			0.02	0.03	0.00	0.05	0.00	0.34	0.01	0.00	0.05	0.01
	Abracris flavolineata	Acrididae	BK068635	15681	37	13	22	2	889	14792	11220	1480	2092
	Eumigus monticolus	Tetrigidae	BK068638	15627	37	13	22	2	949	14678	11202	1409	2067
	Eyprepocnemis plorans	Pamphagidae	BK068639	15668	37	13	22	2	965	14703	11121	1478	2104
	Podisma pedestris	Trigonidiidae	BK068645	15627	37	13	22	2	823	14804	11241	1474	2089
	Pyrgomorpha conica	Pyrgacrididae	BK068644	15636	37	13	22	2	903	14733	11208	1481	2044
	Ronderosia bergii	Acrididae	BK068647	15606	37	13	22	2	835	14771	11208	1479	2084
	Vandiemenella viatica	Acrididae	BK068658	15611	37	13	22	2	951	14660	11257	1387	2016
	Xyleus discoideus angulatus	Romaleidae	BK068659	15752	37	13	22	2	939	14813	11217	1478	2118
	Xyleus discoideus discoideus	Romaleidae	BK068660	15707	37	13	22	2	924	14783	11205	1477	2101
	Gryllotalpa orientalis	Gryllotalpidae	NC_006678.1	15521	37	13	22	2	734	14787	11189	1484	2114
	Ruspolia dubia	Tettigoniidae	NC_009876.1	14971	37	13	22	2	214	14757	11191	1437	2129
	Anabrus simplex	Tettigoniidae	NC_009967.1	15766	37	13	22	2	1013	14753	11198	1458	2097
	Oxya chinensis	Acrididae	NC_010219.1	15443	37	13	22	2	715	14728	11230	1441	2057
	Megaulacobothrus chinensis	Acrididae	NC_011095.1	15599	37	13	22	2	820	14779	11195	1477	2107
Orthoptera	*Gastrimargus marmoratus*	Acrididae	NC_011114.1	15924	37	13	22	2	1162	14762	11180	1470	2112

Order	Species	Family	Reference (NCBI access number)	Genome Size	Numbers of				Total size				
					Nº Genes	Nº PCG	Nº tRNAs	Nº rRNAs	Non-coding portion	Coding portion	PCG	tRNA	rRNA
	Oedaleus asiaticus	Acrididae	NC_011115.1	16259	37	13	22	2	1653	14606	11126	1435	2045
	Locusta migratoria migratoria	Acrididae	NC_011119.1	16053	37	13	22	2	1242	14811	11194	1499	2118
	Gampsocleis gratiosa	Tettigoniidae	NC_011200.1	15929	37	13	22	2	1091	14838	11185	1484	2169
	Myrmecophilus manni	Gryllidae	NC_011301.1	15323	37	13	22	2	554	14769	11228	1450	2091
	Gryllotalpa pluvialis	Gryllotalpidae	NC_011302.1	15525	37	13	22	2	822	14703	11183	1450	2070
	Acrida willemsei	Acrididae	NC_011303.1	15601	37	13	22	2	915	14686	11178	1476	2032
	Calliptamus italicus	Acrididae	NC_011305.1	15675	37	13	22	2	882	14793	11185	1485	2123
	Troglophilus neglectus	Rhaphidophoridae	NC_011306.1	15810	38	13	23	2	1034	14776	11176	1475	2125
	Deracantha onos	Tettigoniidae	NC_011813.1	15650	37	13	22	2	881	14769	11215	1454	2100
	Teleogryllus emma	Gryllidae	NC_011823.1	15660	37	13	22	2	981	14679	11143	1475	2061
	Atractomorpha sinensis	Pyrgomorphidae	NC_011824.1	15558	37	13	22	2	803	14755	11163	1462	2130
	Phlaeoba albonema	Acrididae	NC_011827.1	15657	37	13	22	2	823	14834	11211	1470	2153
	Schistocerca gregaria gregaria	Acrididae	NC_013240.1	15625	37	13	22	2	755	14870	11216	1470	2184
	Ognevia longipennis	Acrididae	NC_013701.1	15621	37	13	22	2	832	14789	11172	1471	2146
	Arcyptera coreana	Acrididae	NC_013805.1	15783	37	13	22	2	1011	14772	11194	1477	2101
	Traulia szetschuanensis	Acrididae	NC_013826.1	15768	37	13	22	2	997	14771	11197	1461	2113
	Prumna arctica	Acrididae	NC_013835.1	15628	37	13	22	2	841	14787	11212	1485	2090
	Gomphocerus licenti	Acrididae	NC_013847.1	15597	37	13	22	2	783	14814	11186	1473	2155
	Gomphocerippus rufus	Acrididae	NC_014349.1	15598	37	13	22	2	772	14826	11203	1470	2153
	Euchorthippus fusigeniculatus	Acrididae	NC_014449.1	15772	37	13	22	2	1125	14647	11106	1439	2102
	Mekongiana xiangchengensis	Pyrgomorphidae	NC_014450.1	15567	37	13	22	2	743	14824	11239	1464	2121
	Mekongiella xizangensis	Pyrgomorphidae	NC_014451.1	15885	37	13	22	2	1060	14825	11182	1484	2159
	Ellipes minuta	Tridactylidae	NC_014488.1	15451	37	13	22	2	732	14719	11182	1462	2075

Order	Species	Family	Reference (NCBI access number)	Genome Size	Numbers of				Total size				
					Nº Genes	Nº PCG	Nº tRNAs	Nº rRNAs	Non-coding portion	Coding portion	PCG	tRNA	rRNA
	Xyleus modestus	Romaleidae	NC_014490.1	15723	37	13	22	2	948	14775	11221	1457	2097
	Physemacris variolosa	Pneumoridae	NC_014491.1	17004	37	13	22	2	2114	14890	11257	1466	2167
	Thrinchus schrenkii	Pamphagidae	NC_014610.1	15672	37	13	22	2	1042	14630	11107	1440	2083
	Acrida cinerea	Acrididae	NC_014887.1	15599	37	13	22	2	834	14765	11192	1475	2098
	Locusta migratoria manilensis	Acrididae	NC_014891.1	15895	37	13	22	2	1050	14845	11249	1452	2144
	Gomphocerus sibiricus tibetanus	Acrididae	NC_015478.1	15571	37	13	22	2	755	14816	11179	1475	2162
	Locusta migratoria tibetensis	Acrididae	NC_015624.1	15568	37	13	22	2	731	14837	11216	1472	2149
	Pielomastax zhengi	Episactidae	NC_016182.1	15602	37	13	22	2	784	14818	11235	1467	2116
	Alulatettix yunnanensis	Tetrigidae	NC_018542.1	15104	37	13	22	2	417	14687	11121	1439	2127
	Tetrix japonica	Tetrigidae	NC_018543.1	15128	37	13	22	2	439	14689	11128	1456	2105
	Xizicus fascipes	Tettigoniidae	NC_018765.1	16166	37	13	22	2	1404	14762	11185	1473	2104
	Chondracris rosea	Acrididae	NC_019993.1	15646	37	13	22	2	766	14880	11223	1560	2097
	Ceracris kiangsu	Acrididae	NC_019994.1	15665	37	13	22	2	1205	14460	11199	1486	1775
	Asiotmethis zacharjini	Pamphagidae	NC_020328.1	15660	37	13	22	2	832	14828	11183	1481	2164
	Filchnerella helanshanensis	Pamphagidae	NC_020329.1	15657	37	13	22	2	901	14756	11192	1458	2106
	Pseudotmethis rubimarginis	Pamphagidae	NC_020330.1	15661	37	13	22	2	840	14821	11222	1473	2126
	Tristira magellanica	Tristiridae	NC_020773.1	16494	37	13	22	2	1524	14970	11201	1468	2301
	Lentula callani	Lentulidae	NC_020774.1	15944	37	13	22	2	1114	14830	11185	1473	2172
	Lithidiopsis carinatus	Acrididae	NC_020775.1	15652	37	13	22	2	869	14783	11206	1464	2113
	Pyrgacris descampsi	Pyrgacrididae	NC_020776.1	15618	37	13	22	2	800	14818	11212	1478	2128
	Tanaocerus koebelei	Pneumoridae	NC_020777.1	15515	37	13	22	2	720	14795	11232	1484	2079
	Ommexecha virens	Ommexechidae	NC_020778.1	15536	37	13	22	2	763	14773	11183	1472	2118
	Gomphocerus sibiricus	Acrididae	NC_021103.1	15590	37	13	22	2	762	14828	11202	1469	2157

Order	Species	Family	Reference (NCBI access number)	Genome Size	Numbers of				Total size				
					Nº Genes	Nº PCG	Nº tRNAs	Nº rRNAs	Non-coding portion	Coding portion	PCG	tRNA	rRNA
	Mecopoda niponensis	Tettigoniidae	NC_021379.1	15364	37	13	22	2	679	14685	11193	1439	2053
	Mecopoda elongata	Tettigoniidae	NC_021380.1	15284	37	13	22	2	573	14711	11184	1470	2057
	Tarragoilus diuturnus	Prophalangopsidae	NC_021397.1	16144	37	13	22	2	1404	14740	11171	1468	2101
	Sinochlora longifissa	Tettigoniidae	NC_021424.1	18133	37	13	22	2	3315	14818	11195	1480	2143
	Xenocatantops brachycerus	Acrididae	NC_021609.1	15605	37	13	22	2	872	14733	11196	1469	2068
	Shirakiacris shirakii	Acrididae	NC_021610.1	15649	37	13	22	2	826	14823	11214	1468	2141
	Orinhippus tibetanus	Acrididae	NC_023467.1	15611	37	13	22	2	874	14737	11181	1468	2088
	Humphaplotropis culaishanensis (nomen nudum)	Pamphagidae	NC_023535.1	15659	37	13	22	2	1010	14649	11169	1436	2044
	Pacris xizangensis	Acrididae	NC_023919.1	15622	37	13	22	2	842	14780	11168	1462	2150
	Kingdonella bicollina	Acrididae	NC_023920.1	15630	37	13	22	2	864	14766	11185	1475	2106
	Mekongiella kingdoni	Pyrgomorphidae	NC_023921.1	15932	37	13	22	2	1101	14831	11240	1466	2125
	Filchnerella beicki	Pamphagidae	NC_024923.1	15658	37	13	22	2	891	14767	11185	1476	2106
	Ceracris versicolor	Acrididae	NC_025285.1	15616	37	13	22	2	870	14746	11177	1473	2096
	Angaracris barabensis	Acrididae	NC_025558.1	15930	37	13	22	2	1166	14764	11149	1466	2149
	Pseudoxya diminuta	Acrididae	NC_025765.1	15541	37	13	22	2	971	14570	11083	1442	2045
	Asiotmethis jubatus	Pamphagidae	NC_025904.1	15669	37	13	22	2	835	14834	11178	1489	2167
	Angaracris rhodopa	Acrididae	NC_025946.1	15930	37	13	22	2	1162	14768	11153	1466	2149
	Sinotmethis brachypterus	Pamphagidae	NC_026525.1	15662	37	13	22	2	841	14821	11185	1480	2156
	Fruhstorferiola kulinga	Acrididae	NC_026716.1	15655	37	13	22	2	821	14834	11183	1484	2167
	Trilophidia annulata	Acrididae	NC_027179.1	15775	37	13	22	2	998	14777	11184	1465	2128
	Qinlingacris taibaiensis	Acrididae	NC_027187.1	15774	37	13	22	2	1714	14060	10499	1462	2099
	Stenopelmatus fuscus	Stenopelmatidae	NC_028058.1	15767	37	13	22	2	972	14795	11201	1478	2116
	Cyphoderris monstrosa	Prophalangopsidae	NC_028059.1	16590	37	13	22	2	1747	14843	11191	1478	2174

Order	Species	Family	Reference (NCBI access number)	Genome Size	Numbers of				Total size				
					Nº Genes	Nº PCG	Nº tRNAs	Nº rRNAs	Non-coding portion	Coding portion	PCG	tRNA	rRNA
	Camptonotus carolinensis	Gryllacrididae	NC_028060.1	15211	37	13	22	2	537	14674	11165	1451	2058
	Pseudothericles compressifrons	Thericleidae	NC_028061.1	15081	37	13	22	2	253	14828	11219	1462	2147
	Comicus campestris	Schizodactylidae	NC_028062.1	15691	37	13	22	2	847	14844	11188	1500	2156
	Henicus brevimucronatus	Anostostomatidae	NC_028063.1	15140	37	13	22	2	341	14799	11217	1445	2137
	Mirhipipteryx andensis	Ripipterygidae	NC_028065.1	15307	37	13	22	2	629	14678	11215	1436	2027
	Phyllomimus detersus	Tettigoniidae	NC_028158.1	16007	37	13	22	2	1818	14189	11176	1482	1531
	Ruidocollaris obscura	Tettigoniidae	NC_028160.1	16424	37	13	22	2	1661	14763	11184	1476	2103
	Teleogryllus oceanicus	Gryllidae	NC_028619.1	15660	37	13	22	2	849	14811	11223	1465	2123
	Peripolus nepalensis	Acrididae	NC_029135.1	15858	37	13	22	2	1066	14792	11201	1474	2117
	Gryllotalpa unispina	Gryllotalpidae	NC_029148.1	15513	37	13	22	2	964	14549	11136	1447	1966
	Phlaeoba tenebrosa	Acrididae	NC_029150.1	15648	37	13	22	2	810	14838	11196	1486	2156
	Gonista bicolor	Acrididae	NC_029205.1	15618	37	13	22	2	801	14817	11178	1478	2161
	Oedaleus infernalis	Acrididae	NC_029327.1	15898	37	13	22	2	1080	14818	11198	1471	2149
	Compsorhipis davidiana	Acrididae	NC_029408.1	16085	37	13	22	2	1365	14720	11256	1399	2065
	Caryanda sp. ZH-2016	Acrididae	NC_030165.1	15445	37	13	22	2	640	14805	11185	1472	2148
	Yunnanacris yunnaneus	Acrididae	NC_030586.1	15475	37	13	22	2	710	14765	11218	1458	2089
	Hieroglyphus tonkinensis	Acrididae	NC_030587.1	15625	37	13	22	2	865	14760	11224	1448	2088
	Calliptamus abbreviatus	Acrididae	NC_030626.1	15558	37	13	22	2	533	15025	11183	1488	2354
	Velarifictorus hemelytrus	Gryllidae	NC_030762.1	16123	37	13	22	2	1506	14617	11142	1417	2058
	Fruhstorferiola huayinensis	Acrididae	NC_031379.1	16227	37	13	22	2	1386	14841	11183	1486	2172
	Curvipennis wixiensis	Acrididae	NC_031397.1	15528	37	13	22	2	767	14761	11179	1475	2107
	Phlaeoba infumata	Acrididae	NC_031506.1	15642	37	13	22	2	841	14801	11217	1470	2114
	Ducetia japonica	Tettigoniidae	NC_031652.1	16276	37	13	22	2	1453	14823	11188	1477	2158

Order	Species	Family	Reference (NCBI access number)	Genome Size	Numbers of Nº Genes	Nº PCG	Nº tRNAs	Nº rRNAs	Non-coding portion	Total size Coding portion	PCG	tRNA	rRNA
	Fruhstorferiola tonkinensis	Acrididae	NC_031817.1	15638	37	13	22	2	817	14821	11182	1474	2165
	Oxya hyla	Acrididae	NC_032076.1	15483	37	13	22	2	625	14858	11218	1475	2165
	Trigonidium sjostedti	Trigonidiidae	NC_032077.1	15763	37	13	22	2	995	14768	11188	1463	2117
	Sinopodisma tsinlingensis	Acrididae	NC_032303.1	15627	37	13	22	2	863	14764	11179	1475	2110
	Tonkinacris sinensis	Acrididae	NC_032716.1	15627	37	13	22	2	1016	14611	11094	1434	2083
	Pseudocosmetura anjiensis	Tettigoniidae	NC_033853.1	16044	37	13	22	2	1490	14554	11104	1424	2026
	Sinopodisma houshana	Acrididae	NC_033905.1	15818	37	13	22	2	1096	14722	11176	1459	2087
	Sinopodisma wulingshanensis	Acrididae	NC_033906.1	15872	37	13	22	2	1041	14831	11188	1476	2167
	Decma fissa	Tettigoniidae	NC_033981.1	16122	37	13	22	2	1387	14735	11184	1467	2084
	Pseudokuzicus pieli	Tettigoniidae	NC_033982.1	16077	37	13	22	2	1314	14763	11218	1454	2091
	Hexacentrus japonicus	Tettigoniidae	NC_033983.1	16120	37	13	22	2	1349	14771	11177	1472	2122
	Zichya baranovi	Tettigoniidae	NC_033984.1	15645	37	13	22	2	869	14776	11171	1474	2131
	Loxoblemmus doenitzi	Gryllidae	NC_033985.1	15396	37	13	22	2	598	14798	11182	1467	2149
	Metrioptera bonneti	Tettigoniidae	NC_033986.1	16256	37	13	22	2	1487	14769	11177	1479	2113
	Conanalus pieli	Tettigoniidae	NC_033987.1	15820	37	13	22	2	1020	14800	11180	1470	2150
	Conocephalus melaenus	Tettigoniidae	NC_033988.1	15852	37	13	22	2	1027	14825	11204	1464	2157
	Diestrammena asynamora	Rhaphidophoridae	NC_033989.1	15309	37	13	22	2	659	14650	11138	1441	2071
	Pseudorhynchus crassiceps	Tettigoniidae	NC_033990.1	15865	37	13	22	2	989	14876	11240	1466	2170
	Ruspolia lineosa	Tettigoniidae	NC_033991.1	16110	37	13	22	2	1346	14764	11196	1437	2131
	Pseudorhynchus acuminatus	Tettigoniidae	NC_033992.1	16056	37	13	22	2	1280	14776	11221	1457	2098
	Holochlora fruhstorferi	Tettigoniidae	NC_033993.1	15899	37	13	22	2	1140	14759	11221	1451	2087
	Phryganogryllacris xiai	Gryllacrididae	NC_033994.1	15876	37	13	22	2	1067	14809	11183	1487	2139
	Kuwayamaea chinensis	Tettigoniidae	NC_033995.1	15875	37	13	22	2	1119	14756	11204	1469	2083
	Lipotactes tripyrga	Tettigoniidae	NC_033996.1	15949	37	13	22	2	1192	14757	11208	1461	2088

Order	Species	Family	Reference (NCBI access number)	Genome Size	Numbers of				Total size				
					Nº Genes	Nº PCG	Nº tRNAs	Nº rRNAs	Non-coding portion	Coding portion	PCG	tRNA	rRNA
	Phyllomimus sinicus	Tettigoniidae	NC_033997.1	15692	37	13	22	2	945	14747	11227	1455	2065
	Homogryllacris anelytra	Gryllacrididae	NC_033998.1	15706	37	13	22	2	971	14735	11181	1470	2084
	Hexacentrus unicolor	Tettigoniidae	NC_033999.1	15752	37	13	22	2	946	14806	11183	1472	2151
	Choroedocus violaceipes	Acrididae	NC_034673.1	15694	37	13	22	2	924	14770	11183	1476	2111
	Aiolopus thalassinus	Acrididae	NC_034674.1	15753	37	13	22	2	1018	14735	11176	1466	2093
	Phaneroptera gracilis	Tettigoniidae	NC_034756.1	18255	37	13	22	2	3427	14828	11186	1477	2165
	Phaneroptera nigroantennata	Tettigoniidae	NC_034757.1	16832	37	13	22	2	1980	14852	11180	1486	2186
	Pseudophyllus titan	Tettigoniidae	NC_034773.1	16227	37	13	22	2	1419	14808	11188	1482	2138
	Truljalia hibinonis	Gryllidae	NC_034797.1	15120	37	13	22	2	332	14788	11201	1466	2121
	Oecanthus sinensis	Gryllidae	NC_034799.1	16142	37	13	22	2	1481	14661	11163	1449	2049
	Sinochlora szechwanensis	Tettigoniidae	NC_034994.1	18051	37	13	22	2	3294	14757	11218	1450	2089
	Pternoscirta caliginosa	Acrididae	NC_035227.1	15598	37	13	22	2	778	14820	11212	1475	2133
	Anabropsis carli	Anostostomatidae	NC_035420.1	15932	37	13	22	2	1096	14836	11183	1486	2167
	Anabropsis carnarius	Anostostomatidae	NC_035552.1	16119	37	13	22	2	1332	14787	11161	1477	2149
	Anabropsis crenatis	Anostostomatidae	NC_035553.1	16099	37	13	22	2	1278	14821	11212	1480	2129
	Nomadacris japonica	Acrididae	NC_036062.1	15638	37	13	22	2	1160	14478	11059	1433	1986
	Traulia minuta	Acrididae	NC_036063.1	15636	37	13	22	2	853	14783	11180	1482	2121
	Caryanda elegans	Acrididae	NC_036750.1	15459	37	13	22	2	645	14814	11184	1481	2149
	Longchuanacris curvifurcula	Acrididae	NC_036994.1	15450	37	13	22	2	652	14798	11182	1467	2149
	Cardiodactylus muiri	Gryllidae	NC_037914.1	16328	37	13	22	2	1596	14732	11207	1442	2083
	Dnopherula yuanmowensis	Acrididae	NC_039408.1	15629	37	13	22	2	771	14858	11229	1483	2146
	Cacoplistes rogenhoferi	Phalangopsidae	NC_039664.1	16018	37	13	22	2	1328	14690	11203	1446	2041
	Meloimorpha japonica	Phalangopsidae	NC_039665.1	15880	37	13	22	2	1102	14778	11144	1473	2161
	Ornebius bimaculatus	Mogoplistidae	NC_039666.1	16136	37	13	22	2	1361	14775	11188	1480	2107

Order	Species	Family	Reference (NCBI access number)	Genome Size	Numbers of				Total size				
					Nº Genes	Nº PCG	Nº tRNAs	Nº rRNAs	Non-coding portion	Coding portion	PCG	tRNA	rRNA
	Ornebius kanetataki	Mogoplistidae	NC_039667.1	16589	37	13	22	2	1887	14702	11184	1448	2070
	Ornebius fuscicerci	Mogoplistidae	NC_039739.1	16368	37	13	22	2	1551	14817	11173	1473	2171
	Arcyptera meridionalis	Acrididae	NC_039962.1	15776	37	13	22	2	1051	14725	11191	1476	2058
	Xiphidiopsis gurneyi	Tettigoniidae	NC_039981.1	16225	37	13	22	2	1468	14757	11198	1515	2044
	Xizicus maculatus	Tettigoniidae	NC_040974.1	16358	37	13	22	2	1597	14761	11219	1454	2088
	Traulia nigritibialis	Acrididae	NC_041114.1	15701	37	13	22	2	915	14786	11188	1483	2115
	Stenocatantops splendens	Acrididae	NC_041115.1	15574	37	13	22	2	749	14825	11201	1474	2150
	Choroedocus capensis	Acrididae	NC_041116.1	16293	37	13	22	2	1434	14859	11211	1495	2153
	Xenogryllus marmoratus	Gryllidae	NC_041236.1	15762	38	13	23	2	1030	14732	11179	1433	2120
	Poecilimon luschani	Tettigoniidae	NC_042665.1	15568	37	13	22	2	904	14664	11159	1443	2062
	Isophya major	Tettigoniidae	NC_042666.1	15724	37	13	22	2	889	14835	11178	1483	2174
	Diabolocatantops pinguis	Acrididae	NC_042904.1	15604	37	13	22	2	829	14775	11183	1481	2111
	Oxya japonica	Acrididae	NC_043773.1	15427	37	13	22	2	598	14829	11189	1477	2163
	Ceracris fasciata fasciata	Acrididae	NC_043956.1	15569	37	13	22	2	810	14759	11192	1479	2088
	Conocephalus maculatus	Tettigoniidae	NC_045065.1	15905	37	13	22	2	1124	14781	11207	1459	2115
	Acosmetura nigrogeniculata	Tettigoniidae	NC_045212.1	16271	37	13	22	2	1487	14784	11218	1466	2100
	Euchorthippus unicolor	Acrididae	NC_045237.1	15629	37	13	22	2	1286	14343	11079	1445	1819
	Homoeoxipha nigripes	Trigonidiidae	NC_045841.1	15679	37	13	22	2	901	14778	11182	1479	2117
	Dianemobius fascipes	Trigonidiidae	NC_045846.1	15363	37	13	22	2	583	14780	11188	1479	2113
	Dianemobius furumagiensis	Trigonidiidae	NC_045847.1	15350	37	13	22	2	710	14640	11136	1435	2069
	Polionemobius taprobanensis	Trigonidiidae	NC_045848.1	16641	37	13	22	2	1892	14749	11190	1463	2096
	Oxya agavisa	Acrididae	NC_045883.1	15552	37	13	22	2	838	14714	11192	1456	2066
	Oxya hainanensis	Acrididae	NC_045928.1	15443	37	13	22	2	618	14825	11189	1469	2167
	Tagasta indica	Pyrgomorphidae	NC_045930.1	15531	37	13	22	2	773	14758	11180	1469	2109

Order	Species	Family	Reference (NCBI access number)	Genome Size	Numbers of					Total size			
					Nº Genes	Nº PCG	Nº tRNAs	Nº rRNAs	Non-coding portion	Coding portion	PCG	tRNA	rRNA
	Gesonula punctifrons	Acrididae	NC_046411.1	15432	37	13	22	2	608	14824	11212	1447	2165
	Tetrix ruyuanensis	Tetrigidae	NC_046412.1	15218	37	13	22	2	500	14718	11141	1453	2124
	Apalacris nigrogeniculata	Acrididae	NC_046527.1	15584	37	13	22	2	842	14742	11187	1463	2092
	Conophymacris viridis	Dericorythidae	NC_046528.1	15654	37	13	22	2	884	14770	11213	1458	2099
	Indopodisma kingdoni	Acrididae	NC_046529.1	15627	37	13	22	2	902	14725	11217	1446	2062
	Paratonkinacris vittifemoralis	Acrididae	NC_046530.1	15625	37	13	22	2	808	14817	11198	1471	2148
	Shirakiacris yunkweiensis	Acrididae	NC_046531.1	15655	37	13	22	2	854	14801	11191	1477	2133
	Spathosternum prasiniferum prasiniferum	Acrididae	NC_046532.1	15596	37	13	22	2	772	14824	11176	1482	2166
	Xiangelilacris zhongdianensis	Acrididae	NC_046533.1	15507	37	13	22	2	727	14780	11188	1479	2113
	Pseudoeoscyllina brevipennisoides	Acrididae	NC_046534.1	15629	37	13	22	2	810	14819	11193	1466	2160
	Bryodema nigroptera	Acrididae	NC_046535.1	15933	37	13	22	2	1088	14845	11225	1470	2150
	Bryodemella holdereri holdereri	Acrididae	NC_046536.1	15929	37	13	22	2	1117	14812	11183	1474	2155
	Heteropternis respondens	Acrididae	NC_046537.1	16265	37	13	22	2	1521	14744	11207	1449	2088
	Oedaleus abruptus	Acrididae	NC_046538.1	16251	37	13	22	2	1432	14819	11186	1490	2143
	Mecostethus alliaceus	Acrididae	NC_046539.1	16252	37	13	22	2	1519	14733	11180	1466	2087
	Ergatettix dorsifera	Tetrigidae	NC_046540.1	15326	37	13	22	2	520	14806	11189	1461	2156
	Euparatettix bimaculatus	Tetrigidae	NC_046541.1	15221	37	13	22	2	410	14811	11181	1483	2147
	Euparatettix variabilis	Tetrigidae	NC_046542.1	15194	37	13	22	2	292	14902	11210	1471	2221
	Bryodema dolichoptera	Acrididae	NC_046543.1	15924	37	13	22	2	1079	14845	11220	1471	2154
	Calliptamus barbarus	Acrididae	NC_046544.1	15578	37	13	22	2	545	15033	11188	1488	2357
	Fruhstorferiola omei	Acrididae	NC_046545.1	15639	37	13	22	2	825	14814	11194	1472	2148
	Sinopodisma funiushana	Acrididae	NC_046546.1	15894	37	13	22	2	1175	14719	11173	1460	2086
	Sinopodisma wudangshanensis	Acrididae	NC_046547.1	15834	37	13	22	2	1049	14785	11200	1475	2110

Order	Species	Family	Reference (NCBI access number)	Genome Size	Numbers of				Total size				
					Nº Genes	Nº PCG	Nº tRNAs	Nº rRNAs	Non-coding portion	Coding portion	PCG	tRNA	rRNA
	Ruidocollaris convexipennis	Tettigoniidae	NC_046548.1	16437	37	13	22	2	1710	14727	11189	1462	2076
	Sinopodisma lushiensis	Acrididae	NC_046549.1	15869	37	13	22	2	1138	14731	11149	1472	2110
	Sphingonotus menglaensis	Acrididae	NC_046550.1	15948	37	13	22	2	1111	14837	11181	1484	2172
	Traulia lofaoshana	Acrididae	NC_046551.1	15644	37	13	22	2	797	14847	11192	1482	2173
	Atractomorpha psittacina	Pyrgomorphidae	NC_046552.1	15567	37	13	22	2	794	14773	11189	1465	2119
	Bryodemacris uvarovi	Acrididae	NC_046553.1	15929	37	13	22	2	1091	14838	11219	1469	2150
	Bryodemella tuberculata diluta	Acrididae	NC_046554.1	15927	37	13	22	2	1090	14837	11219	1468	2150
	Dericorys annulata	Dericorythidae	NC_046555.1	15463	37	13	22	2	627	14836	11210	1467	2159
	Emeiacris maculata	Acrididae	NC_046556.1	15570	37	13	22	2	832	14738	11202	1455	2081
	Euthystira luteifemora	Acrididae	NC_046557.1	15608	37	13	22	2	1007	14601	11085	1436	2080
	Filchnerella qilianshanensis	Pamphagidae	NC_046558.1	15661	37	13	22	2	826	14835	11183	1484	2168
	Filchnerella tenggerensis	Pamphagidae	NC_046559.1	15659	37	13	22	2	823	14836	11183	1485	2168
	Omocestus viridulus	Acrididae	NC_046560.1	15635	37	13	22	2	859	14776	11172	1471	2133
	Pedopodisma emeiensis	Acrididae	NC_046561.1	15902	37	13	22	2	1125	14777	11204	1477	2096
	Sinopodisma lofaoshana	Acrididae	NC_046562.1	15867	37	13	22	2	1048	14819	11185	1479	2155
	Sphingonotus ningsianus	Acrididae	NC_046563.1	16261	37	13	22	2	1463	14798	11207	1486	2105
	Sphingonotus yenchihensis	Acrididae	NC_046564.1	16261	37	13	22	2	1438	14823	11201	1476	2146
	Traulia orchotibialis	Acrididae	NC_046565.1	15642	37	13	22	2	861	14781	11189	1479	2113
	Oxytauchira brachyptera	Acrididae	NC_046570.1	15445	37	13	22	2	512	14933	11231	1478	2224
	Anterastes babadaghi	Tettigoniidae	NC_046894.1	15883	37	13	22	2	1101	14782	11224	1453	2105
	Chorthippus fallax	Acrididae	NC_048465.1	16143	37	13	22	2	1338	14805	11172	1477	2156
	Shoveliteratura triangula	Tettigoniidae	NC_048466.1	16152	37	13	22	2	1346	14806	11185	1481	2140
	Natula pravdini	Trigonidiidae	NC_050742.1	15817	37	13	22	2	1130	14687	11194	1429	2064

Order	Species	Family	Reference (NCBI access number)	Genome Size	Numbers of				Total size				
					Nº Genes	Nº PCG	Nº tRNAs	Nº rRNAs	Non-coding portion	Coding portion	PCG	tRNA	rRNA
	Sinopodisma pieli	Acrididae	NC_051867.1	15625	37	13	22	2	821	14804	11185	1476	2143
	Anapodisma miramae	Acrididae	NC_052715.1	15189	37	13	22	2	406	14783	11200	1476	2107
	Sinopodisma rostellocerca	Acrididae	NC_052716.1	15622	37	13	22	2	785	14837	11185	1479	2173
	Stenocatantops mistshenkoi	Acrididae	NC_052717.1	15573	37	13	22	2	753	14820	11195	1474	2151
	Bryodema kozlovi	Acrididae	NC_052731.1	15930	37	13	22	2	1131	14799	11180	1470	2149
	Epacromius coerulipes	Acrididae	NC_052732.1	15592	37	13	22	2	741	14851	11185	1487	2179
	Filchnerella rubrimargina	Pamphagidae	NC_052733.1	15661	37	13	22	2	829	14832	11183	1482	2167
	Oedaleus manjius	Acrididae	NC_052734.1	15590	37	13	22	2	794	14796	11176	1471	2149
	Euconocephalus nasutus	Tettigoniidae	NC_053383.1	14999	37	13	22	2	209	14790	11172	1471	2147
	Svistella anhuiensis	Trigonidiidae	NC_053543.1	16494	37	13	22	2	1983	14511	11186	1478	1847
	Gryllus bimaculatus	Gryllidae	NC_053546.1	16075	37	13	22	2	1519	14556	11090	1447	2019
	Fer nigripennis	Acrididae	NC_053658.1	15669	37	13	22	2	1043	14626	11085	1444	2097
	Caryandoides hunanica	Acrididae	NC_053659.1	15555	37	13	22	2	784	14771	11201	1472	2098
	Paratoacris reticulipennis	Acrididae	NC_053660.1	16321	37	13	22	2	1513	14808	11180	1468	2160
	Oxytauchira flange	Acrididae	NC_053745.1	16250	37	13	22	2	1433	14817	11187	1473	2157
	Uvaroviola multispinosa	Acrididae	NC_053942.1	15620	37	13	22	2	753	14867	11209	1531	2127
	Eclipophleps carinata	Acrididae	NC_054195.1	15781	37	13	22	2	1010	14771	11215	1462	2094
	Sinopodisma qinlingensis	Acrididae	NC_056238.1	15843	37	13	22	2	1051	14792	11179	1472	2141
	Chorthippus parallelus parallelus	Acrididae	NC_056785.1	15623	37	13	22	2	778	14845	11188	1500	2157
	Chorthippus parallelus erythropus	Acrididae	NC_056786.1	15624	37	13	22	2	800	14824	11191	1472	2161
	Gryllus lineaticeps	Gryllidae	NC_057052.1	15607	37	13	22	2	1059	14548	11135	1443	1970
	Gryllus veletis	Gryllidae	NC_057053.1	15686	37	13	22	2	945	14741	11208	1469	2064
	Gryllodes sigillatus	Gryllidae	NC_057195.1	16369	37	13	22	2	1560	14809	11199	1472	2138

					Numbers of				Total size				
Order	Species	Family	Reference (NCBI access number)	Genome Size	Nº Genes	Nº PCG	Nº tRNAs	Nº rRNAs	Non-coding portion	Coding portion	PCG	tRNA	rRNA
	Tachycines zorzini	Rhaphidophoridae	NC_057442.1	15369	37	13	22	2	599	14770	11205	1460	2105
	Melanoplus differentialis	Acrididae	NC_057646.1	15625	37	13	22	2	817	14808	11177	1481	2150
	Average			15782.16	37.01	13.00	22.01	2.00	1014.85	14767.31	11185.54	1467.77	2114.00
	Standard deviation (SD)			411.75	0.09	0.00	0.09	0.00	420.04	99.34	53.29	18.28	69.09
	Coefficient of variation (CV)			0.03	0.00	0.00	0.00	0.00	0.41	0.01	0.00	0.01	0.03
	Amphinemura sulcicollis	Nemouridae	BK068636	15829	37	13	22	2	933	14896	11271	1478	2147
	Brachyptera seticornis	Taeniopterygidae	BK068637	15532	37	13	22	2	648	14884	11266	1479	2139
	Kathroperla doma	Chloroperlidae	BK068661	15990	37	13	22	2	1142	14848	11325	1474	2105
	Kathroperla siskiyou	Chloroperlidae	BK068662	16358	37	13	22	2	1585	14773	11250	1489	2100
	Leuctra hippopus	Leuctridae	BK068663	15685	37	13	22	2	827	14858	11319	1477	2129
	Paraperla wilsoni	Chloroperlidae	BK068646	16594	37	13	22	2	1684	14910	11295	1492	2123
	Siphonoperla torrentium	Chloroperlidae	BK068648	15996	37	13	22	2	1094	14902	11319	1486	2097
	Utaperla gaspesiana	Chloroperlidae	BK068655	17119	37	13	22	2	2212	14907	11307	1493	2107
	Utaperla lepnevae	Chloroperlidae	BK068656	16655	37	13	22	2	1719	14936	11274	1554	2108
	Utaperla sopladora	Chloroperlidae	BK068657	16278	37	13	22	2	1320	14958	11289	1555	2114
	Pteronarcys princeps	Pteronarcyidae	NC_006133.1	16004	37	13	22	2	1182	14822	11221	1480	2121
	Dinocras cephalotes	Perlidae	NC_022843.1	15666	37	13	22	2	735	14931	11215	1494	2222
	Acroneuria hainana	Perlidae	NC_026104.1	15804	37	13	22	2	907	14897	11202	1487	2208
	Apteroperla tikumana	Capniidae	NC_027698.1	15564	37	13	22	2	787	14777	11110	1476	2191
	Kamimuria chungnanshana	Perlidae	NC_028076.1	15943	37	13	22	2	1065	14878	11217	1488	2173
	Pteronarcella badia	Pteronarcyidae	NC_029248.1	15585	37	13	22	2	826	14759	11226	1483	2050
	Capnia zijinshana	Capniidae	NC_034661.1	16310	37	13	22	2	1493	14817	11226	1476	2115
	Styloperla spinicercia	Styloperlidae	NC_034809.1	16129	37	13	22	2	1277	14852	11234	1479	2139
Plecoptera	*Nemoura nankinensis*	Nemouridae	NC_034939.1	16602	37	13	22	2	1781	14821	11229	1475	2117

Order	Species	Family	Reference (NCBI access number)	Genome Size	Numbers of				Total size				
					Nº Genes	Nº PCG	Nº tRNAs	Nº rRNAs	Non-coding portion	Coding portion	PCG	tRNA	rRNA
	Zelandoperla fenestrata	Gripopterygidae	NC_034997.1	16385	37	13	22	2	1582	14803	11180	1490	2133
	Suwallia errata	Chloroperlidae	NC_037754.1	16146	37	13	22	2	1284	14862	11246	1487	2129
	Taeniopteryx ugola	Taeniopterygidae	NC_037897.1	15353	37	13	22	2	540	14813	11223	1471	2119
	Isoperla eximia	Perlodidae	NC_038167.1	16034	37	13	22	2	1175	14859	11258	1479	2122
	Pseudomegarcys japonica	Perlodidae	NC_038168.1	16067	37	13	22	2	1203	14864	11264	1479	2121
	Soliperla sp. ZTC-2018	Peltoperlidae	NC_038189.1	15877	37	13	22	2	1038	14839	11262	1477	2100
	Isoperla bilineata	Perlodidae	NC_038190.1	15048	37	13	22	2	148	14900	11258	1481	2161
	Scopura longa	Scopuridae	NC_041105.1	15798	37	13	22	2	969	14829	11227	1467	2135
	Antarctoperla michaelseni	Gripopterygidae	NC_042199.1	16069	37	13	22	2	1285	14784	11213	1447	2124
	Neuroperla schedingi	Eustheniidae	NC_042200.1	16882	37	13	22	2	2025	14857	11222	1478	2157
	Diamphipnoa annulata	Diamphipnoidae	NC_042205.1	14882	37	13	22	2	49	14833	11222	1475	2136
	Neonemura barrosi	Notonemouridae	NC_042206.1	14852	37	13	22	2	52	14800	11216	1460	2124
	Rhopalopsole bulbifera	Leuctridae	NC_042207.1	15599	37	13	22	2	693	14906	11208	1481	2217
	Mesonemoura metafiligera	Nemouridae	NC_044719.1	15739	37	13	22	2	940	14799	11217	1463	2119
	Mesonemoura tritaenia	Nemouridae	NC_044720.1	15778	37	13	22	2	955	14823	11229	1471	2123
	Amphinemura longispina	Nemouridae	NC_044748.1	15709	37	13	22	2	866	14843	11229	1484	2130
	Amphinemura yao	Nemouridae	NC_044749.1	15876	37	13	22	2	1044	14832	11229	1477	2126
	Indonemoura jacobsoni	Nemouridae	NC_044750.1	15642	37	13	22	2	832	14810	11223	1475	2112
	Indonemoura nohirae	Nemouridae	NC_044751.1	15738	37	13	22	2	920	14818	11229	1472	2117
	Protonemura kohnoae	Nemouridae	NC_044752.1	15707	37	13	22	2	885	14822	11229	1474	2119
	Protonemura orbiculata	Nemouridae	NC_044753.1	15758	37	13	22	2	935	14823	11229	1475	2119
	Sphaeronemoura grandicauda	Nemouridae	NC_044754.1	15661	37	13	22	2	837	14824	11229	1472	2123
	Sphaeronemoura acutispina	Nemouridae	NC_044755.1	15016	37	13	22	2	185	14831	11229	1473	2129
	Protonemura meyeri	Nemouridae	NC_050322.1	15695	37	13	22	2	899	14796	11203	1468	2125

					Numbers of				Total size				
Order	Species	Family	Reference (NCBI access number)	Genome Size	Nº Genes	Nº PCG	Nº tRNAs	Nº rRNAs	Non-coding portion	Coding portion	PCG	tRNA	rRNA
	Paraleuctra cercia	Leuctridae	NC_053557.1	15625	37	13	22	2	827	14798	11205	1469	2124
	Perlomyia isobeae	Leuctridae	NC_053558.1	15795	37	13	22	2	975	14820	11207	1472	2141
	Acroneuria carolinensis	Perlidae	NC_053852.1	15718	37	13	22	2	843	14875	11225	1487	2163
	Togoperla limbata	Perlidae	NC_053853.1	15915	37	13	22	2	1015	14900	11214	1500	2186
	Oyamia nigribasis	Perlidae	NC_056285.1	15923	37	13	22	2	1025	14898	11229	1491	2178
	Amphinemura bulla	Nemouridae	NC_057056.1	15827	37	13	22	2	995	14832	11229	1484	2119
	Paragnetina indentata	Perlidae	NC_057280.1	15885	37	13	22	2	968	14917	11223	1488	2206
	Perlesta teaysia	Perlidae	NC_057281.1	16023	37	13	22	2	1205	14818	11223	1473	2122
	Flavoperla hatakeyamae	Perlidae	NC_057436.1	15730	37	13	22	2	850	14880	11234	1478	2168
	Sphaeronemoura elephas	Nemouridae	NC_057512.1	15846	37	13	22	2	1026	14820	11220	1475	2125
	Nemoura meniscata	Nemouridae	NC_057513.1	15895	37	13	22	2	1087	14808	11217	1475	2116
	Average			15872.89	36.98	13.00	21.98	2.00	1025.44	14847.44	11235.09	1477.67	2134.69
	Standard deviation (SD)			432.94	0.31	0.00	0.31	0.00	424.29	45.77	35.97	22.65	32.53
	Coefficient of variation (CV)			0.03	0.01	0.00	0.01	0.00	0.41	0.00	0.00	0.02	0.02
	Medauroidea extradentata	Phasmatidae	BK068643	17428	37	13	22	2	2823	14605	11217	1391	1997
	Timema bartmani	Timematidae	BK068649	19048	37	13	22	2	4253	14795	11208	1502	2085
	Timema genevievae	Timematidae	BK068650	18085	37	13	22	2	3321	14764	11172	1502	2090
	Timema monikensis	Timematidae	BK068651	16977	37	13	22	2	2054	14923	11235	1575	2113
	Timema podura	Timematidae	BK068652	16864	37	13	22	2	2044	14820	11205	1503	2112
	Timema poppensis	Timematidae	BK068664	18305	37	13	21	2	3457	14848	11268	1407	2173
	Timema shepardi	Timematidae	BK068665	17534	37	13	22	2	2671	14863	11260	1443	2160
	Entoria okinawaensis	Phasmatidae	NC_014694.1	16910	37	13	22	2	2291	14619	11101	1452	2066
	Eurycantha calcarata	Phasmatidae	NC_058255.1	16280	37	13	22	2	1689	14591	11095	1440	2056
Phasmatodea	*Extatosoma tiaratum*	Phasmatidae	NC_017748.1	16537	37	13	22	2	1899	14638	11099	1463	2076

Order	Species	Family	Reference (NCBI access number)	Genome Size	Numbers of				Total size				
					Nº Genes	Nº PCG	Nº tRNAs	Nº rRNAs	Non-coding portion	Coding portion	PCG	tRNA	rRNA
	Heteropteryx dilatata	Heteropterygidae	NC_014680.1	16618	37	13	22	2	1965	14653	11098	1474	2081
	Megacrania alpheus adan	Phasmatidae	NC_014688.1	17124	37	13	22	2	2507	14617	11112	1440	2065
	Micadina phluctainoides	Lonchodidae	NC_014673.1	16507	37	13	22	2	1896	14611	11103	1460	2048
	Phobaeticus serratipes	Phasmatidae	NC_014678.1	16182	37	13	22	2	1617	14565	11068	1453	2044
	Phraortes illepidus	Lonchodidae	NC_014695.1	16456	37	13	22	2	1900	14556	11066	1447	2043
	Phraortes sp. Iriomote Island	Lonchodidae	NC_014705.1	16867	37	13	22	2	2341	14526	11053	1439	2034
	Ramulus hainanense	Phasmatidae	NC_013185.1	15590	40	13	25	2	760	14830	11081	1680	2069
	Ramulus mikado	Phasmatidae	NC_014702.1	16633	37	13	22	2	2007	14626	11107	1447	2072
	Average			16996.94	37.17	13.00	22.11	2.00	2305.28	14691.67	11141.56	1473.22	2076.89
	Standard deviation (SD)			833.58	0.71	0.00	0.76	0.00	792.25	124.72	71.75	65.80	42.71
	Coefficient of variation (CV)			0.05	0.02	0.00	0.03	0.00	0.34	0.01	0.01	0.04	0.02
