# Supplementary material for: Expanding the mitochondrial genomic toolkit for Polyneoptera: New mitogenomes and evaluation of reduced marker sets for phylogeny and DNA barcoding
Source: Genet Mol Biol. 2026 Jul 24;49(3):e20250282. doi: 10.1590/1678-4685-GMB-2025-0282 (PMC13403772; doi:10.1590/1678-4685-GMB-2025-0282)
Supplement: Table S7 - [file 1415-4757-GMB-49-3-e20250282-s7.pdf]

Genetics and Molecular Biology

DOI: https://doi.org/10.1590/1678-4685-GMB-2025-0282

Supplementary Material to "Expanding the mitochondrial genomic toolkit for Polyneoptera: New mitogenomes and evaluation of reduced marker sets for phylogeny and DNA barcoding"

Table S7 - Results of barcoding gap analyses for Orthoptera, Blattodea, Mantodea, and Plecoptera based on mitochondrial gene regions. The table summarizes intraspecific and interspecific genetic distances (minimum, median, and maximum), the difference between minimum interspecific and maximum intraspecific distances, and the results of Mann–Whitney–Wilcoxon tests comparing distance distributions.

Order	Gene	NCBI Gene ID	Region	Length (bp)	Number of species	Number of sequences	Interspecific distance			Intraspecific distance			(Min. inter. dist.) - (Max. intra. dist.)	Mann-Whitney-Wilcoxon	
							Min	Median	Max	Min	Median	Max		W	p-value
	12S	19893562	380..688	301	19	93	0.00	0.28	1.07	0.00	0.00	0.97	-96.97%	17098394	2.20E-16
	16S	19893560	750..1101	354	36	165	0.00	0.23	0.37	0.00	0.00	0.19	-19.20%	187786250	2.20E-16
	ATP6	19893539	1..675	678	5	24	0.08	0.19	0.39	0.00	0.00	0.03	4.68%	42320	2.20E-16
	COX1	19893533	97..660	564	39	278	0.00	0.22	0.37	0.00	0.02	0.29	-29.29%	1495162298	2.20E-16
	CYTB	19893556	430..854	440	23	120	0.00	0.22	0.38	0.00	0.01	0.24	-23.61%	50291666	2.20E-16
	ND2	19893529	58..1014	952	32	159	0.00	0.46	0.70	0.00	0.03	0.31	-31.42%	147852038	2.20E-16
	ND4	19893551	1..1336	1335	3	14	0.13	0.15	0.17	0.00	0.01	0.05	7.87%	3380	2.20E-16
**Orthoptera**	*ND5*	19893549	815..1413	627	31	164	0.00	0.12	0.24	0.00	0.01	0.08	-7.89%	158227725	2.20E-16
	ATP6	19893539	22..675	660	26	138	0.09	0.28	0.48	0.00	0.01	0.15	-6.77%	74919153	2.20E-16
	COX1	19893533	130..637	508	55	360	0.00	0.23	0.40	0.00	0.01	0.17	-16.74%	3988399496	2.20E-16
	COX2	19893535	59..608	550	52	364	0.00	0.28	0.45	0.00	0.00	0.16	-15.96%	4049518131	2.20E-16
	ND5	19893549	1..1717	1720	14	63	0.09	0.37	0.53	0.00	0.01	0.06	2.68%	2770196	2.20E-16
**Blattodea**	*ND6*	19893555	1..525	489	14	63	0.08	0.25	0.37	0.00	0.00	0.08	0.41%	2770196	2.20E-16
	16S	19893560	1..1322	1314	3	12	0.11	0.14	0.16	0.00	0.02	0.04	6.51%	1728	5.92E-15
	ATP8	19893538	1..159	159	3	12	0.24	0.26	0.36	0.00	0.03	0.14	9.53%	1728	5.40E-15
**Mantodea**	*COX1*	19893533	1..1466	1536	3	12	0.13	0.15	0.17	0.00	0.02	0.06	7.40%	1728	5.92E-15

Order	Gene	NCBI Gene ID	Region	Length (bp)	Number of species	Number of sequences	Interspecific distance			Intraspecific distance			(Min. inter. dist.) - (Max. intra. dist.)	Mann-Whitney-Wilcoxon	
							Min	Median	Max	Min	Median	Max		W	p-value
	ND2	19893529	22..1024	1027	3	12	0.17	0.24	0.25	0.00	0.02	0.12	4.84%	1728	5.93E-15
	ND6	19893555	4..522	504	3	12	0.17	0.20	0.25	0.00	0.03	0.08	8.69%	1728	5.86E-15
	COX1	19893533	95..668	573	12	70	0.16	0.24	0.30	0.00	0.01	0.20	-3.74%	4815396	2.20E-16
	ND4	19893551	1..1341	1341	3	14	0.19	0.22	0.24	0.00	0.06	0.08	10.97%	3380	2.20E-16
	ND4L	19893552	1..297	297	3	17	0.20	0.22	0.28	0.00	0.04	0.09	11.23%	7680	2.20E-16
	ND5	19893549	55..1717	1662	3	14	0.39	0.42	0.46	0.00	0.07	0.13	25.55%	4588	2.20E-16
**Plecoptera**	*ND6*	19893555	22..525	503	3	15	0.20	0.23	0.25	0.00	0.05	0.07	12.41%	3380	2.20E-16
**Phasmatodea**	*COX1*	19893533	819..1294	476	11	87	0	0.142731865	0.269552803	0	0.006342533	0.047712637	-4.77%	10744453	2.20E-16
