# Supplementary material for: Expanding the mitochondrial genomic toolkit for Polyneoptera: New mitogenomes and evaluation of reduced marker sets for phylogeny and DNA barcoding
Source: Genet Mol Biol. 2026 Jul 24;49(3):e20250282. doi: 10.1590/1678-4685-GMB-2025-0282 (PMC13403772; doi:10.1590/1678-4685-GMB-2025-0282)
Supplement: Table S8 - [file 1415-4757-GMB-49-3-e20250282-s8.pdf]

Genetics and Molecular Biology

DOI: https://doi.org/10.1590/1678-4685-GMB-2025-0282

Supplementary Material to "Expanding the mitochondrial genomic toolkit for Polyneoptera: New mitogenomes and evaluation of reduced marker sets for phylogeny and DNA barcoding"

Table S8 - Mantel and Robinson–Foulds (RF) coefficients comparing phylogenetic trees inferred from different mitochondrial datasets in Plecoptera.

	**Mantel**				**RF**			
**Dataset**	**mt DNA**	**PCG**	**Partition**	**PCG _3rd**	**mt DNA**	**PCG**	**Partition**	**PCG _3rd**
mtDNA	1.000	1.000	0.999	0.998	0.000	0.222	0.204	0.204
PCG	1.000	1.000	0.999	0.999	0.222	0.000	0.148	0.167
Partition	0.999	0.999	1.000	0.999	0.204	0.148	0.000	0.148
var	0.997	0.998	0.997	0.996	0.148	0.148	0.222	0.222
COX1	0.995	0.995	0.996	0.994	0.500	0.537	0.463	0.519
COX1_var	0.998	0.999	0.998	0.997	0.185	0.204	0.148	0.185
PCG_3rd	0.998	0.999	0.999	1.000	0.204	0.167	0.148	0.000
var_3rd	0.998	0.998	0.998	0.997	0.185	0.222	0.167	0.148
COX1_3rd	0.970	0.971	0.972	0.974	0.611	0.574	0.611	0.611
COX1_var_3rd	0.998	0.998	0.999	0.998	0.148	0.185	0.074	0.130
ATP6_3rd	0.985	0.982	0.980	0.979	0.796	0.778	0.778	0.796
COX3_3rd	0.993	0.993	0.994	0.995	0.537	0.519	0.519	0.519
ND4_3rd	0.997	0.998	0.998	0.997	0.278	0.259	0.315	0.296
ND4L_3rd	0.921	0.924	0.925	0.922	0.870	0.833	0.852	0.852
ND5_3rd	0.998	0.999	0.998	0.997	0.370	0.315	0.315	0.296
ND6_3rd	0.965	0.967	0.971	0.973	0.389	0.389	0.407	0.389
16s	0.973	0.974	0.977	0.976	0.333	0.389	0.278	0.352
ATP6	0.993	0.994	0.994	0.992	0.574	0.556	0.574	0.556
COX3	0.992	0.992	0.994	0.994	0.389	0.389	0.389	0.389
ND4	0.999	0.999	0.999	0.997	0.278	0.296	0.315	0.333
ND4L	0.993	0.993	0.993	0.992	0.741	0.704	0.704	0.722
ND5	0.995	0.996	0.995	0.994	0.333	0.241	0.333	0.315
ND6	0.987	0.989	0.990	0.991	0.426	0.370	0.407	0.370

* Dataset definitions: mtDNA, complete mitochondrial genome; PCG, concatenated mitochondrial protein-coding genes; Partition, protein-coding genes analyzed under a partitioned scheme; var, mitochondrial regions identified as nucleotide-diversity hotspots; COX1_var, variable regions plus the COX1 gene; _3rd, datasets including only third codon positions of protein-coding genes.
