# Supplementary material for: Expanding the mitochondrial genomic toolkit for Polyneoptera: New mitogenomes and evaluation of reduced marker sets for phylogeny and DNA barcoding
Source: Genet Mol Biol. 2026 Jul 24;49(3):e20250282. doi: 10.1590/1678-4685-GMB-2025-0282 (PMC13403772; doi:10.1590/1678-4685-GMB-2025-0282)
Supplement: Table S9 - [file 1415-4757-GMB-49-3-e20250282-s9.pdf]

Genetics and Molecular Biology
DOI: https://doi.org/10.1590/1678-4685-GMB-2025-0282

Supplementary Material to "Expanding the mitochondrial genomic toolkit for Polyneoptera: New mitogenomes and evaluation of reduced marker sets for phylogeny and DNA barcoding"

Table S9 - Mantel and Robinson–Foulds (RF) coefficients comparing phylogenetic trees inferred from different mitochondrial datasets in Orthoptera.

	Mantel				RF			
	**mt DNA**	**PCG**	**Partition**	**PCG_3rd**	**mt DNA**	**PCG**	**Partition**	**PCG_3rd**
mtDNA	1.000	0.992	0.992	0.973	0.000	0.137	0.133	0.190
PCG	0.992	1.000	0.999	0.980	0.137	0.000	0.028	0.169
Partition	0.992	0.999	1.000	0.984	0.133	0.028	0.000	0.169
var	0.979	0.992	0.993	0.988	0.181	0.116	0.124	0.185
COX1	0.947	0.943	0.942	0.909	0.379	0.353	0.353	0.386
COX1_var	0.988	0.997	0.997	0.985	0.169	0.100	0.112	0.201
PCG_3rd	0.973	0.980	0.984	1.000	0.190	0.169	0.169	0.000
var_3rd	0.957	0.969	0.975	0.993	0.274	0.273	0.277	0.229
COX1_3rd	0.900	0.902	0.907	0.925	0.540	0.534	0.538	0.550
COX1_var_3rd	0.962	0.972	0.976	0.996	0.258	0.233	0.241	0.225
ATP6_3rd	0.885	0.887	0.893	0.917	0.581	0.550	0.550	0.566
COX2_3rd	0.899	0.890	0.894	0.909	0.565	0.554	0.550	0.554
ND2_3rd	0.598	0.666	0.669	0.686	0.448	0.454	0.454	0.438
ND4_3rd	0.899	0.928	0.933	0.946	0.435	0.426	0.422	0.426
ND4L_3rd	0.822	0.818	0.823	0.840	0.690	0.695	0.699	0.699
ND5_3rd	0.913	0.932	0.937	0.962	0.419	0.410	0.418	0.398
ATP6	0.941	0.937	0.939	0.927	0.440	0.422	0.418	0.422
COX2	0.934	0.929	0.932	0.931	0.423	0.410	0.406	0.434
ND2	0.612	0.680	0.682	0.692	0.347	0.321	0.329	0.333
ND4	0.922	0.944	0.947	0.953	0.355	0.357	0.361	0.386
ND4L	0.812	0.809	0.819	0.845	0.665	0.659	0.659	0.651
ND5	0.934	0.952	0.956	0.969	0.367	0.329	0.341	0.341

* Dataset definitions: mtDNA, complete mitochondrial genome; PCG, concatenated mitochondrial protein-coding genes; Partition, protein-coding genes analyzed under a partitioned scheme; var, mitochondrial regions identified as nucleotide-diversity hotspots; COX1_var, variable regions plus the COX1 gene; _3rd, datasets including only third codon positions of protein-coding genes.
