# Supplementary material for: Expanding the mitochondrial genomic toolkit for Polyneoptera: New mitogenomes and evaluation of reduced marker sets for phylogeny and DNA barcoding
Source: Genet Mol Biol. 2026 Jul 24;49(3):e20250282. doi: 10.1590/1678-4685-GMB-2025-0282 (PMC13403772; doi:10.1590/1678-4685-GMB-2025-0282)
Supplement: Table S10 - [file 1415-4757-GMB-49-3-e20250282-s10.pdf]

Genetics and Molecular Biology
DOI: https://doi.org/10.1590/1678-4685-GMB-2025-0282

Supplementary Material to "Expanding the mitochondrial genomic toolkit for Polyneoptera: New mitogenomes and evaluation of reduced marker sets for phylogeny and DNA barcoding"

Table S10 - Mantel and Robinson–Foulds (RF) coefficients comparing phylogenetic trees inferred from different mitochondrial datasets in Phasmatodea.

	Mantel				RF			
**Dataset**	**mt DNA**	**PCG**	**Partition**	**PCG_3rd**	**mt DNA**	**PCG**	**Partition**	**PCG_3rd**
mtDNA	1.000	0.998	0.994	0.971	0.000	0.111	0.167	0.167
PCG	0.998	1.000	0.997	0.970	0.111	0.000	0.167	0.167
Partition	0.994	0.997	1.000	0.978	0.167	0.167	0.000	0.056
var	0.995	0.998	0.999	0.973	0.167	0.167	0.111	0.056
COX1	0.971	0.979	0.981	0.958	0.500	0.500	0.500	0.500
COX1_var	0.993	0.997	0.997	0.967	0.167	0.111	0.167	0.111
PCG_3rd	0.971	0.970	0.978	1.000	0.167	0.167	0.056	0.000
var_3rd	0.986	0.987	0.992	0.992	0.222	0.167	0.222	0.167
COX1_3rd	0.922	0.928	0.936	0.977	0.611	0.611	0.611	0.611
COX1_var_3rd	0.976	0.978	0.984	0.995	0.222	0.167	0.222	0.167
ATP8_3rd	0.915	0.919	0.920	0.862	0.778	0.778	0.778	0.778
ND2_3rd	0.990	0.991	0.989	0.981	0.167	0.167	0.278	0.278
ND4L_3rd	0.932	0.939	0.948	0.977	0.333	0.333	0.278	0.278
ND5_3rd	0.952	0.949	0.957	0.993	0.278	0.278	0.111	0.167
16S	0.975	0.976	0.973	0.913	0.167	0.167	0.111	0.056
ATP8	0.930	0.930	0.924	0.869	0.611	0.611	0.611	0.611
ND2	0.981	0.984	0.979	0.973	0.333	0.333	0.444	0.444
ND4L	0.947	0.955	0.964	0.982	0.389	0.389	0.333	0.333
ND5	0.995	0.996	0.998	0.979	0.222	0.222	0.056	0.111

* Dataset definitions: mtDNA, complete mitochondrial genome; PCG, concatenated mitochondrial protein-coding genes; Partition, protein-coding genes analyzed under a partitioned scheme; var, mitochondrial regions identified as nucleotide-diversity hotspots; COX1_var, variable regions plus the COX1 gene; _3rd, datasets including only third codon positions of protein-coding genes.
