# Supplementary material for: Expanding the mitochondrial genomic toolkit for Polyneoptera: New mitogenomes and evaluation of reduced marker sets for phylogeny and DNA barcoding
Source: Genet Mol Biol. 2026 Jul 24;49(3):e20250282. doi: 10.1590/1678-4685-GMB-2025-0282 (PMC13403772; doi:10.1590/1678-4685-GMB-2025-0282)
Supplement: Table S11 - [file 1415-4757-GMB-49-3-e20250282-s11.pdf]

Genetics and Molecular Biology
DOI: https://doi.org/10.1590/1678-4685-GMB-2025-0282

Supplementary Material to "Expanding the mitochondrial genomic toolkit for Polyneoptera: New mitogenomes and evaluation of reduced marker sets for phylogeny and DNA barcoding"

Table S11 - Mantel and Robinson–Foulds (RF) coefficients comparing phylogenetic trees inferred from different mitochondrial datasets in Mantodea.

	Mantel				RF			
**Dataset**	**mt DNA**	**PCG**	**Partition**	**PCG_3rd**	**mt DNA**	**PCG**	**Partition**	**PCG_3rd**
mtDNA	1.000	0.997	0.996	0.988	0	0.08	0.08	0.12
PCG	0.997	1.000	1.000	0.990	0.08	0	0	0.12
Partition	0.996	1.000	1.000	0.991	0.08	0	0	0.12
var	0.993	0.995	0.995	0.985	0.2	0.12	0.12	0.2
COX1	0.951	0.956	0.954	0.932	0.48	0.52	0.52	0.4
COX1_var	0.993	0.996	0.996	0.984	0.24	0.2	0.2	0.32
PCG_3rd	0.988	0.990	0.991	1.000	0.12	0.12	0.12	0
var_3rd	0.992	0.994	0.994	0.991	0.28	0.24	0.24	0.32
COX1_3rd	0.960	0.963	0.964	0.975	0.48	0.52	0.52	0.52
COX1_var_3rd	0.992	0.993	0.993	0.995	0.32	0.36	0.36	0.36
ATP8_3rd	0.633	0.632	0.629	0.568	0.88	0.84	0.84	0.88
ND2_3rd	0.984	0.987	0.987	0.984	0.56	0.56	0.56	0.6
ND6_3rd	0.960	0.962	0.963	0.979	0.72	0.72	0.72	0.76
16s	0.985	0.986	0.986	0.975	0.24	0.24	0.24	0.28
ATP8	0.720	0.718	0.715	0.658	0.76	0.76	0.76	0.8
ND2	0.982	0.986	0.986	0.975	0.56	0.56	0.56	0.56
ND6	0.966	0.968	0.970	0.984	0.64	0.6	0.6	0.6

* Dataset definitions: mtDNA, complete mitochondrial genome; PCG, concatenated mitochondrial protein-coding genes; Partition, protein-coding genes analyzed under a partitioned scheme; var, mitochondrial regions identified as nucleotide-diversity hotspots; COX1_var, variable regions plus the COX1 gene; _3rd, datasets including only third codon positions of protein-coding genes.
