# Supplementary material for: Expanding the mitochondrial genomic toolkit for Polyneoptera: New mitogenomes and evaluation of reduced marker sets for phylogeny and DNA barcoding
Source: Genet Mol Biol. 2026 Jul 24;49(3):e20250282. doi: 10.1590/1678-4685-GMB-2025-0282 (PMC13403772; doi:10.1590/1678-4685-GMB-2025-0282)
Supplement: Table S12 - [file 1415-4757-GMB-49-3-e20250282-s12.pdf]

Genetics and Molecular Biology
DOI: https://doi.org/10.1590/1678-4685-GMB-2025-0282

Supplementary Material to "Expanding the mitochondrial genomic toolkit for Polyneoptera: New mitogenomes and evaluation of reduced marker sets for phylogeny and DNA barcoding"

Table S12 - Mantel and Robinson–Foulds (RF) coefficients comparing phylogenetic trees inferred from different mitochondrial datasets in Blatoddea.

	Mantel				RF			
**Dataset**	**mt DNA**	**PCG**	**Partition**	**PCG_3rd**	**mt DNA**	**PCG**	**Partition**	**PCG_3rd**
mtDNA	1.000	0.982	0.994	0.984	0.000	0.164	0.150	0.230
PCG	0.982	1.000	0.988	0.966	0.164	0.000	0.066	0.221
Partition	0.994	0.988	1.000	0.990	0.150	0.066	0.000	0.208
var	0.979	0.997	0.989	0.969	0.208	0.159	0.195	0.239
COX1	0.974	0.982	0.983	0.962	0.412	0.376	0.358	0.447
COX1_var	0.980	0.998	0.990	0.968	0.208	0.142	0.168	0.243
PCG_3rd	0.984	0.966	0.990	1.000	0.230	0.221	0.208	0.000
var_3rd	0.982	0.958	0.986	0.996	0.301	0.288	0.292	0.239
COX1_3rd	0.974	0.982	0.983	0.962	0.412	0.376	0.358	0.447
COX1_var_3rd	0.981	0.965	0.988	0.998	0.319	0.301	0.301	0.248
ATP6_3rd	0.925	0.883	0.919	0.946	0.637	0.628	0.624	0.624
ND5_3rd	0.967	0.933	0.966	0.983	0.460	0.456	0.456	0.434
ND2_3rd	0.952	0.951	0.971	0.974	0.487	0.500	0.504	0.469
ND6_3rd	0.965	0.938	0.962	0.972	0.562	0.540	0.540	0.580
ND5	0.979	0.958	0.984	0.980	0.363	0.323	0.336	0.367
ATP6	0.959	0.939	0.957	0.959	0.438	0.420	0.420	0.425
ND2	0.954	0.962	0.973	0.963	0.305	0.296	0.305	0.345
ND6	0.969	0.981	0.970	0.954	0.482	0.434	0.456	0.487

* Dataset definitions: mtDNA, complete mitochondrial genome; PCG, concatenated mitochondrial protein-coding genes; Partition, protein-coding genes analyzed under a partitioned scheme; var, mitochondrial regions identified as nucleotide-diversity hotspots; COX1_var, variable regions plus the COX1 gene; _3rd, datasets including only third codon positions of protein-coding genes.
